# Alveolar epithelial paxillin in postnatal lung alveolar development

**DOI:** 10.1242/bio.061939

**Published:** 2025-03-20

**Authors:** Mikaela Scheer, Priscilla Kyi, Tadanori Mammoto, Akiko Mammoto

**Affiliations:** ^1^ Department of Pediatrics, Medical College of Wisconsin, Milwaukee, WI 53226, USA; ^2^ Department of Cell Biology, Neurobiology and Anatomy, Medical College of Wisconsin, Milwaukee, WI 53226, USA; ^3^ Department of Pharmacology and Toxicology, Medical College of Wisconsin, Milwaukee, WI 53226, USA

**Keywords:** Lung development, Paxillin, ABCA3, CEBPA

## Abstract

Focal adhesion protein, paxillin plays an important role in embryonic development. We have reported that paxillin controls directional cell motility and angiogenesis. The role of paxillin in lung development remains unclear. Paxillin expression is higher in mouse pulmonary alveolar epithelial type 2 (AT2) cells at postnatal day (P)10 (alveolar stage) compared to P0 (saccular stage). The alveolar and vascular structures are disrupted, lung compliance is reduced, and the postnatal survival rate is lower in tamoxifen-induced *Pxn^i^*^Δ*AT2*^ neonatal mice, in which the levels of paxillin in AT2 cells are knocked down. Surfactant protein expression and lamellar body structure are also inhibited in *Pxn^i^*^Δ*AT2*^ neonatal mouse lungs. The expression of lipid transporter ABCA3 and its transcriptional regulator CEBPA that control surfactant homeostasis is inhibited in *Pxn^i^*^Δ*AT2*^ neonatal mouse AT2 cells. These findings suggest that paxillin controls lung alveolar development through CEBPA-ABCA3 signaling in AT2 cells. Modulation of paxillin in AT2 cells may be novel interventions for neonatal lung developmental disorder.

## Teaser

**Summary:** Epithelial cell paxillin is necessary for the postnatal lung alveolar development, which is mediated by CEBPA-ABCA3 signaling to control surfactant homeostasis.

## INTRODUCTION

Impairment of lung development during transition from saccular to alveolar stage is one of the most significant features of neonatal lung developmental disorders, including bronchopulmonary dysplasia (BPD) (Thebaud, 2007). AT2 cells produce and secrete pulmonary surfactant proteins and lipids into the alveoli, which reduces alveolar surface tension and prevents them from collapse to promote gas exchange (Whitsett et al., 2015). AT2 cells also secrete angiogenic factors (e.g. angiopoietin1), which stimulates blood vessel formation and nutrient transport (Mammoto and Mammoto, 2019; Whitsett et al., 2015). In addition, AT2 cells serve as primary progenitors to maintain alveolar epithelial structures, repairing the alveolar epithelium after injury (Barkauskas et al., 2013; Hogan et al., 2014; Konkimalla et al., 2023; Whitsett et al., 2015). Maintaining the functional AT2 cells in the lung during this critical period could be a promising strategy for lung developmental disorders in preterm babies.

Surfactant proteins synthesized by AT2 cells and surfactant lipids are packaged and stored in lamellar bodies (LBs), multi-lamellated storage forms of pulmonary surfactant, maintaining surface tension to prevent the collapse of alveoli (Chander and Fisher, 1990; de Aguiar Vallim et al., 2017; Dietl et al., 2001; Frick et al., 2001; Perez-Gil and Weaver, 2010; Weaver et al., 2002; Whitsett et al., 2015). Disruption of surfactant homeostasis is a hallmark of neonatal lung diseases (Whitsett et al., 2015); defects in surfactant homeostasis are accompanied by a disruption of LB ultrastructure, leading to lung developmental disorder and inhibiting repair from injury (Dillard et al., 2020; Guenthart et al., 2019; Perez-Gil and Weaver, 2010). The ATP binding cassette (ABC) transmembrane transporter A3 (ABCA3) is expressed in AT2 cells to transport surfactant lipids [e.g. phosphatidylcholine (PC), phosphatidylglycerol (PG), phosphatidylethanolamine, sphingolipids, cholesterol] into LBs, and is required for LB formation and surfactant homeostasis during lung development (Ban et al., 2007; Mulugeta et al., 2002; Rindler et al., 2017). The levels of ABCA3 increase in the developing fetal lungs (Stahlman et al., 2007); in contrast, loss of ABCA3 in AT2 cells results in early postnatal death due to impairment of packaging surfactant into LBs and secreting into alveoli (Ban et al., 2007; Besnard et al., 2007; Shulenin et al., 2004). ABCA3-related pulmonary disease patients reveal abnormalities in surfactant lipid content and function (Garmany et al., 2006). A transcription factor, CCAAT enhancer binding protein A (CEBPA) controls ABCA3 expression, which is necessary for tissue development and lipid metabolism (Cao et al., 1991; Sugahara et al., 2001). CEBP is highly expressed in AT2 cells in the fetal lungs (Li et al., 1995); in contrast, knockdown of CEBPA in AT2 cells inhibits maturation of the fetal lungs, leading to respiratory failure at birth (Martis et al., 2006).

Mechanical forces [e.g. extracellular matrix (ECM) stiffness, flow, stretch] control angiogenesis (Mammoto et al., 2009, 2022; Thodeti et al., 2009) and alveolar epithelial morphogenesis (Mammoto and Mammoto, 2019; Mammoto et al., 2013 b). We have reported that ECM stiffness controls postnatal lung vascular development (Mammoto et al., 2013b). Mechanical deformation/expansion of alveoli is also one of the major stimuli for surfactant secretion (Diem et al., 2020; Edwards, 2001). Paxillin is one of the focal adhesion adaptor LIM domain family proteins (Sero et al., 2012; Turner, 2000). Paxillin senses mechanical forces (Sero et al., 2011; Stutchbury et al., 2017) and plays an important role in tissue remodeling and embryonic development (Brown and Turner, 2004; Hagel et al., 2002; Noh et al., 2021; Plotnikov et al., 2012). We have previously reported that paxillin controls directional migration of endothelial cells (ECs) and fibroblasts (Sero et al., 2011) and regulates neonatal retinal vascular development (German et al., 2014). The role of AT2 cell paxillin in surfactant homeostasis and postnatal lung alveolar development remains unclear.

Here, we demonstrate that paxillin in AT2 cells is necessary for vascular and alveolar morphogenesis in the neonatal mouse lungs. Knockdown of paxillin in AT2 cells suppresses the levels of CEBPA to inhibit ABCA3 expression, which disrupts LB structures and attenuates postnatal lung alveolar development. Modulation of paxillin in AT2 cells could be an efficient strategy for neonatal lung developmental disorder.

## RESULTS

### Paxillin in AT2 cells is required for alveolar morphogenesis in neonatal mouse lungs

**Fig. 1.**
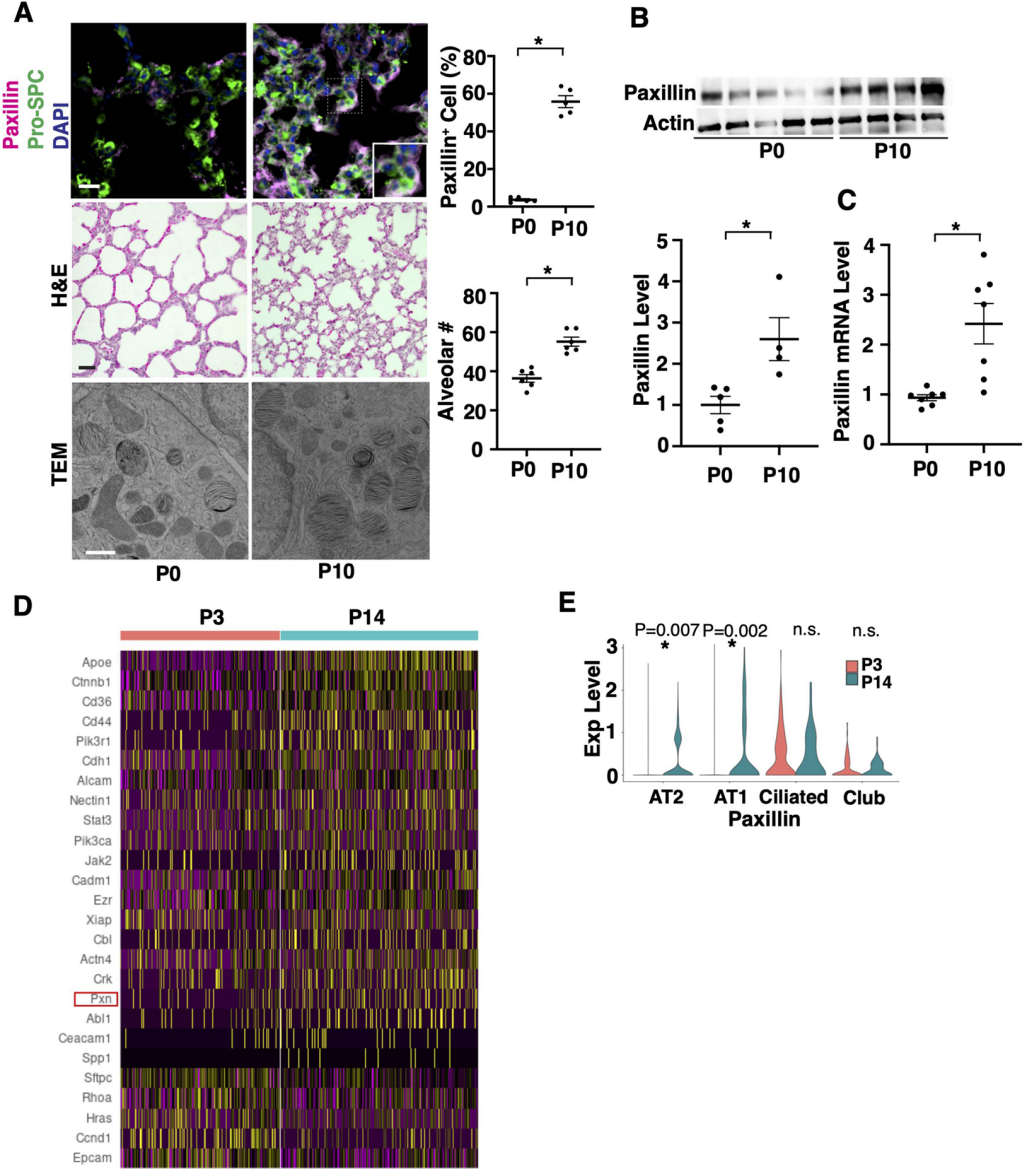
**Paxillin expression increases in alveolar epithelial cells during postnatal lung development.** (A) Immunofluorescence (IF) micrographs showing Pro-SPC-positive AT2 cells, paxillin expression and DAPI (top row insert: higher magnification image), H&E stained micrographs (middle row), and TEM images (bottom row) of P0 and P10 mouse lungs. Scale bars: IHC: 25 µm; HE: 50 µm; TEM: 800 nm. Quantification of % of paxillin expressing Pro-SPC-positive cells (top) and alveolar number (bottom) in the P0 and P10 mouse lungs (*n*=5-6, mean±s.e.m., *P<0.05). (B) Immunoblots showing paxillin and β-actin protein levels in EpCAM^+^ alveolar epithelial cells in the P0 and P10 mouse lungs (top). Quantification of paxillin protein levels normalized by actin (bottom, *n*=4-5, mean±s.e.m., *P<0.05). (C) Paxillin mRNA levels in EpCAM^+^ alveolar epithelial cells in the P0 and P10 mouse lungs (*n*=7, mean±s.e.m., *P<0.05). (D) Heatmap of focal adhesion/paxillin related genes in the P3 versus P14 mouse lung AT2 cell clusters. (E) Violin plots showing the expression of paxillin in the P3 and P14 mouse lung epithelial cell subpopulations. Data were analyzed by an unpaired Student's *t*-test.

Paxillin is necessary for tissue remodeling (Brown and Turner, 2004; Noh et al., 2021; Plotnikov et al., 2012) and embryonic development (Hagel et al., 2002). Expression of paxillin in AT2 cells is higher in the P10 CD1 mouse lungs (alveolar stage) compared to P0 pups (saccular stage), in which the alveolar number increased (Fig. 1A). Consistently, the mRNA and protein levels of paxillin in EpCAM^+^ alveolar epithelial cells isolated from P10 mouse lungs (Fig. S1A) measured using qRT-PCR and IB, respectively, were higher than those in the P0 mouse alveolar epithelial cells (Fig. 1B,C). We also analyzed publicly available scRNAseq data of P3 (saccular stage) versus P14 (alveolar stage) mouse lungs (GSE151974) (Hurskainen et al., 2021). Differential gene expression analysis of the AT2 cell cluster from P14 mouse lungs compared to those from P3 mouse lungs revealed that there are 879 significantly upregulated and 214 significantly downregulated genes that met the criteria of *P*-adjusted value <0.05 (Table S1); among them, 21 upregulated (e.g. Pxn, Cdh1, Alcam, Crk) and 5 downregulated genes (Epcam, Ccnd1, Rhoa, Sftpc, Hras) were related to focal adhesion remodeling (Fig. 1D). Paxillin was one of the significantly differentially expressed genes (P3 versus P14) in AT2 and AT1 cells (AT2: Log2FC, 0.136; *P*-adjusted value, 0.00714, AT1: Log2FC, 0.616; *P*-adjusted value, 0.002, Fig. 1E). The significantly differentially expressed genes in AT2 cells generated 583 BP GO Terms categories when analyzed using DAVID (Table S2). Of these 583 BP GO Terms, top 50 BP GO Terms include focal adhesion-related genes (e.g. Abl1, Actn4, Cdh1, Ctnnb1, Epcam, Pdlim2, Pxn) detected as appearing on the master list comprised of Gene Card (Fig. S2B, Table S2). These 50 BP GO terms include focal adhesion and development-related (e.g. actin cytoskeleton organization; positive regulation of cell migration; regulation of cell shape), cell cycle-related (e.g. apoptotic process, G1/S transition, cell proliferation), gene regulation and cell signaling-related (e.g. transcription, electron transport chain, translation), and lipid metabolism-related (e.g. lipid metabolic process, fatty acid metabolic process) BP GO Terms (Fig. S2B). IPA network analysis of all genes from top categories revealed the direct and indirect interaction with paxillin (Fig. S2C). These results suggest that paxillin is involved in postnatal lung alveolar formation by interacting with focal adhesion-, lipid metabolism-, and cell cycle-related genes.

**Fig. 2.**
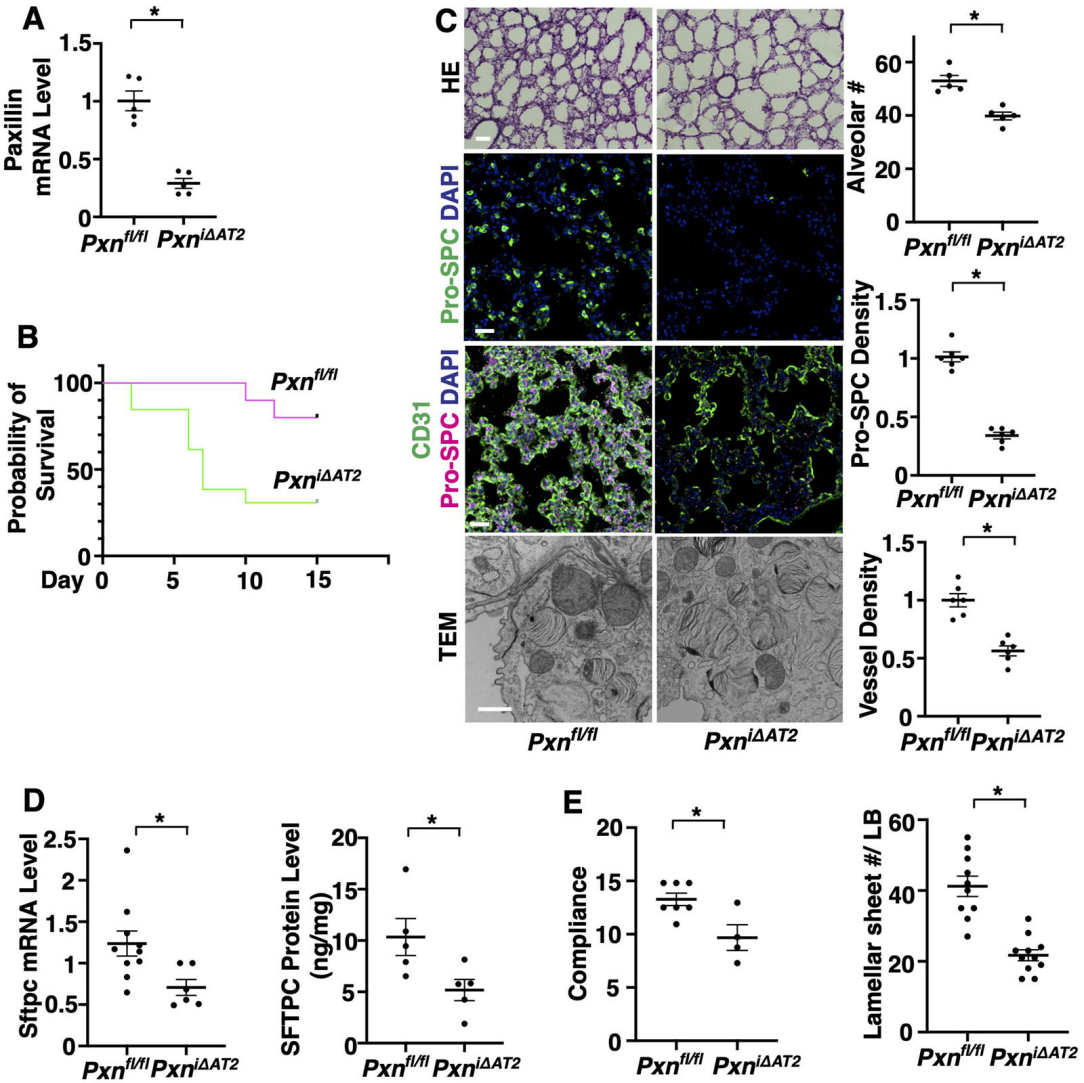
**AT2 cell paxillin is required for lung alveolar structure and function.** (A) Paxillin mRNA levels in EpCAM^+^ alveolar epithelial cells in the *Pxn^i^*^Δ*AT2*^ and *Pxn^fl/fl^* mouse lungs after tamoxifen induction (*n*=5, mean±s.e.m., *P<0.05). (B) Kaplan–Meier survival curves of *Pxn^fl/fl^* and *Pxn^i^*^Δ*AT2*^ mice after tamoxifen induction (*n*=10-13, *P*=0.0103 by log-rank test). (C) H&E-stained micrographs (top row), IF micrographs showing Pro-SPC-positive AT2 cells and DAPI (second row from top), CD31-positive blood vessels, Pro-SPC-positive AT2 cells and DAPI (third row from top) and TEM images (bottom row) of P10 *Pxn^i^*^Δ*AT2*^ and control *Pxn^fl/fl^* mouse lungs. Scale bars: HE and IHC: 50 µm; TEM: 800 nm. Quantification of alveolar number (top), Pro-SPC-positive cells (second row), blood vessel density (third row), and number of lamellar membrane sheets per LB (bottom row) in the P10 *Pxn^i^*^Δ*AT2*^ and control *Pxn^fl/fl^* mouse lungs (*n*=5-6, mean±s.e.m., *P<0.05). (D) Sftpc mRNA and protein levels in the P10 *Pxn^i^*^Δ*AT2*^ and *Pxn^fl/fl^* mouse lungs (*n*=5-10, mean±s.e.m., *P<0.05). (E) Static lung compliance in the P10 *Pxn^i^*^Δ*AT2*^ and *Pxn^fl/fl^* mouse lungs (*n*=4-7, mean±s.e.m., *P<0.05). Data were analyzed by an unpaired Student's *t*-test (A,C-E).

To directly examine the effects of AT2 cell paxillin on postnatal lung development, we developed *Pxn^i^*^Δ*AT2*^ mice by crossing *Pxn^fl/fl^* mice (obtained from Dr Christopher Turner, SUNY Upstate Medical University, USA) (Rashid et al., 2017) with *Sftpc-CreER^T2^* mice (Jackson Laboratory, 28054) (Kobayashi et al., 2020), in which cre recombination is induced in AT2 cells by tamoxifen induction (125 µg/mouse, five times from P0). Paxillin mRNA expression decreased by 73% in isolated lung EpCAM^+^ epithelial cells compared with those from control *Pxn^fl/fl^* mice (Fig. 2A). The survival rate was significantly lower in tamoxifen-induced *Pxn^i^*^Δ*AT2*^ neonatal mice (P15, Fig. 2B); at P10, only 10% of the *Pxn^fl/fl^* neonatal pups died, whereas 70% of tamoxifen-induced *Pxn^i^*^Δ*AT2*^ mice died. The number of alveoli and the density of Pro-SPC^+^ AT2 cells were 24% and 68% lower, respectively, in tamoxifen-induced *Pxn^i^*^Δ*AT2*^ mouse lungs compared to control *Pxn^fl/fl^* mice (Fig. 2C). Vascular formation was also attenuated; the CD31^+^ blood vessel density was 48% lower in P10 *Pxn^i^*^Δ*AT2*^ mouse lungs (Fig. 2C). Lamellar structure of LBs, in which surfactant lipids are packaged and stored (Dietl et al., 2001; Frick et al., 2001; Perez-Gil and Weaver, 2010; Weaver et al., 2002), was disrupted in tamoxifen-induced *Pxn^i^*^Δ*AT2*^ mice; the number of the LBs per cell was not significantly different between tamoxifen-induced P10 *Pxn^i^*^Δ*AT2*^ mouse lung AT2 cells and control *Pxn^fl/fl^* AT2 cells, however the number of lamellar membrane sheets per LB was significantly lower in tamoxifen-induced P10 *Pxn^i^*^Δ*AT2*^ mouse lungs than in control *Pxn^fl/fl^* mice (Fig. 2C). Consistently, the mRNA and protein levels of Sftpc were lower in EpCAM^+^ alveolar epithelial cells isolated from *Pxn^i^*^Δ*AT2*^ mouse lungs compared to control *Pxn^fl/fl^* mice (Fig. 2D) and lung compliance was significantly lower in *Pxn^i^*^Δ*AT2*^ mouse lungs (Fig. 2E).

### AT2 paxillin controls ABCA3 expression through CEBPA during postnatal lung development

**Fig. 3.**
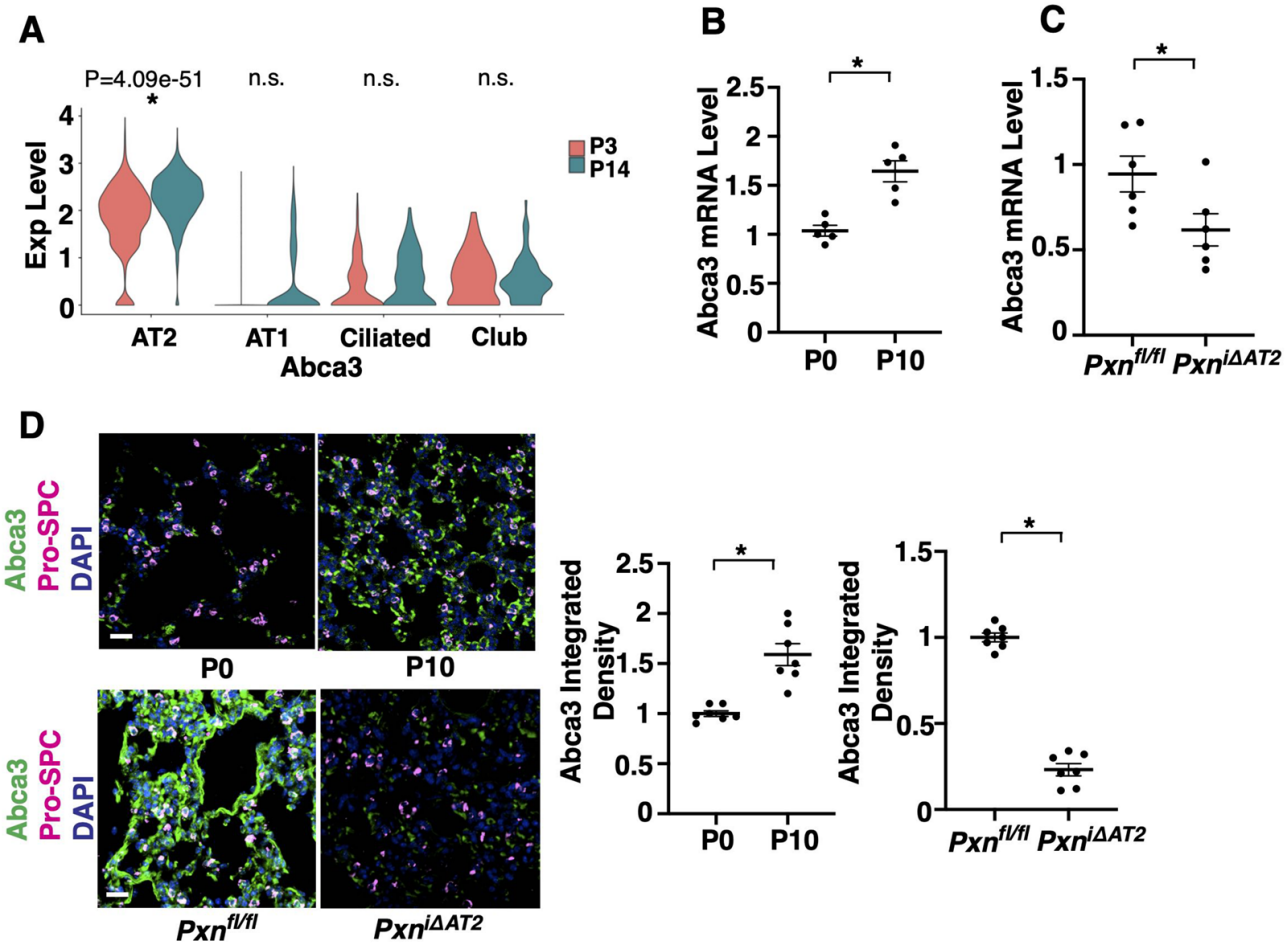
**Abca3 expression is inhibited in the *Pxn^i^*^Δ*AT2*^ mouse lung AT2 cells.** (A) Violin plots showing the expression of Abca3 in the P3 and P14 mouse lung epithelial cell subpopulations. (B) Abca3 mRNA levels in EpCAM^+^ alveolar epithelial cells in the P0 and P10 mouse lungs (*n*=5, mean±s.e.m., *P<0.05). (C) Abca3 mRNA levels in EpCAM^+^ alveolar epithelial cells in the P10 *Pxn^i^*^Δ*AT2*^ and *Pxn^fl/fl^* mouse lungs (*n*=6, mean±s.e.m., *P<0.05). (D) RNA-ISH and IF images showing Abca3 expression, Pro-SPC^+^ AT2 cells and DAPI in the P0 and P10 mouse lungs (top row) and P10 *Pxn^i^*^Δ*AT2*^ and *Pxn^fl/fl^* mouse lungs (bottom row). Scale bars: 50 µm. Quantification of Abca3 expression in the P0 and P10 mouse lungs (*n*=7, mean±s.e.m., *P<0.05) and the P10 *Pxn^i^*^Δ*AT2*^ and *Pxn^fl/fl^* mouse lungs (*n*=7, mean±s.e.m., *P<0.05). Data were analyzed by an unpaired Student's *t*-test.

The ABC transporters translocate surfactant lipids across cell membranes (Baldan et al., 2009; Takahashi et al., 2005; Wenzel et al., 2007). In the lung, ABCA3 transports surfactant lipids into LBs and controls LB formation and surfactant homeostasis during lung development (Ban et al., 2007; Mulugeta et al., 2002; Rindler et al., 2017). In the publicly available scRNAseq data of P3 and P14 mouse lungs (GSE151974) (Hurskainen et al., 2021), Abca3 expression was significantly increased in P14 (alveolar stage) AT2 cells compared to P3 saccular stage (Log2FC, 0.478; *P*-adjusted value, 4.09e-51; Fig. 3A). Consistently, the mRNA levels of Abca3 in EpCAM^+^ alveolar epithelial cells (Fig. 3B, qRT-PCR) and in the lungs (Fig. 3D, RNA-ISH) were 1.7- and 1.6-times higher, respectively, in the P10 mouse lungs compared to those in P0 lungs. Knockdown of paxillin in AT2 cells suppressed Abca3 expression in the tamoxifen-induced P10 *Pxn^i^*^Δ*AT2*^ mouse lungs (Fig. 3C,D), suggesting that paxillin in AT2 cells mediates alveolar morphogenesis and function by maintaining LB structure through ABCA3 signaling during postnatal lung development.

**Fig. 4.**
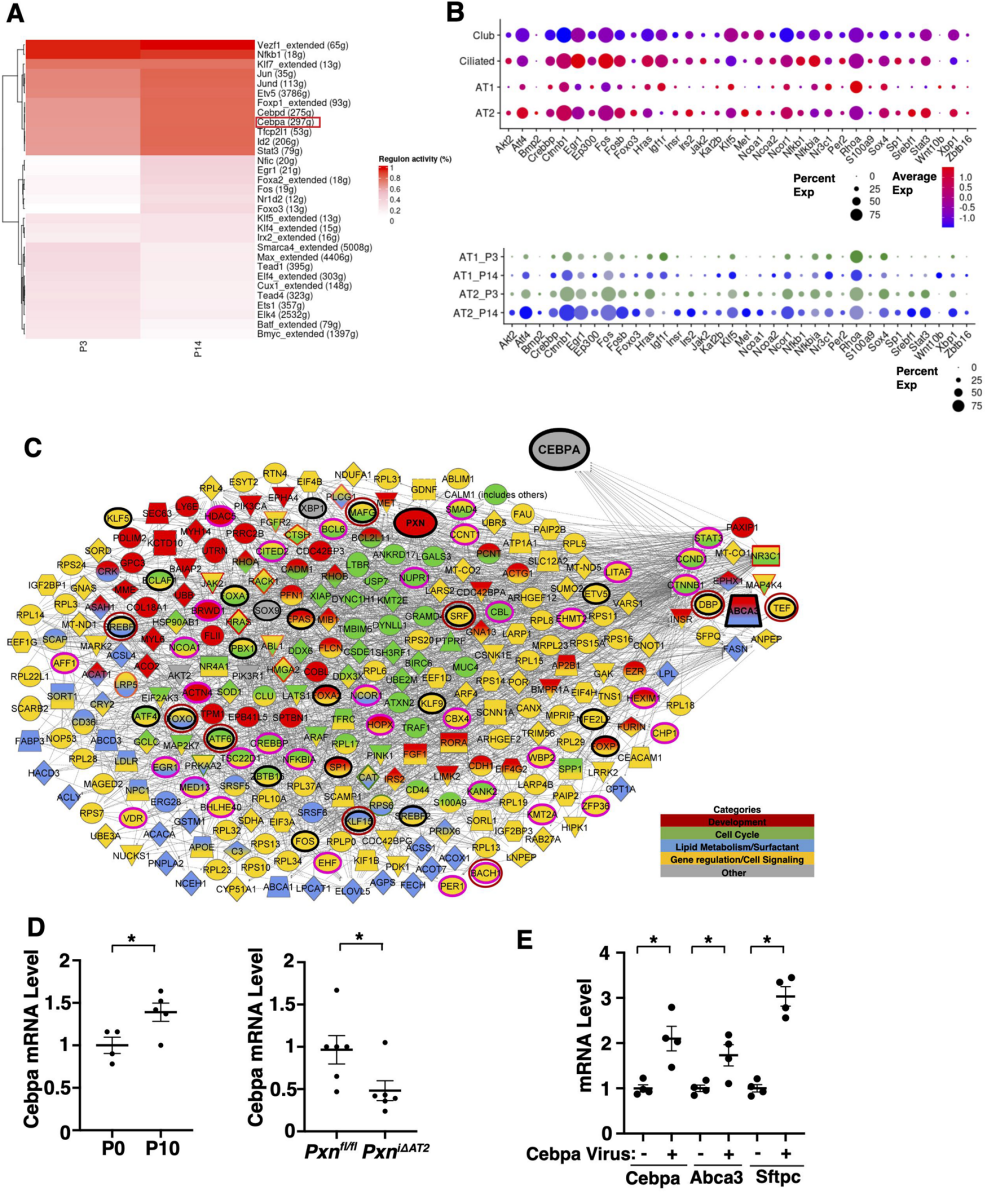
**Cebpa mediates Abca3 expression in mouse lung AT2 cells.** (A) Heatmap of top 25% regulons from AT1 and AT2 cells in the P3 and P14 mouse lungs. (B) Dot plots showing differentially expressed Cebpa-related genes identified in the P14 mouse lung epithelial cell subpopulations (top) and P3 and P14 in AT1 and AT2 cells (bottom). (C) IPA network analysis of direct or indirect interactions between Cebpa and significantly differentially expressed genes in the top 50 BPGO Terms from AT2 cluster of the P3 versus P14 mouse lungs. Pink bold: transcription regulators; black bold: transcription factors; red bold: regulons with at least 25% active genes. (D) Cebpa mRNA levels in the P0 and P10 mouse lung AT2 cells (*left*, *n*=4-5, mean±s.e.m., *P<0.05). Cebpa mRNA levels in the P10 *Pxn^i^*^Δ*AT2*^ and *Pxn^fl/fl^* mouse lung AT2 cells (*right*, *n*=6, mean±s.e.m., *P<0.05). (E) mRNA levels of Cebpa, Abca3 and Sftpc in the P10 mouse lung EpCAM^+^ cells treated with retrovirus overexpressing Cebpa (*n*=4, mean±s.e.m., *P<0.05). Data were analyzed by an unpaired Student's *t*-test.

A transcription factor CEBPA controls ABCA3 expression to regulate tissue development and lipid metabolism (Cao et al., 1991; Sugahara et al., 2001). Knockdown of Cebpa in AT2 cells inhibits fetal lung development (Martis et al., 2006). When we analyzed the transcriptional gene regulatory network in AT1/AT2 cells using SCENIC, Cebpa was in the list of AT1/AT2 REGULONS with at least 25% active genes, and its network activity was higher in P14 mouse lungs compared to that in P3 mouse lungs (Fig. 4A, GSE151974; Hurskainen et al., 2021). Cebpa-related genes (e.g. Ctnnb1, Fos, Egr1, Ncor1, Stat3, Klf5) were highly expressed in AT2 cells compared to other alveolar epithelial cell clusters, which was significantly differentially expressed in P14 mouse lung AT2 cells compared to P3 (Fig. 4B). IPA network analysis revealed that Cebpa directly (15 genes, e.g. Abca3, Tef, Stat3) or indirectly (271 genes, e.g. Pxn, Foxo, Smad4, Klf5, Epas) interacted with differentially expressed genes related to lipid metabolism and organ development in AT2 cells (Fig. 4C). These genes include 34 transcription regulators (e.g. BCL6, CBL, CCNT1, CTNNB1, PER1, NFKBIA, SMAD4; pink bold outline), 25 transcription factors (e.g. ATF4, EPAS1, ETV5, SOX9, FOS, KLF5; black bold outline), and 8 genes from SCENIC analysis of AT1/AT2 cells in the P3 versus P14 mouse lungs (MAFG, TEF, DBP, SREBF1, KLF15, FOXO3, ATF6, SRF; black and red outline, Fig. 4C), suggesting that Cebpa interacts with paxillin, Abca3, and other organ development and surfactant metabolism related genes to control lung development.

Consistent with fetal lungs (Li et al., 1995), the mRNA levels of Cebpa were higher in EpCAM^+^ alveolar epithelial cells isolated from P10 mouse lungs compared to those in P0 mouse lungs (Fig. 4D). The levels of Cebpa were suppressed in the tamoxifen-induced P10 *Pxn^i^*^Δ*AT2*^ mouse lung EpCAM^+^ alveolar epithelial cells (Fig. 4D). Overexpression of Cebpa using retroviral transduction stimulated Abca3 and Sftpc expression in P10 mouse lung EpCAM^+^ alveolar epithelial cells (Fig. 4E).

**Fig. 5.**
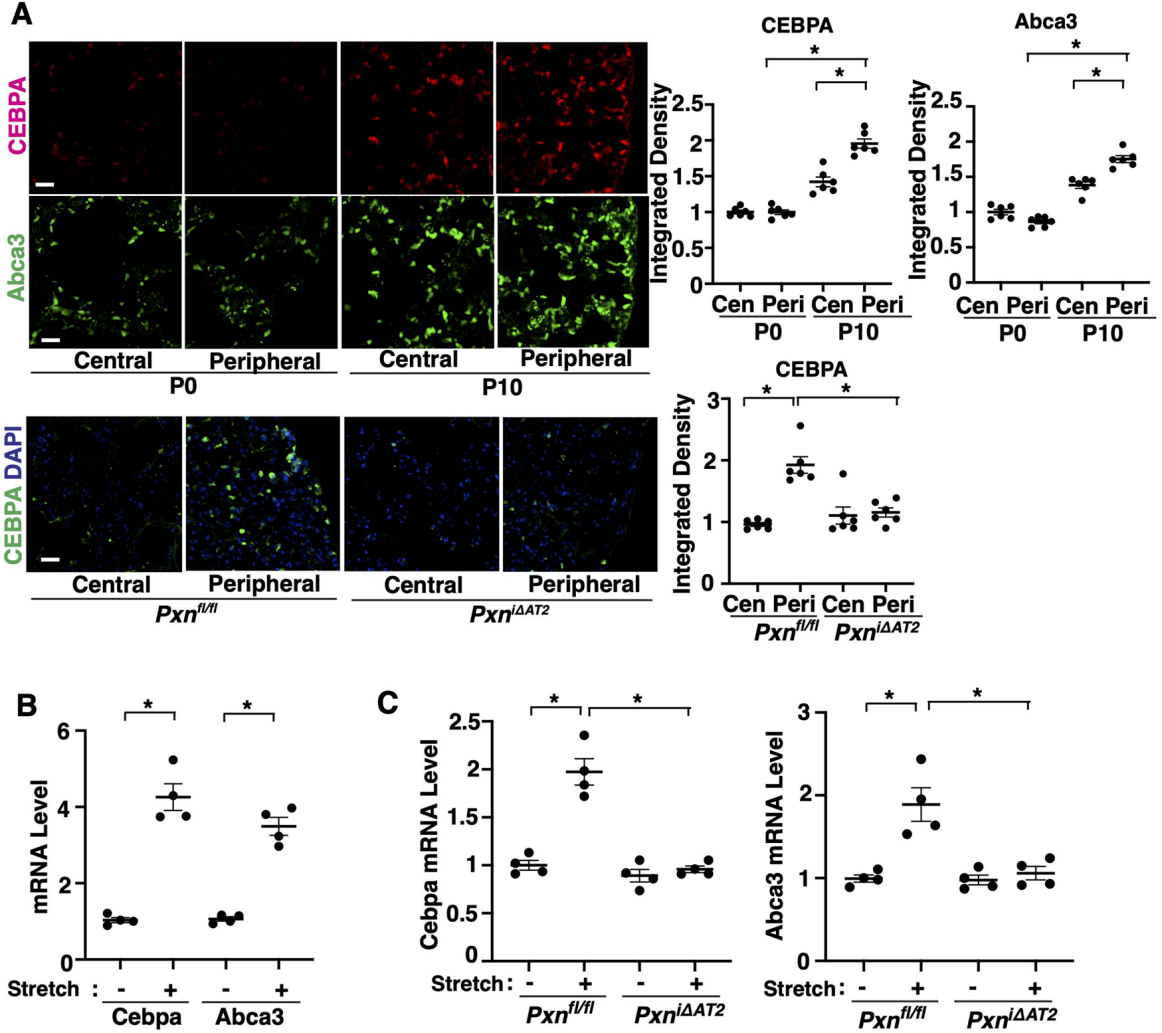
**Cebpa and Abca3 expression is stimulated in the peripheral region of postnatal mouse lungs.** (A) RNA-ISH and IF micrographs showing CEBPA expression (top) and Abca3 expression (middle) in the central and peripheral regions of P0 and P10 mouse lungs. IF micrographs showing CEBPA expression and DAPI in the central and peripheral regions of P10 *Pxn^i^*^Δ*AT2*^ and control *Pxn^fl/fl^* mouse lungs (bottom). Scale bars: 50 µm. Quantification of CEBPA and Abca3 expression in the P0 and P10 mouse and P10 *Pxn^i^*^Δ*AT2*^ and control *Pxn^fl/fl^* mouse lungs (*n*=6, mean±s.e.m., *P<0.05). (B) mRNA levels of Cebpa and Abca3 in the P10 mouse lung EpCAM^+^ cells with or without stretching (*n*=4, mean±s.e.m., *P<0.05). (C) mRNA levels of Cebpa and Abca3 in the P10 *Pxn^i^*^Δ*AT2*^ and *Pxn^fl/fl^* mouse lung EpCAM^+^ cells with or without stretching (*n*=4, mean±s.e.m., *P<0.05). Data were analyzed by one-way ANOVA with Bonferroni test (A,C) or an unpaired Student's *t*-test (B).

It has been reported that CEBP is highly expressed in the peripheral region of fetal lungs (Li et al., 1995), where mechanical forces are significantly altered during tissue regeneration (Filipovic et al., 2013; Yilmaz et al., 2009, 2011). Consistently, the levels of CEBPA were significantly higher at the peripheral region of P10 mouse lungs compared to those in P0 mouse lungs and at the central region; the effects were suppressed in the tamoxifen-induced P10 *Pxn^i^*^Δ*AT2*^ mouse lungs (Fig. 5A). Similarly, the levels of Abca3 were significantly higher at the peripheral region of P10 mouse lungs (Fig. 5A). Since paxillin senses mechanical forces and controls cell migration and angiogenesis (German et al., 2014; Sero et al., 2012, 2011), we also examined the effects of mechanical stretching on Cebpa and Abca3 expression. When we applied uniaxial cyclic stretch (10%) to EpCAM^+^ alveolar epithelial cells isolated from mouse lungs adherent to flexible ECM substrates for 16 h as we reported (Thodeti et al., 2009), 10% uniaxial cyclic stretch of alveolar epithelial cells increased the mRNA levels of Cebpa and Abca3 (Fig. 5B). Knockdown of paxillin inhibited the mRNA levels of Cebpa and Abca3 induced by stretching (Fig. 5C). These results indicate that paxillin mediates postnatal lung development through CEBPA-ABCA3 signaling in AT2 cells.

## DISCUSSION

Here we have demonstrated that paxillin in alveolar epithelial cells controls lung alveolar morphogenesis through CEBPA-ABCA3 signaling. The levels of paxillin increase during neonatal development, while knockdown of paxillin in AT2 cells decreases expression of CEBPA to inhibit ABCA3 expression that controls surfactant homeostasis, inhibiting alveolar development in the mouse lungs. Modulation of paxillin in AT2 cells may be novel interventions for neonatal lung developmental disorder.

In addition to chemical signaling, the micromechanical environment controls vascular and epithelial morphogenesis and function (Mammoto and Mammoto, 2019; Mammoto et al., 2012a). We have reported that ECM stiffness controls angiogenesis and vascular integrity (Mammoto et al., 2009, 2013a); hydrostatic pressure increased after pneumonectomy stimulates angiogenic factor expression and vascular formation in the mouse lungs (Mammoto et al., 2022); and age-dependent changes in EC size mediate age-dependent decline in angiogenesis (Mammoto et al., 2019b). We have also demonstrated that ECM stiffness controls vascular and alveolar development in the neonatal mouse lungs (Mammoto et al., 2013b). Other mechanical forces such as shear flow and stretching forces are also altered during postnatal lung development (Fang et al., 2019; Mammoto and Mammoto, 2019). Paxillin senses mechanical forces and controls cell behaviors (Sero et al., 2012, 2011). In fact, (1) scRNAseq analyses of postnatal lungs (saccular stage versus alveolar stage) reveal that focal adhesion- and actin cytoskeleton-related genes are significantly differentially expressed and interacted with paxillin (Fig. 1D, Table S2, Fig. S2B,C); (2) CEBPA and ABCA3 expression is higher at the peripheral region of P10 mouse lungs (Li et al., 1995), where mechanical forces and tissue deformation are dynamically changed (Filipovic et al., 2013; Yilmaz et al., 2009, 2011) and cell proliferation and gene expression are upregulated (Mammoto et al., 2022) (Fig. 5A); and (3) stretching forces increase CEBPA and ABCA3 expression in alveolar epithelial cells, while knockdown of paxillin inhibited the effects (Fig. 5B,C). Peripheral region of the lungs has special chemical and mechanical characteristics; (1) less septal tissues (Zeltner et al., 1990) (2) involvement of systemic (bronchial) and pulmonary vessels (Marchand et al., 1950), and (3) abundant stem cells (Herrick and Mutsaers, 2004), which changes cell behaviors in a spatiotemporal manner. It is also reported that differentiation of AT2 cells to AT1 cells is more prominent at the peripheral region during repair from lung injury (Kobayashi et al., 2020). Thus, paxillin in AT2 cells may sense spatial difference in the chemical and mechanical stimuli altered during lung development and control alveolar morphogenesis by changing expression of CEBPA and ABCA3.

In addition to surfactant signaling, stretching forces increase AT1 marker gene Rage expression (Burgess et al., 2024) in P10 mouse EpCAM+ alveolar epithelial cells, which is inhibited by paxillin knockdown (Fig. S1C). These results suggest that stretching forces on EpCAM+ alveolar epithelial cells may control alveolar morphogenesis by stimulating surfactant homeostasis through increasing Cebpa expression as well as promoting AT1 progenitor program. This is consistent with reports suggesting that (1) mechanical forces contribute to alveolar fate acquisition (Foster et al., 2010; Li et al., 2018); (2) alveolar progenitors that differentiate into AT2 and AT1 cells acquire AT1 fate through a secondary signal provided by nascent AT2 cells, which is enhanced by mechanical forces (Brownfield et al., 2022). However, it has been reported that Cebpa deletion in neonatal AT2 cells leads to reversion to the progenitor program as well as to an increase of AT1-like cells (Hassan and Chen, 2024). Stretching dependent changes in the expression of other progenitor reprogramming factors (e.g. FGFR2 ligands; Brownfield et al., 2022) may also be involved in the reprogramming program of AT1 and AT2 cells during alveolar morphogenesis. Alternatively, spatiotemporal changes in the different types of mechanical forces during perinatal stages may differentially alter the expression and response of Cebpa and other soluble factors to mechanical forces as well as the way how they control the progenitor reprogramming program.

We have demonstrated that paxillin knockdown in AT2 cells decreases CEBPA and ABCA3 expression and inhibits lung vascular and alveolar morphogenesis, suppressing lung compliance and postnatal survival (Figs 2-4). Although CEBPA overexpression increased the levels of Abca3 and Sftpc in alveolar epithelial cells (Fig. 4E), CEBPA overexpression was not able to restore the expression of Abca3 and Sftpc suppressed by paxillin knockdown (not shown). Paxillin may control ABCA3 expression and surfactant homeostasis through other signaling pathways as well. Paxillin is also involved in inflammatory response and contributes to lung injury (Gawlak et al., 2014; Kelly et al., 2019). It has been reported that knockdown of ABCA3 in AT2 cells increases expression of genes associated with organ injury and inflammation, which stimulates capillary leak and inhibits surfactant secretion and activity (Berry et al., 1986), leading to alveolar damage (Rindler et al., 2017). Thus, paxillin may also sense chemical signaling due to inflammation and associated changes in mechanical forces, and there is a complex feedback mechanism contributing to inhibition of lung alveolar morphogenesis in *Pxn^i^*^Δ*AT2*^ mouse lungs. The effects of paxillin knockdown in AT2 cells on suppression of alveolar morphogenesis may be the secondary and indirectly consequence (e.g. migration, actin cytoskeleton remodeling) not solely due to its effects on surfactant homeostasis and reprogramming of progenitors given the multiple functions of paxillin. However, we have reported that cell adhesion of paxillin knocked down (siRNA) ECs is not significantly altered compared to those treated with control scrambled siRNA (German et al., 2014). Consistently, cell adhesion of paxillin knocked down (siRNA) EpCAM^+^ alveolar epithelial cells is not significantly inhibited (not shown), which may be because of the effects of other focal adhesion proteins. Thus, paxillin may control alveolar morphogenesis at least partly through inhibiting surfactant homeostasis via CEBPA-ABCA3 signaling. Further investigation of the effects of other focal adhesion proteins on alveolar development will elucidate the mechanism.

Here, we have demonstrated that paxillin in AT2 cells controls postnatal lung alveolar morphogenesis through CEBPA-ABCA3 signaling. It remains unknown the relationship between paxillin and CEBPA signaling. It has been reported that paxillin controls Rho/Rac activity (Xu et al., 2022), which stimulates mechanotransduction pathways (e.g. YAP/TAZ, Wnt signaling; Esposito et al., 2022; Meng et al., 2016; Zhan et al., 2017). Wnt-βcatenin signaling controls CEBPA expression and transcriptional activity in liver cancer cells (Nakagawa et al., 2024). We have demonstrated that ECM stiffness controls postnatal lung development through LRP5 signaling (Mammoto et al., 2013b) and LRP5 is one of the significantly differentially expressed genes in the scRNAseq analysis of P3 versus P14 mouse lung AT2 cells that interact with CEBPA/ABCA3 and paxillin (Fig. 4C). It has been reported that paxillin also localizes in the nucleus to directly exert its transcription activity in cancer cells (Noh et al., 2021; Sen et al., 2012). However, our IHC results suggest that paxillin expresses in the cytoplasm of AT2 and AT1 cells during perinatal development (Fig. 1A, Fig. S1B). Paxillin may directly or indirectly interact with transcription factors, co-factors, or regulators (e.g. YAP, β-catenin etc.) in the cytoplasm and control nuclear localization and transcriptional activity of these genes to regulate CEBPA-ABCA3 transcription and expression during AT2 cell maturation. Thus, paxillin and its effects on the actin cytoskeleton may impact CEBPA activity by modulating mechanosensitive signaling pathways. We show that LB structures are disrupted in *Pxn^i^*^Δ*AT2*^ mouse lungs (Fig. 2C). Knockdown of paxillin may disrupt LB structures by suppressing expression of lipid transporter ABCA3 that is critical for LB biogenesis and surfactant protein processing during lung development (Cheong et al., 2007; Dietl and Frick, 2021). In fact, AT2 cells from Abca3^−/−^ embryos do not contain LBs and expression of mature SPB protein was suppressed (Cheong et al., 2007). LB biogenesis and SPB processing are also attenuated in human patients with ABCA3 mutations (Brasch et al., 2006; Bullard et al., 2005). Since paxillin is a cytoskeletal/focal adhesion molecule, knockdown of paxillin may also directly suppress LB extrusion and consequently inhibit surfactant secretion and attenuate lung compliance (Fig. 2E). It has been reported that actin contraction controlled by selective recruitment of actin cytoskeleton modulating molecules (e.g. Rho, Rock, MLCK) stimulates LB compression, extrusion, and surfactant secretion in AT2 cells (Miklavc et al., 2015). In the scRNAseq analysis, although Rhoa expression was significantly lower in AT2 cells at P14 compared to P3, the levels of Rho activators, Arhgef12 and Arhgef38 were significantly higher in P14 AT2 cells (Fig. 1D, Table S1). Paxillin may control Rho and Rac activity to regulate actin cytoskeleton remodeling (Chen et al., 2005; Deakin and Turner, 2008) and stimulate LB structure and surfactant secretion. In this study, TEM is used for analysis of LB structures. Using cryo-EM will further enable us to visualize and understand the formation of the three-dimensional structure of the membrane stacks (Klein et al., 2021).

Epithelial/endothelial–mesenchymal transition (EMT/EndMT) plays key roles in organ development and contributes to various lung pathologies such as chronic obstructive pulmonary disease (COPD), lung cancer, and pulmonary fibrosis (Jolly et al., 2018; Rout-Pitt et al., 2018). It has been reported that paxillin knockdown suppresses EMT during tumor progression and metastasis (Wen et al., 2020). Thus, paxillin signaling may control lung vascular and alveolar development through EMT/EndMT as well. In fact, EMT related genes (e.g. SMAD4, RGCC, LRG1, BAMBI, CTNNB1, AGER, BCL9 L, DDX17, EPB41L5, HMGA2, SOX9, FGFR2) are up- and down regulated in the scRNAseq analysis of P3 versus P14 mouse lung AT2 cells (Table S1). Among them, SMAD4, CTNNB1, HMGA2, SOX9 interacted with CEBPA and paxillin (Fig. 4C), suggesting that paxillin may control lung alveolar development through EMT signaling as well.

In summary, we have demonstrated that paxillin in AT2 cells controls alveolar development through CEBPA-ABCA3 signaling. Modulation of AT2 cell paxillin may accelerate lung development in premature babies and potentially lead to the development of new therapeutic strategies for lung developmental disorders.

## MATERIALS AND METHODS

### Materials

Anti-paxillin (610619; 1:100 for IHC, 1:500 for IB) and -CD31 (553370; 1:100 for IHC) antibodies were from BD Biosciences (San Jose, CA, USA). Anti-CEBPA (8178; 1:100 for IHC) antibody was from Cell Signaling (Danvers, MA, USA). Anti-pro SPC (AB3786; 1:100 for IHC) and -β-actin (A5441; 1:1000 for IB) antibodies were from Sigma (St. Louis, MO, USA). Anti-RAGE (MAB1179; 1:100 for IHC) antibody was from R&D systems (Minneapolis, MN, USA).

### Molecular biological and biochemical methods

The retroviral WZLneo-C/EBPa was a gift from Kai Ge (Addgene Plasmid, 34567). Generation of viral vectors was accomplished as reported (Mammoto et al., 2009, 2019a). Mouse alveolar epithelial cells were incubated with viral stocks in the presence of 5 μg/ml polybrene (Sigma) and 90-100% infection was achieved 3 days later (Mammoto et al., 2009, 2019a). Quantitative reverse transcription (qRT)-PCR was performed with the iScript reverse transcription and iTaq SYBR Green qPCR kit (Bio-Rad, Hercules, CA, USA) using the Bio-Rad real time PCR system (Bio-Rad). Cyclophilin controlled for overall cDNA content. The primers used for mouse cyclophilin were previously described (Mammoto et al., 2009, 2019a). The primers used for mouse Abca3 forward; CAGCTCACCCTCCTACTCTG, reverse; ACTGGATCTTCAAGCGAAGCC, mouse Sftpc forward; ATGGACATGAGTAGCAAAGAGGT, reverse; CACGATGAGAAGGCGTTTGAG, mouse paxillin forward; GGCATCCCAGAAAATAACACTCC, reverse; GCCCTGCATCTTGAAATCTGA, mouse Cebpa forward; CAAGAACAGCAACGAGTACCG, reverse; GTCACTGGTCAACTCCAGCAC, mouse Rage forward; CCACTGGAATTGTCGATGAGG, reverse; CTCGGACTCGGTAGTTGGACT. Protein levels of mouse SFTPC were measured using ELISA kit from MyBioSource (San Diego, CA, USA).

### Mouse lung tissue preparation

The *in vivo* animal study was carried out in strict accordance with the recommendations in the Guide for the Care and Use of Laboratory Animals of the National Institutes of Health. The protocol was reviewed and approved by the Animal Care and Use Committee of Medical College of Wisconsin (AUA5598). CD1 mice (Charles River Laboratory), *Pxn^fl/fl^*, and *Pxn^fl/fl^-Sftpc-CreER^T2^* (*Pxn^i^*^Δ*AT2*^) mice were used for the study. *Pxn^fl/fl^* mice (obtained from Dr Christopher Turner, SUNY Upstate Medical University) (Rashid et al., 2017) were crossed with *Sftpc-CreER^T2^* mice (Jackson Laboratory, 28054) (Kobayashi et al., 2020), an inducible cre deleter under the control of *Sftpc* promoter, to create mice with *Sftpc-Cre*-dependent conditional deletion of paxillin (*Pxn^i^*^Δ*AT2*^), in which cre recombination is induced in AT2 cells by administration of tamoxifen (125 µg/mouse, five times from P0). Mouse lung epithelial cells were isolated from mouse lungs using anti-EpCAM conjugated magnetic beads (Fig. S1A) (Chen and Liu, 2021; Mammoto et al., 2020, 2018). Isolated epithelial cells were validated by FACS and ICC for epithelial markers before use. Mouse lung compliance was measured as described previously (Mammoto et al., 2016a).

Histological samples were prepared and morphological analysis of vascular and alveolar structures was performed as previously described (Kyi et al., 2022; Mammoto et al., 2012b, 2016a,b, 2018, 2019a). Fluorescent images were taken on a Nikon A1 confocal laser scanning microscope and morphometric analysis was performed using ImageJ software as we reported (Kyi et al., 2022; Mammoto et al., 2012b, 2016a,b, 2018, 2019a).

### Mechanical strain application

Mouse lung epithelial cells (1×10^5^) were seeded on the stretch chamber made of poly-dimethylsiloxane (Strex Corp., Osaka, Japan) coated with collagen 24 h before stretching. The chambers were then applied to uniaxial cyclic strain (0.5 Hz, 10%) for 16 h (Thodeti et al., 2009). After stretching, cells were collected for subsequent analysis.

### Transmission electron microscopy (TEM)

Mouse lungs were collected and fixed using the method of Hirsch and Fedorko (Hirsch and Fedorko, 1968) to enhance LBs in AT2 cells. Essentially, freshly excised mouse lung pieces were immersed in a mixture 1-part 2.5% glut in 0.1 M cacodylate+2-parts OsO_4_ in 0.1 M sodium cacodylate buffer – mixed just before use - for 45 min on ice. Tissues were then washed with distilled water three times for 10 min each time and immersed in 0.25% aqueous uranyl acetate *en bloc* for 2 h (Locke et al., 1971). Tissues were again washed three times for 10 min each time with distilled water then dehydrated through graded methanol, acetonitrile washes and infiltrated with EMBed 812 epoxy resin (14120; Electron Microscopy Sciences, Hatfield, PA, USA). Ultrathin sections (70-80 nm) were cut with an Ultra 45° diamond knife (DiATOME, Hatfield, PA, USA), collected on to copper grids (G200H-Cu; Electron Microscopy Sciences) and stained with uranyl acetate and lead citrate for contrast. Images were captured using a JEOL 1400 Flash transmission electron microscope (JEOL, Tokyo, Japan) equipped with a 2 K×2 K ultrahigh resolution digital camera. The lamellar structures of LBs were quantified by measuring the number of lamellar membrane sheets per LB.

### RNA *in situ* hybridization (RNA-ISH)

RNAscope technology [Advanced Cell Diagnostics (ACD), Newark, CA, USA] was used for RNA-ISH. The formalin-fixed paraffin-embedded (FFPE) mouse lung tissue samples were sectioned at 5 μm thick using a microtome, mounted on slides, baked for 1 h at 60°C, then deparaffinized in xylene and 100% ethanol. For target retrieval, hydrogen peroxide (ACD 322381) was applied for 10 min at room temperature, followed by a mild boil at 98-102°C for 15 min in 1× target retrieval reagent buffer (ACD 322001). Sections were then treated with Protease Plus (ACD 322381) at 40°C for 30 min in hybridization oven. *In situ* detection of Abca3 was performed by hybridization with target probes [Mm-Abca3-01-C1 (ACD 1080651-C1)] following the ACD instruction with RNAscope Multiplex Fluorescent Reagent Kit v2. For dual ISH-immunohistochemistry (IHC), FFPE sections were first subjected to ISH as described above, followed by IHC such as blocking (1% fetal bovine serum in TBS) and incubation with primary and secondary antibodies. Images were taken on a Nikon A1 confocal laser scanning microscope and morphometric analysis was performed using ImageJ software as we reported (Kyi et al., 2022; Mammoto et al., 2012b, 2016a,b, 2018, 2019a).

### scRNA sequencing data analysis

Publicly available scRNA-seq dataset of P3 and P14 mouse lungs (GSE151974, normoxia) was used to analyze gene expression profiles of neonatal mouse lung alveolar epithelial cells (Hurskainen et al., 2021). All main processing steps, including quality control, integration, and clustering were performed as originally described (Hurskainen et al., 2021). Briefly, each demultiplexed unique sample was split into separate Seurat objects and SCTransform was used to normalize each sample and select highly variable genes. FindAllMarkers functions in Seurat were used to identify major cell type subsets. Epithelial cell populations (AT1, AT2, Ciliated, and Club) (Fig. S2A) were extracted from the dataset and subsequent analysis was presented in the form of dot plots, heat maps, and violin plots.

Differentially expressed genes were identified comparing AT2 clusters from P3 versus P14 mouse lungs using model-based analysis of single-cell transcriptomics (MAST) (Table S1). BP GO analysis of these genes with *P*-adjusted value of <0.05 was performed via the DAVID which generated 583 BP GO Terms categories (Table S2). The top 50 BP GO Terms were color-coded according to the categories such as development, cell cycle, lipid metabolism/surfactant, and gene regulation/cell signaling (Fig. S2B). Network generation was performed on all genes from top 50 BP GO Terms categories in P3 versus P14 mouse lung sorted by *P*-value using IPA software, and interaction with paxillin was analyzed. Genes connecting to less than three other genes were trimmed from the network. Another network was generated using the genes from the top 50 BP GO Terms of AT2 subpopulation, illustrating the direct and indirect interactions with Cebpa. Genes with direct interactions are the genes that have a direct physical connection to another gene and genes with indirect interactions are genes that have a relationship with another gene through one or more intermediary genes.

SCENIC package (version 1.1.2) was used to analyze the gene regulatory network of AT1 and AT2 cells in P3 versus P14 of mouse lungs using the Cis Target database in R. The binary regulon activity heatmap showed the regulons with absolute correlation >0.3 and active in at least 25% of AT1 and AT2 cells. Cebpa related genes were extracted from significantly differentially expressed genes with *P*-adjusted value of <0.05 from AT2 or other epithelial subpopulations and the expression levels of these genes were presented in dot plots.

### Statistical analysis

All phenotypic analysis was performed by masked observers unaware of the identity of experimental groups. Power analysis was performed to ensure that the number of animals provides a minimum of 80% power to detect treatment effect sizes of 20-30%. Data analyses were performed using GraphPad Prism 8.0. Error bars (s.e.m.) and *P*-values were determined from the results of three or more independent experiments. Student's *t*-test was used for statistical significance for two groups. For more than two groups, one-way ANOVA with a post-hoc using the Bonferroni test was conducted. Mouse survival was compared using the Kaplan–Meier log-rank test. *P*<0.05 was considered significant.

## Supplementary Material

10.1242/biolopen.061939_sup1 Supplementary information
