# Supplementary material for: Alveolar epithelial paxillin in postnatal lung alveolar development
Source: Biol Open. 2025 Mar 20;14(3):bio061939. doi: 10.1242/bio.061939 (PMC11957453; doi:10.1242/bio.061939)
Supplement: Supplementary information [file biolopen-14-061939-s1.pdf]

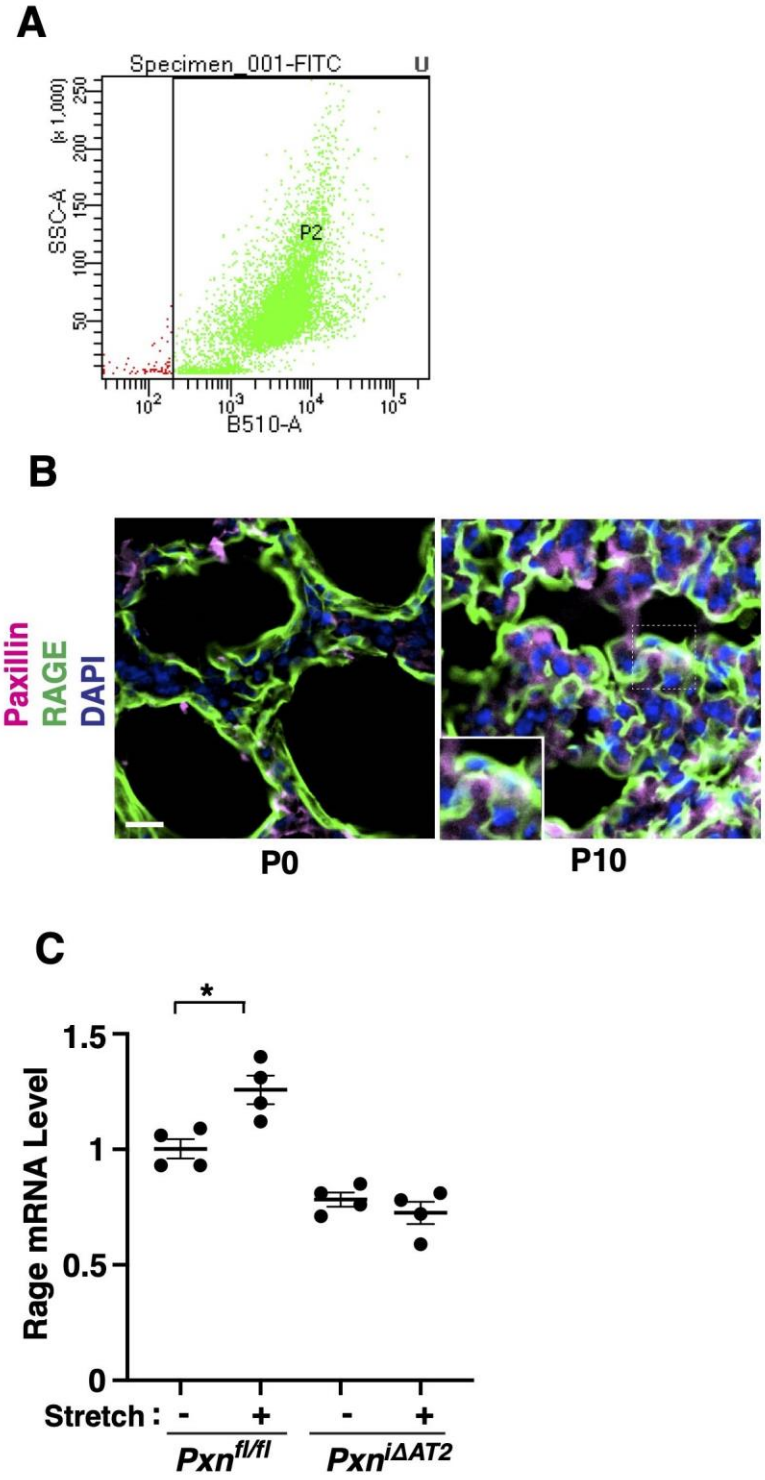
A
Specimen_001-FITC
SSC-A (x 1,000)
B510-A
P2
B
Paxillin RAGE DAPI
P0
P10
C
Rage mRNA Level
Stretch :
Pxn fl/fl
Pxn iΔAT2

Fig. S1. Paxillin expression increases in alveolar epithelial cells during postnatal lung development. A) FACS analysis of alveolar epithelial cells isolated from mouse lungs showing 94.0% of isolated lung cells are EpCAM-positive. **B)** IF micrographs showing RAGE-positive AT1 cells, paxillin expression and DAPI (*insert*: higher magnification image) of P0 and P10 mouse lungs. Scale bars, 25 μm. **C)** mRNA levels of Rage in the P10 $Pxn^{i\Delta AT2}$ and $Pxn^{fl/fl}$ mouse lung $EpCAM^{+}$ cells with or without stretching (n=4, mean ± s.e.m., *p<0.05). Data are analyzed by an unpaired Student *t* test.

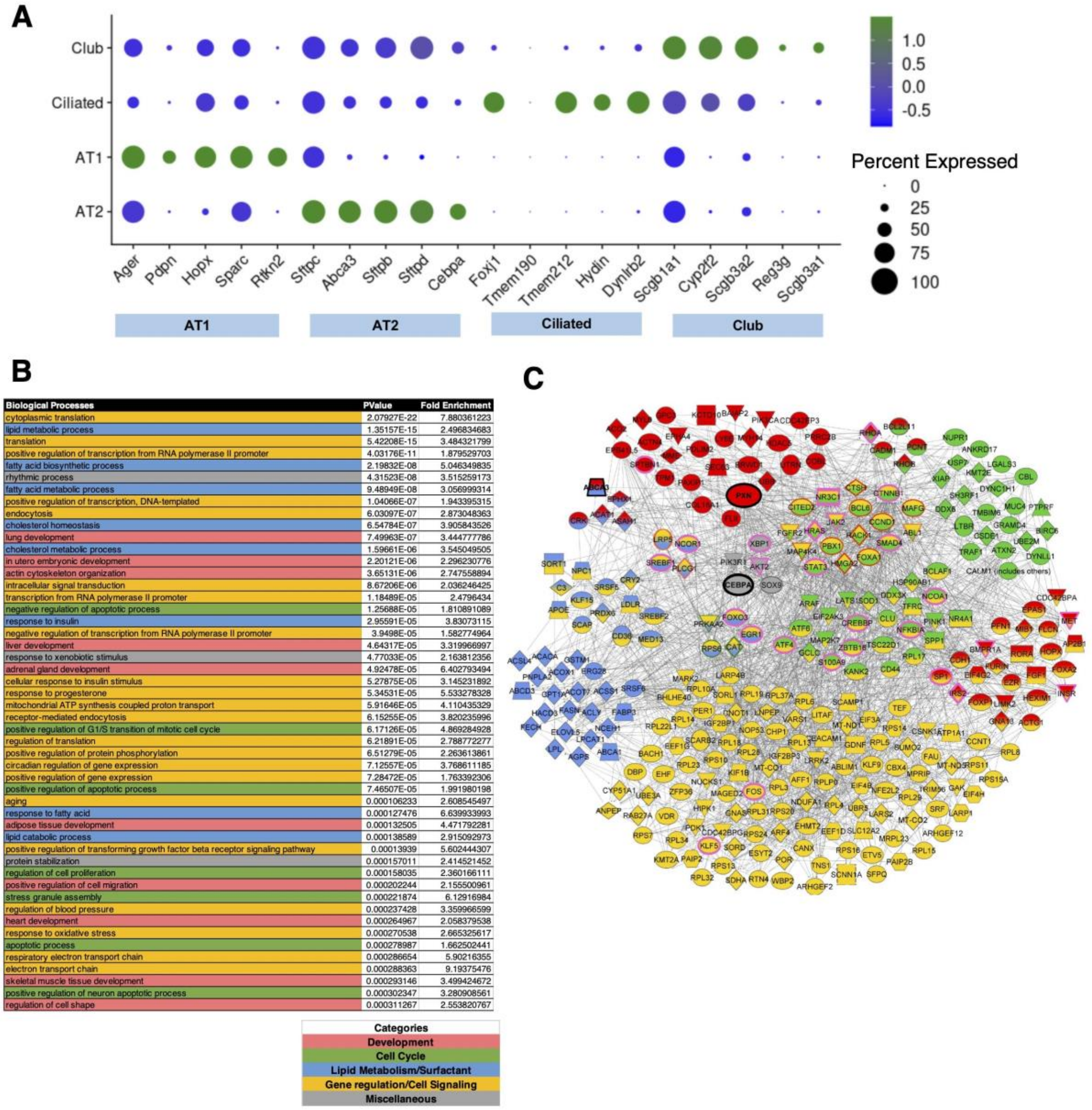

Biological Processes	PValue	Fold Enrichment
cytoplasmic translation	2.07927E-22	7.880361223
lipid metabolic process	1.35157E-15	2.496834683
translation	5.42208E-15	3.484321799
positive regulation of transcription from RNA polymerase II promoter	4.03176E-11	1.879529703
fatty acid biosynthetic process	2.19832E-08	5.046349835
rhythmic process	4.31523E-08	3.515259173
fatty acid metabolic process	9.48949E-08	3.056999314
positive regulation of transcription, DNA-templated	1.04066E-07	1.943395315
endocytosis	6.03097E-07	2.873048363
cholesterol homeostasis	6.54784E-07	3.905843526
lung development	7.49963E-07	3.444777786
cholesterol metabolic process	1.59661E-06	3.545049505
in utero embryonic development	2.20121E-06	2.296230776
actin cytoskeleton organization	3.65131E-06	2.747558894
intracellular signal transduction	8.67206E-06	2.036246425
transcription from RNA polymerase II promoter	1.18489E-05	2.4796434
negative regulation of apoptotic process	1.25688E-05	1.810891089
response to insulin	2.95591E-05	3.83073115
negative regulation of transcription from RNA polymerase II promoter	3.9498E-05	1.582774964
liver development	4.64317E-05	3.319966997
response to xenobiotic stimulus	4.77033E-05	2.163812356
adrenal gland development	4.92478E-05	6.402793494
cellular response to insulin stimulus	5.27875E-05	3.145231892
response to progesterone	5.34531E-05	5.533278328
mitochondrial ATP synthesis coupled proton transport	5.91646E-05	4.110435329
receptor-mediated endocytosis	6.15255E-05	3.820235996
positive regulation of G1/S transition of mitotic cell cycle	6.17126E-05	4.869284928
regulation of translation	6.21891E-05	2.788772277
positive regulation of protein phosphorylation	6.51279E-05	2.263613861
circadian regulation of gene expression	7.12557E-05	3.768611185
positive regulation of gene expression	7.28472E-05	1.763392306
positive regulation of apoptotic process	7.46507E-05	1.991980198
aging	0.000106233	2.608545497
response to fatty acid	0.000127476	6.639933993
adipose tissue development	0.000132505	4.471792281
lipid catabolic process	0.000138589	2.915092973
positive regulation of transforming growth factor beta receptor signaling pathway	0.00013939	5.602444307
protein stabilization	0.000157011	2.414521452
regulation of cell proliferation	0.000158035	2.360166111
positive regulation of cell migration	0.000202244	2.155500961
stress granule assembly	0.000221874	6.12916984
regulation of blood pressure	0.000237428	3.359966599
heart development	0.000264967	2.058379538
response to oxidative stress	0.000270538	2.665325617
apoptotic process	0.000278987	1.662502441
respiratory electron transport chain	0.000286654	5.90216355
electron transport chain	0.000288363	9.19375476
skeletal muscle tissue development	0.000293146	3.499424672
positive regulation of neuron apoptotic process	0.000302347	3.280908561
regulation of cell shape	0.000311267	2.553820767

Categories
Development
Cell Cycle
Lipid Metabolism/Surfactant
Gene regulation/Cell Signaling
Miscellaneous

Fig. S2. scRNAseq analysis of focal adhesion related genes in P3 vs. P14 mouse lung AT2 cells. A) Dot plots of representative marker genes of each epithelial cluster in the mouse lungs. **B)** Top 50 BPGO terms categories of significantly differentially expressed genes in the P3 vs. P14 mouse lung AT2 cell clusters. Pink: Development, Green: Cell cycle, Blue: Lipid metabolism/surfactant, Gold: Gene regulation/Cell signaling, Grey: Miscellaneous. **C)** IPA network analysis showing interactions between paxillin and significantly differentially expressed genes in AT2 cells in the P3 and P14 mouse lungs categorized as development, cell cycle, lipid metabolism/surfactant, gene regulation/cell signaling related genes.

Biology Open • Supplementary information

Table S1.

Gene ID	p_val	avg_log2FC	p_val_adj
Apoe	1.53E-134	1.494518281	3.39E-130
Cbr2	3.82E-185	1.311195424	8.45E-181
Gm42418	1.54E-81	1.168580369	3.40E-77
Scd1	1.79E-65	1.078780863	3.95E-61
Klf9	2.10E-126	1.059048075	4.65E-122
Rgcc	1.93E-78	1.039872822	4.26E-74
Acoxl	4.82E-103	0.973498421	1.07E-98
Epas1	4.62E-109	0.885104723	1.02E-104
Lyz2	5.19E-71	0.873848578	1.15E-66
Glul	1.75E-91	0.85854124	3.87E-87
Lrg1	2.84E-32	0.849861149	6.28E-28
Ces1d	6.20E-107	0.849727068	1.37E-102
Egr1	3.56E-54	0.846425117	7.87E-50
Errfi1	4.51E-82	0.834757267	9.97E-78
Cat	2.94E-100	0.81695372	6.49E-96
Bmp1	6.69E-67	0.795463008	1.48E-62
Slc34a2	4.82E-190	0.787200353	1.07E-185
H2-Ab1	5.68E-66	0.773282842	1.26E-61
Fosb	1.05E-40	0.76772328	2.31E-36
Cpm	1.26E-74	0.75913393	2.79E-70
Clic4	1.23E-72	0.709803432	2.71E-68
Lpin2	3.38E-66	0.698999127	7.47E-62
Atp1b1	6.32E-93	0.693291458	1.40E-88
Lars2	9.70E-50	0.687259219	2.14E-45
Zbtb20	3.02E-63	0.684539246	6.67E-59
Aox3	3.57E-57	0.683699579	7.90E-53
H2-Aa	1.66E-57	0.6834211	3.67E-53
Rnase4	1.13E-81	0.683151111	2.50E-77
mt-Co1	3.41E-188	0.67891938	7.55E-184
Apoc1	6.28E-40	0.66801919	1.39E-35
Herpud1	1.52E-59	0.662303916	3.36E-55
Mme	7.59E-64	0.662034155	1.68E-59
Fam126b	3.78E-48	0.654352035	8.35E-44
Ccdc141	1.48E-54	0.644164357	3.27E-50
Atp7b	6.11E-66	0.639889989	1.35E-61
Nop53	1.02E-60	0.634579555	2.26E-56
Scnn1a	3.65E-57	0.615271707	8.07E-53
Prdx6	4.06E-57	0.612554573	8.98E-53
Selenop	8.23E-61	0.597987037	1.82E-56
Nupr1	2.08E-50	0.587997911	4.60E-46
Abcd3	1.54E-55	0.580177526	3.40E-51
Emb	1.40E-51	0.573826143	3.10E-47
Ahcyl2	9.06E-44	0.571892678	2.00E-39
Kcnj15	2.30E-50	0.570726039	5.09E-46
Per1	5.09E-55	0.568864237	1.13E-50
Arhgef38	1.35E-48	0.563109521	2.98E-44

Neat1	6.55E-35	0.560652895	1.45E-30
Mmp15	1.23E-48	0.538842655	2.72E-44
Eln	2.92E-50	0.537163003	6.45E-46
Fasn	4.83E-40	0.519072105	1.07E-35
Snx25	4.76E-41	0.514314762	1.05E-36
Serinc3	3.37E-42	0.511787088	7.45E-38
Slc6a6	2.18E-36	0.502311874	4.82E-32
Chia1	2.53E-14	0.497862182	5.58E-10
mt-Nd3	2.68E-46	0.495214669	5.93E-42
Sec14l4	1.42E-40	0.491664089	3.13E-36
Mgp	9.02E-36	0.488038303	1.99E-31
Atf4	4.44E-34	0.483644585	9.80E-30
Slc43a2	1.20E-32	0.483618375	2.66E-28
Zfp36	3.23E-29	0.482849563	7.14E-25
Sod1	1.93E-41	0.482517726	4.27E-37
Lgi3	5.53E-45	0.482011372	1.22E-40
Dazap2	8.32E-40	0.47921628	1.84E-35
Abca3	1.85E-55	0.478101957	4.09E-51
Deptor	2.88E-37	0.477181848	6.37E-33
Enpep	3.60E-33	0.475798069	7.95E-29
Nfix	4.28E-36	0.469700447	9.46E-32
Por	7.89E-35	0.465829353	1.74E-30
C3	1.73E-31	0.4652949	3.82E-27
2810474O19Rik	5.69E-26	0.464596774	1.26E-21
Megf6	8.71E-46	0.45886837	1.93E-41
Adgrf5	4.82E-38	0.457621175	1.06E-33
Ank3	1.48E-40	0.457551487	3.27E-36
Bach1	5.17E-37	0.456847083	1.14E-32
Foxp1	4.47E-38	0.451074796	9.87E-34
Ddx3x	7.80E-34	0.445915496	1.72E-29
Akr1b3	4.23E-31	0.445173286	9.34E-27
mt-Nd5	1.99E-44	0.444460325	4.40E-40
Lmo7	2.79E-25	0.443992892	6.16E-21
Lamp3	5.01E-44	0.431462626	1.11E-39
Pabpc1	4.15E-36	0.430590963	9.18E-32
Cox6a2	4.40E-21	0.42702905	9.72E-17
Irs2	3.10E-23	0.418067297	6.86E-19
Tfrc	7.21E-28	0.417925362	1.59E-23
mt-Nd1	5.82E-58	0.416939385	1.29E-53
Cd74	1.11E-36	0.412232096	2.45E-32
Hp	1.23E-26	0.411825908	2.71E-22
Itih4	2.93E-19	0.408872328	6.47E-15
Ralgapa2	6.85E-27	0.408200046	1.51E-22
Srebf1	6.05E-23	0.407080799	1.34E-18
Txn1	2.87E-34	0.401822694	6.35E-30
Ctnnb1	8.13E-34	0.399918382	1.80E-29
Rora	2.00E-25	0.39901759	4.43E-21

Biology Open • Supplementary information

Cdc42bpa	5.23E-26	0.397340545	1.16E-21
Creg1	4.72E-28	0.391466074	1.04E-23
mt-Nd4	2.96E-66	0.391083871	6.54E-62
Larp4b	9.73E-27	0.390352356	2.15E-22
Cd36	5.94E-33	0.389928827	1.31E-28
5330417C22Rik	2.06E-25	0.386247649	4.55E-21
Uhrf1bp1l	1.46E-27	0.385035107	3.22E-23
Irf2bp2	6.31E-26	0.382944983	1.39E-21
Ly6e	1.68E-20	0.378516225	3.71E-16
Nfic	7.21E-25	0.37666106	1.59E-20
Tacc1	2.96E-26	0.375219503	6.54E-22
Rpl5	3.93E-43	0.374658483	8.69E-39
Dst	4.32E-24	0.374013347	9.55E-20
Sord	2.66E-23	0.373198869	5.87E-19
Mkln1	1.58E-23	0.372873798	3.49E-19
Mob1b	4.06E-23	0.37236626	8.98E-19
Mllt6	1.57E-26	0.371872504	3.47E-22
Gas6	2.47E-26	0.366911861	5.46E-22
mt-Co2	2.83E-62	0.362133068	6.26E-58
Itga9	5.73E-21	0.362009683	1.27E-16
Dusp1	1.12E-16	0.361974893	2.47E-12
Brd2	3.19E-19	0.360647237	7.06E-15
AA986860	1.12E-25	0.359193416	2.47E-21
Tns1	7.34E-24	0.35900545	1.62E-19
Eif3a	9.62E-25	0.358461489	2.13E-20
Sult1a1	5.21E-26	0.358429407	1.15E-21
Adam19	3.53E-21	0.357718527	7.81E-17
Irx1	3.52E-24	0.35728641	7.77E-20
H2-Eb1	6.65E-22	0.354133534	1.47E-17
Tef	1.36E-26	0.349926954	3.00E-22
Acot7	2.75E-17	0.349856215	6.08E-13
Socs2	1.51E-18	0.347510844	3.34E-14
Pnpla2	4.71E-22	0.347443912	1.04E-17
Cebpa	1.63E-19	0.347381801	3.60E-15
Nfia	2.57E-21	0.346986037	5.68E-17
Baiap2	7.55E-20	0.346821022	1.67E-15
Hnrnph1	5.59E-23	0.345215248	1.23E-18
Itpr2	4.02E-33	0.344438299	8.88E-29
Foxa2	1.56E-19	0.34367763	3.45E-15
Secisbp2l	2.07E-21	0.342478216	4.57E-17
Etv5	1.07E-20	0.342464292	2.36E-16
Scd2	2.02E-18	0.342084467	4.46E-14
Atp8a1	8.95E-28	0.3420263	1.98E-23
Snhg11	7.18E-22	0.340005148	1.59E-17
Kank2	3.45E-21	0.3385074	7.63E-17
Use1	6.44E-25	0.336818633	1.42E-20
Met	8.89E-20	0.336778107	1.96E-15

Biology Open • Supplementary information

Slc31a1	1.86E-18	0.333017394	4.11E-14
C2cd4b	1.01E-13	0.332696851	2.23E-09
Ldlr	1.98E-14	0.331779237	4.38E-10
Foxo3	1.32E-21	0.331761234	2.91E-17
Gpt	2.76E-24	0.330139065	6.10E-20
C6	3.42E-15	0.33003728	7.56E-11
Khk	2.66E-22	0.329833438	5.88E-18
Fam168b	1.03E-20	0.329593532	2.28E-16
Anpep	3.02E-27	0.32788912	6.67E-23
Csgalnact2	4.86E-21	0.326709949	1.07E-16
Ehf	1.85E-21	0.323952105	4.09E-17
Tsc22d3	8.56E-18	0.323842129	1.89E-13
Tmprss4	3.03E-20	0.322808674	6.69E-16
Cds1	9.30E-22	0.322563921	2.05E-17
Actn1	4.76E-20	0.321559315	1.05E-15
Zbtb16	9.27E-40	0.320931359	2.05E-35
Hexim1	3.37E-18	0.320420259	7.44E-14
Slco4c1	2.59E-20	0.32040339	5.72E-16
Birc6	1.05E-16	0.319061969	2.33E-12
Rab27a	4.34E-17	0.318143474	9.58E-13
Gns	3.85E-21	0.315121045	8.51E-17
Srebf2	4.51E-19	0.313595919	9.96E-15
Lrrk2	3.43E-19	0.313160057	7.58E-15
Ctnnal1	3.28E-21	0.312379585	7.24E-17
Mid1ip1	1.52E-13	0.311947596	3.36E-09
Ttc38	6.22E-21	0.310750103	1.37E-16
Pde7a	3.89E-18	0.3105164	8.60E-14
Cd200	1.77E-19	0.309797826	3.91E-15
Cd44	2.64E-21	0.309367157	5.84E-17
Cracr2a	2.20E-18	0.309196111	4.86E-14
Gtf2i	3.78E-18	0.309069534	8.35E-14
Mthfd1	9.55E-21	0.30846195	2.11E-16
C77080	3.69E-18	0.307002207	8.15E-14
Eef2	1.92E-26	0.306452135	4.24E-22
Ssh2	2.09E-17	0.306072412	4.62E-13
Iah1	5.28E-19	0.30429356	1.17E-14
Ddx17	3.33E-17	0.303836971	7.37E-13
Atp1a1	1.06E-19	0.301875416	2.35E-15
Npc1	1.81E-18	0.300194522	4.00E-14
Atp6v1a	1.39E-17	0.299376512	3.07E-13
2900097C17Rik	1.63E-17	0.29882373	3.61E-13
Pum2	3.07E-19	0.298684712	6.79E-15
Pex11a	3.76E-20	0.298576124	8.32E-16
Sgpp2	6.99E-18	0.29730859	1.55E-13
Diaph1	5.82E-18	0.296851062	1.29E-13
Atp11a	3.79E-18	0.296119647	8.38E-14
Abca1	6.52E-27	0.29608311	1.44E-22

Biology Open • Supplementary information

Tob2	7.55E-18	0.294568643	1.67E-13
Aff1	5.37E-17	0.291430394	1.19E-12
Sec61a1	1.73E-14	0.29139969	3.81E-10
Entpd1	6.34E-20	0.290381249	1.40E-15
Ppp1r1b	6.75E-28	0.290380068	1.49E-23
Acaca	1.27E-17	0.290035157	2.80E-13
Kctd10	4.82E-19	0.289874746	1.07E-14
Rps6	1.48E-24	0.289179257	3.27E-20
Tspan11	1.23E-16	0.28910633	2.72E-12
Slc22a23	7.78E-17	0.289050771	1.72E-12
Lrp2	8.73E-16	0.289016359	1.93E-11
Cobl	2.03E-19	0.288013451	4.49E-15
Zfp503	4.76E-13	0.287589589	1.05E-08
Bckdha	9.57E-22	0.287582734	2.11E-17
Per3	6.11E-20	0.287416251	1.35E-15
Prr15l	7.60E-17	0.286606574	1.68E-12
Cited2	2.82E-13	0.286021665	6.23E-09
Nisch	5.00E-15	0.285711998	1.10E-10
Insr	1.90E-18	0.285646457	4.19E-14
Pik3r1	1.13E-16	0.285247447	2.50E-12
Tor1aip1	2.50E-16	0.284451418	5.53E-12
BC005537	2.39E-17	0.283451394	5.28E-13
Chil3	4.95E-35	0.283052871	1.09E-30
Eif4h	2.30E-15	0.282914966	5.09E-11
Acsl5	8.23E-18	0.281519963	1.82E-13
Mknk2	4.64E-17	0.280052598	1.02E-12
Macc1	1.31E-17	0.280040032	2.90E-13
Gclc	7.27E-10	0.279110885	1.61E-05
Lifr	6.14E-17	0.278827539	1.36E-12
B4galnt1	6.25E-26	0.278753584	1.38E-21
Zbtb4	1.32E-18	0.27858798	2.92E-14
Slc15a2	5.92E-16	0.278509836	1.31E-11
Phldb2	3.17E-16	0.278377038	7.02E-12
Prrg3	1.01E-12	0.277912318	2.24E-08
Iqgap1	1.11E-21	0.273900947	2.45E-17
Rpl17	1.54E-26	0.27297292	3.40E-22
Cyb5b	1.56E-16	0.272823769	3.45E-12
Gprc5a	6.52E-16	0.271997636	1.44E-11
Rab3a	1.40E-16	0.271719594	3.10E-12
Fos	8.12E-13	0.271284629	1.79E-08
Atp6v1b2	1.97E-13	0.271215617	4.35E-09
Fhdc1	3.16E-17	0.270882723	6.99E-13
Ptprf	2.26E-20	0.269107074	4.99E-16
Man1a	9.10E-16	0.267807911	2.01E-11
Sec14l3	3.89E-12	0.267658365	8.59E-08
Prmt8	3.12E-29	0.267336379	6.89E-25
Micall1	5.12E-20	0.266870392	1.13E-15

mt-Atp6	1.40E-41	0.26666254	3.09E-37
Chp1	2.85E-15	0.265554082	6.30E-11
Mbnl1	1.00E-17	0.265331044	2.22E-13
Tbcel	3.05E-14	0.265295632	6.74E-10
Acss1	4.66E-16	0.264669493	1.03E-11
Ddhd1	3.44E-14	0.264115806	7.61E-10
Prcd	1.35E-19	0.263560884	2.98E-15
Aldh6a1	2.11E-15	0.262896064	4.67E-11
Chka	9.74E-11	0.261500901	2.15E-06
mt-Nd2	7.59E-29	0.260367206	1.68E-24
Dyrk2	3.01E-15	0.260114012	6.65E-11
Adipor2	1.87E-14	0.259081766	4.12E-10
Rbms3	8.21E-14	0.258996389	1.81E-09
D230025D16Rik	1.47E-13	0.258897534	3.26E-09
Mbd1	3.48E-13	0.258808992	7.69E-09
Acsl4	3.26E-14	0.25821646	7.21E-10
Ctdsp2	3.53E-14	0.257478171	7.79E-10
Bicc1	6.18E-16	0.257457406	1.36E-11
Rassf3	7.49E-15	0.257027475	1.65E-10
Fbxo9	1.67E-14	0.256998327	3.68E-10
Cdc42ep3	1.11E-11	0.256773878	2.45E-07
Akt2	2.09E-15	0.256687836	4.61E-11
Hmgcs2	5.98E-19	0.256457219	1.32E-14
Larp1	2.05E-12	0.256354887	4.53E-08
Ppl	1.33E-13	0.255729051	2.94E-09
Mical2	2.43E-12	0.255178362	5.36E-08
Klf13	9.63E-14	0.255050444	2.13E-09
Fads1	2.25E-13	0.25479135	4.97E-09
Atp5a1	1.22E-17	0.253672303	2.70E-13
Pbx1	7.24E-11	0.253107621	1.60E-06
Scaf11	5.64E-13	0.251696179	1.25E-08
Bhlhe40	3.74E-11	0.251604438	8.27E-07
Xdh	1.09E-21	0.25119052	2.42E-17
Cdc42bpg	1.29E-18	0.250524174	2.84E-14
Rbm47	1.38E-12	0.250176787	3.05E-08
Bambi	4.12E-09	0.250014243	9.10E-05
Acox1	3.83E-14	0.249665637	8.46E-10
Plekhb2	8.35E-15	0.249518173	1.84E-10
Pura	2.70E-12	0.248912912	5.98E-08
Nr4a1	5.91E-07	0.248385236	0.013067463
Prps2	2.53E-12	0.247047998	5.59E-08
Cdh1	2.06E-18	0.246977792	4.55E-14
Acly	2.10E-13	0.246856073	4.64E-09
Ogdh	1.62E-13	0.24676313	3.57E-09
Ptgs1	6.32E-12	0.246068733	1.40E-07
Thsd4	1.72E-25	0.245916228	3.79E-21
Ahnak	3.98E-12	0.245709248	8.80E-08

Col4a4	4.91E-14	0.245634882	1.08E-09
Nceh1	4.64E-11	0.245008036	1.03E-06
Suclg1	1.07E-13	0.244936889	2.37E-09
Alcam	4.57E-18	0.244883446	1.01E-13
Nectin1	1.89E-12	0.244819102	4.17E-08
Gstt3	8.69E-14	0.244531219	1.92E-09
Etnk1	1.38E-12	0.244488816	3.05E-08
Nrbp2	2.36E-13	0.244291501	5.23E-09
Cry2	1.82E-11	0.244194961	4.01E-07
Slco2a1	5.64E-11	0.243893361	1.25E-06
Gramd2	1.44E-10	0.243082041	3.18E-06
Rbms1	4.00E-13	0.242916639	8.85E-09
Cdip1	5.27E-13	0.242186927	1.17E-08
Sf3b2	3.18E-10	0.241169043	7.02E-06
Bclaf1	2.52E-13	0.2405413	5.57E-09
Nedd4l	1.23E-12	0.239365362	2.72E-08
Stat3	2.05E-10	0.239152201	4.54E-06
Arhgef12	6.68E-11	0.23848561	1.48E-06
Mlc1	4.35E-12	0.237947065	9.62E-08
Eef1d	2.72E-15	0.237821154	6.01E-11
Fam129a	1.36E-15	0.237713291	3.01E-11
Atp6v1g1	1.08E-13	0.23633198	2.38E-09
Pank3	3.89E-13	0.235871509	8.59E-09
Mettl7a1	1.74E-13	0.235796312	3.85E-09
Hnrnpa3	7.40E-13	0.235023977	1.64E-08
Igfbp6	6.47E-13	0.23450871	1.43E-08
Cluh	1.21E-17	0.234485075	2.67E-13
Prrc2b	6.79E-12	0.232725936	1.50E-07
Dhrs3	5.45E-13	0.232414816	1.21E-08
Gsta3	3.81E-21	0.232020128	8.42E-17
Fbrsl1	2.28E-13	0.231891068	5.04E-09
Dbp	7.62E-11	0.231641301	1.69E-06
Gstp3	1.47E-12	0.231324587	3.26E-08
Man2a1	6.55E-11	0.231096936	1.45E-06
Gstm1	3.73E-16	0.230351948	8.24E-12
Paxip1	1.27E-17	0.22891926	2.81E-13
Sytl2	6.26E-12	0.228456002	1.38E-07
Slc12a2	1.87E-13	0.22753371	4.14E-09
Tmod1	1.62E-14	0.227484974	3.57E-10
Pfkfb2	2.36E-15	0.227082163	5.21E-11
Pik3ca	2.17E-12	0.227066126	4.79E-08
Trpm6	2.59E-14	0.226510812	5.71E-10
Acer3	4.32E-15	0.22646561	9.56E-11
Ash1l	1.05E-11	0.226411648	2.33E-07
Adcy7	1.18E-11	0.225060637	2.60E-07
Mpv17l2	1.21E-11	0.22413893	2.67E-07
Gnpat	9.71E-12	0.224094612	2.15E-07

Pccb	1.89E-14	0.223888239	4.17E-10
Fmo2	1.23E-17	0.223144335	2.72E-13
Tet3	2.52E-10	0.223052525	5.56E-06
Wbp2	2.53E-10	0.222615304	5.59E-06
Srsf5	8.02E-13	0.221624131	1.77E-08
Tspan2	2.42E-11	0.221609112	5.36E-07
Creb3l1	3.65E-13	0.221587869	8.08E-09
Cobll1	5.73E-14	0.221013342	1.27E-09
Kif1b	8.51E-10	0.220507678	1.88E-05
S100a10	2.15E-10	0.22039352	4.76E-06
Muc1	1.08E-14	0.21967851	2.38E-10
Tmem245	7.90E-12	0.218790084	1.75E-07
Erbin	2.46E-09	0.218673412	5.44E-05
Nfkbiz	6.61E-09	0.218321389	0.000146185
Slc30a5	1.24E-12	0.218068673	2.73E-08
Cflar	1.66E-10	0.217394564	3.68E-06
Npr1	2.11E-13	0.216639941	4.67E-09
Cpeb4	1.08E-10	0.216265013	2.39E-06
Atxn2l	1.07E-10	0.216247559	2.36E-06
Gigyf1	5.41E-13	0.216074251	1.20E-08
Per2	4.36E-11	0.215694108	9.64E-07
Rassf2	1.24E-14	0.21536209	2.75E-10
Megf9	4.16E-12	0.215236902	9.20E-08
Selenbp1	4.81E-10	0.214950695	1.06E-05
Ewsr1	1.41E-10	0.21477738	3.12E-06
mt-Co3	2.94E-25	0.214563718	6.50E-21
Csde1	1.75E-12	0.214323044	3.87E-08
Scarf2	1.64E-12	0.213356367	3.62E-08
Smg1	5.61E-13	0.21300445	1.24E-08
Tbc1d4	9.51E-11	0.212960701	2.10E-06
Pias1	1.36E-10	0.212537032	3.00E-06
6-Mar	1.79E-12	0.212380384	3.96E-08
Mtss1	7.26E-11	0.212173124	1.60E-06
Xbp1	8.19E-10	0.211929759	1.81E-05
Zfp36l2	1.55E-09	0.211833211	3.43E-05
Trove2	2.12E-10	0.211588636	4.68E-06
Hip1r	1.27E-10	0.211188914	2.80E-06
Pmm1	1.75E-12	0.210494661	3.87E-08
Ltbr	9.89E-12	0.210401568	2.19E-07
Usp25	5.86E-12	0.209372256	1.30E-07
Sf1	5.60E-09	0.209307107	0.000123686
Lpcat1	1.15E-14	0.20911634	2.53E-10
Nfe2l1	4.84E-10	0.209077485	1.07E-05
Kdm6b	4.72E-11	0.208762038	1.04E-06
Golph3	5.14E-12	0.208716728	1.14E-07
Ddi2	3.24E-10	0.208661711	7.16E-06
Acaa2	8.33E-12	0.207632107	1.84E-07

Zfp110	1.59E-12	0.20724682	3.52E-08
Col23a1	1.45E-11	0.207037308	3.20E-07
Sp1	7.29E-11	0.206867096	1.61E-06
Numa1	2.02E-12	0.205995438	4.46E-08
Fus	1.48E-11	0.20585337	3.27E-07
Rhob	6.88E-08	0.205779856	0.001520883
Epn2	1.25E-10	0.205715725	2.77E-06
Cpt1a	9.79E-12	0.205217091	2.16E-07
Fkbp15	2.05E-12	0.205058824	4.53E-08
Syne2	1.84E-09	0.204922695	4.06E-05
Spry1	7.68E-10	0.204720651	1.70E-05
Mib1	2.70E-09	0.204149818	5.96E-05
Ppp1r15b	2.19E-12	0.203973258	4.84E-08
Pptc7	2.77E-11	0.203886382	6.12E-07
Ptp4a1	6.06E-09	0.203666429	0.000133981
Plekhb1	2.13E-13	0.203566756	4.70E-09
Dusp18	1.25E-09	0.203320112	2.76E-05
Hk2	2.98E-11	0.203035316	6.59E-07
Cracr2b	2.25E-12	0.202247053	4.97E-08
Xpot	1.76E-10	0.202129078	3.89E-06
Pink1	3.57E-10	0.202031957	7.90E-06
Kmt2a	3.48E-10	0.202021202	7.70E-06
Slc25a51	1.27E-09	0.201792006	2.82E-05
Dopey1	1.72E-11	0.201684454	3.80E-07
Prpf18	2.97E-10	0.2016251	6.56E-06
Ap2b1	4.34E-08	0.201345185	0.000960199
Trp53inp1	1.01E-10	0.201267101	2.24E-06
Fam91a1	2.03E-11	0.200853432	4.48E-07
Wwc1	4.75E-13	0.200632194	1.05E-08
Ccpg1	6.54E-13	0.200372298	1.45E-08
Klf15	9.20E-14	0.20024756	2.03E-09
Tjp2	5.45E-09	0.200143065	0.000120494
Sftpb	6.24E-27	0.200078373	1.38E-22
Tmem41b	6.63E-09	0.199758129	0.000146595
Klhl29	1.16E-16	0.199399321	2.55E-12
Snrk	8.24E-11	0.199033104	1.82E-06
Hipk1	3.12E-10	0.198664106	6.90E-06
Tspan12	1.65E-10	0.198608799	3.65E-06
Mbnl2	5.39E-11	0.198547421	1.19E-06
Spred2	3.09E-12	0.198547176	6.83E-08
Acat1	1.29E-09	0.198441469	2.86E-05
Slc6a14	5.35E-13	0.198383514	1.18E-08
Lrp5	1.23E-12	0.198304055	2.73E-08
Jak2	1.17E-12	0.198143855	2.58E-08
Raph1	1.64E-09	0.197921571	3.63E-05
Foxa1	1.79E-08	0.197644518	0.000394603
Peli1	1.12E-08	0.197310823	0.000246576

Cytip	2.86E-09	0.196942517	6.31E-05
Ece1	1.37E-09	0.196478932	3.02E-05
Npr2	2.00E-13	0.196377246	4.42E-09
Vldlr	2.39E-08	0.195434798	0.000529188
Zzef1	4.71E-11	0.195432721	1.04E-06
Rbp4	1.20E-16	0.19516817	2.64E-12
Abhd2	3.35E-10	0.19502363	7.41E-06
Crebbp	7.02E-11	0.19497265	1.55E-06
9330182L06Rik	1.02E-11	0.194878378	2.25E-07
Mut	8.56E-11	0.194563573	1.89E-06
Pcnt	1.92E-08	0.194352172	0.000423381
Lrrc58	6.71E-09	0.193640469	0.000148284
Sort1	1.95E-08	0.193407362	0.000430561
Camsap1	2.60E-10	0.193213779	5.75E-06
Ddx6	1.57E-09	0.192970322	3.47E-05
Nucks1	1.46E-11	0.192838046	3.22E-07
Cpd	3.42E-11	0.1925959	7.55E-07
Aco2	1.74E-09	0.19258655	3.86E-05
Cadm1	1.07E-09	0.192450656	2.37E-05
Rabac1	8.84E-12	0.192152837	1.95E-07
Plcg1	2.56E-10	0.191807302	5.66E-06
H6pd	1.07E-07	0.191443769	0.002362981
Prkaa2	9.56E-13	0.191405138	2.11E-08
Tmem109	5.92E-09	0.191199555	0.000130928
Klf5	1.12E-07	0.190616097	0.002466222
Rnf186	5.78E-10	0.19035872	1.28E-05
Pof1b	2.52E-09	0.189883726	5.57E-05
Tfcp2l1	1.03E-08	0.189843472	0.000228087
Ppp2r1a	3.60E-10	0.189284702	7.95E-06
Tmem132d	1.20E-13	0.189128046	2.65E-09
Zcchc14	1.63E-10	0.188436154	3.60E-06
Bmp3	6.84E-10	0.188297628	1.51E-05
Dram1	3.60E-14	0.188280874	7.95E-10
Gse1	2.72E-08	0.188101695	0.000601552
Eif4g1	7.11E-09	0.188086371	0.00015716
Hnrnpul2	4.56E-09	0.18781716	0.00010071
Lrp6	3.89E-10	0.187764564	8.60E-06
Nr1d2	2.06E-10	0.187427232	4.56E-06
Slc16a2	3.12E-09	0.187023248	6.90E-05
Mgat1	2.43E-09	0.186835417	5.37E-05
Itga1	2.71E-15	0.18640317	6.00E-11
Lnpep	1.23E-09	0.186354758	2.71E-05
Cbx7	2.62E-13	0.186000788	5.79E-09
Arid2	2.51E-08	0.185948885	0.000555159
Limk2	8.90E-10	0.18556862	1.97E-05
Med13	1.27E-09	0.184844241	2.81E-05
Zfp395	5.72E-12	0.184740604	1.26E-07

Biology Open • Supplementary information

Cnppd1	1.71E-09	0.184378487	3.77E-05
D17Wsu92e	2.57E-09	0.183996849	5.67E-05
Ankrd17	1.57E-08	0.183912528	0.000346207
Prss8	1.71E-08	0.18358649	0.000377038
Zmiz1	3.19E-08	0.183532698	0.000704794
Foxp4	5.78E-10	0.183465035	1.28E-05
Dixdc1	9.31E-13	0.183452325	2.06E-08
Dync1h1	2.13E-08	0.183384581	0.000470106
Sdha	1.89E-09	0.183356299	4.17E-05
Smim1	5.31E-08	0.183102657	0.001174598
Dgkd	6.50E-09	0.183101471	0.000143624
Klhl21	1.99E-09	0.182809245	4.39E-05
Limch1	8.42E-09	0.182462213	0.000186084
Tbc1d30	1.26E-10	0.182415721	2.79E-06
Scap	8.00E-13	0.182243249	1.77E-08
Tacc2	2.18E-09	0.182135041	4.81E-05
Osgin1	8.36E-13	0.181633173	1.85E-08
Sel1l	8.49E-09	0.181572157	0.000187577
Setd1b	1.46E-08	0.181250652	0.000321597
Trim56	4.51E-10	0.181212944	9.97E-06
Nucb2	9.56E-10	0.180809026	2.11E-05
Tmem8	7.01E-09	0.180796181	0.000154884
Trap1	1.88E-09	0.180707781	4.16E-05
Arid1b	1.14E-08	0.180559809	0.000251072
Mapre2	2.28E-09	0.180225559	5.04E-05
Wdr26	2.87E-09	0.18020977	6.35E-05
Gfpt1	7.16E-08	0.179602501	0.001582117
Smad4	3.92E-08	0.179579921	0.000865394
Atxn7	4.81E-08	0.179408244	0.001062783
Pdha1	4.58E-09	0.178701778	0.000101197
Litaf	6.20E-10	0.177930996	1.37E-05
Hdac5	2.76E-08	0.177892682	0.000609365
Krt23	1.54E-08	0.177838939	0.00034031
Rnf145	6.10E-10	0.177780423	1.35E-05
Urah	2.87E-09	0.177668157	6.35E-05
Plxna4	2.57E-10	0.177605721	5.68E-06
Ggcx	4.00E-09	0.177489093	8.85E-05
Gdpd1	1.24E-07	0.177452084	0.002742663
Fgd6	5.31E-09	0.177396858	0.000117422
Tmem56	6.88E-10	0.177314753	1.52E-05
Eif4a2	2.10E-10	0.177100718	4.63E-06
Pcyt1a	1.47E-06	0.176565104	0.032447181
Golgb1	2.10E-07	0.176511352	0.004634537
Usp7	1.75E-08	0.176349396	0.000385928
Ezr	2.97E-09	0.175919773	6.57E-05
Ncoa1	3.38E-08	0.175788438	0.000747767
Traf1	2.30E-08	0.175730415	0.000508754

Pdcd6ip	1.34E-07	0.175387884	0.002955519
Arrdc3	3.01E-07	0.17538613	0.006654893
Myo5b	1.23E-08	0.175019176	0.000271095
H13	1.59E-07	0.174962941	0.003519187
Rai1	8.31E-09	0.174799054	0.000183638
Kat2b	4.31E-09	0.174572721	9.53E-05
Ccnt2	8.49E-08	0.174521829	0.001875222
Irx2	5.67E-10	0.173986101	1.25E-05
Col4a3bp	1.03E-08	0.173702298	0.000227909
Clmn	9.68E-08	0.173674087	0.002139357
Fzd5	1.62E-08	0.173540393	0.000357214
Snx27	5.81E-09	0.173505524	0.000128336
Vezf1	8.32E-09	0.173281428	0.000183809
Nktr	2.53E-07	0.173099259	0.005600422
Usp33	1.97E-06	0.172575824	0.043527648
Sec63	9.88E-08	0.172241302	0.002182469
Lats1	1.48E-08	0.172177712	0.000327475
Ranbp10	4.80E-09	0.172133604	0.000105993
Nkd1	1.32E-07	0.171877037	0.002924988
Rab11fip2	1.81E-07	0.171715001	0.00399829
Zdhhc3	5.56E-09	0.171486055	0.000122861
Prpf8	2.66E-08	0.171358272	0.000587469
Ttc39b	2.31E-09	0.17127055	5.11E-05
Tmem164	6.18E-08	0.171269636	0.001365188
Clstn1	1.63E-07	0.171088064	0.003605569
Cfap20	1.36E-07	0.171003688	0.002998849
Xiap	1.92E-09	0.170989345	4.25E-05
Arih1	1.59E-07	0.170903212	0.003503153
Mark2	4.22E-08	0.170786358	0.000933286
mt-Cytb	1.96E-17	0.17071183	4.34E-13
Ldb1	3.29E-10	0.170540806	7.27E-06
Rpn1	1.39E-07	0.170427237	0.00307506
Ranbp2	3.80E-08	0.169955305	0.00084083
Rprm	7.11E-07	0.169931365	0.015719819
Flii	3.46E-08	0.169755225	0.00076553
Ppp6r3	4.28E-08	0.169492695	0.000946642
Tapbp	1.98E-07	0.169392786	0.004369829
Mafg	8.45E-07	0.16936138	0.018675929
Myh14	2.77E-08	0.169348985	0.000613128
Fgfr2	2.75E-08	0.169189576	0.000607781
Igsf3	3.08E-08	0.168846241	0.000681516
Ptpn13	6.12E-09	0.168708028	0.000135329
Agps	3.18E-08	0.16852669	0.000701838
Pik3c2b	7.07E-12	0.168234878	1.56E-07
Mtm1	6.15E-10	0.168227113	1.36E-05
Arl8b	1.77E-07	0.168053314	0.003901424
Ablim1	1.57E-07	0.167762796	0.003480534

Biology Open • Supplementary information

Cecr2	5.92E-08	0.16763036	0.001308978
Gdnf	1.42E-11	0.167607802	3.13E-07
Pon1	1.34E-08	0.16735675	0.00029681
Mprip	1.41E-07	0.16735234	0.003108245
Pja2	3.64E-07	0.16704109	0.008044126
Rpl10-ps3	7.01E-08	0.166721255	0.001548498
Bcl2l11	2.24E-08	0.166709102	0.00049538
Utrn	3.73E-07	0.166670856	0.008253361
Zc3h7a	9.40E-09	0.166583321	0.00020765
Msl2	1.93E-08	0.16655108	0.000425738
Gna13	6.45E-08	0.166533707	0.001426409
Srsf6	1.07E-08	0.166445379	0.000236421
Map4k4	2.68E-07	0.165985958	0.005929773
Myo5c	2.20E-08	0.165943244	0.000485576
Pds5a	4.31E-07	0.165437806	0.009531112
Phf2	2.35E-08	0.165313462	0.000519108
Eif4g3	2.04E-07	0.164840991	0.004497445
Atp6v1c2	2.46E-07	0.164668759	0.005435273
Pxmp2	1.01E-10	0.164444488	2.23E-06
Srf	3.16E-08	0.164431787	0.000699373
Comt	1.83E-07	0.164322876	0.004043133
Amfr	9.60E-08	0.164143179	0.00212205
Pip4k2c	9.15E-09	0.163935725	0.000202308
Fut8	5.19E-07	0.163897575	0.011471435
Tnk2	9.23E-11	0.163583145	2.04E-06
Hacd3	2.98E-07	0.163386267	0.006577861
Vps54	2.32E-08	0.163297488	0.000513496
Sft2d2	4.43E-08	0.163287799	0.000980024
Canx	9.02E-09	0.163228042	0.000199412
Purb	2.83E-08	0.162992112	0.000625816
Ptpn3	1.01E-10	0.16292895	2.24E-06
Ccp110	2.04E-08	0.162911256	0.000450822
Slc26a2	2.80E-08	0.162714193	0.000619165
Sptlc1	2.17E-08	0.162684235	0.000479305
Rps27rt	5.62E-07	0.162438791	0.012430263
Csnk1e	2.83E-07	0.162317001	0.006243898
Cisd1	1.34E-07	0.162284518	0.002954827
Atf6	1.97E-09	0.162213652	4.35E-05
2810403A07Rik	1.39E-07	0.161965536	0.003079397
Nbr1	1.08E-07	0.161926421	0.002380027
Cab39l	5.52E-08	0.161692999	0.001219743
Pdk1	9.25E-09	0.161467256	0.000204318
Selenos	5.50E-09	0.161339172	0.000121494
Acadsb	1.03E-07	0.161268877	0.002276163
Brwd1	1.04E-06	0.161121148	0.022888533
Furin	5.31E-09	0.160891765	0.000117413
Cbl	7.60E-09	0.16087108	0.000168045

Trpv6	1.36E-09	0.160603309	3.01E-05
Mau2	3.01E-07	0.160549723	0.006642902
Letm1	1.62E-08	0.160512486	0.000357351
Pdlim1	1.82E-07	0.159710362	0.004011575
Tob1	5.72E-08	0.159618339	0.001264267
Lpl	6.56E-07	0.159500657	0.014493985
Tmem50b	1.19E-07	0.159458134	0.002635152
Casz1	2.01E-06	0.159381002	0.04436886
Ccnt1	8.31E-08	0.159372527	0.001835882
Cnot3	1.32E-07	0.159157995	0.002917668
Tst	6.89E-07	0.159089387	0.015219954
Coq8b	8.51E-09	0.159029422	0.00018818
Ubl3	6.71E-07	0.158995011	0.014839114
Heatr1	6.82E-09	0.15897007	0.000150642
Etfb	1.84E-08	0.158506334	0.000406443
Gstt1	3.37E-08	0.158123468	0.000744795
Wdfy1	1.01E-06	0.157411538	0.022346739
Chchd10	4.40E-10	0.157265534	9.72E-06
Bcl9l	5.06E-07	0.157247487	0.01118501
Map2k7	5.57E-09	0.156848981	0.000123185
Jag1	7.62E-08	0.156801918	0.001684316
Cth	3.67E-07	0.156735275	0.008113788
Flcn	9.38E-08	0.156685909	0.002073282
B2m	2.62E-07	0.156550048	0.005796814
Zfp687	6.28E-10	0.156340666	1.39E-05
Fgf1	1.96E-08	0.156247644	0.000432863
Mbp	2.38E-07	0.155688775	0.005268382
Rybp	7.54E-08	0.155403254	0.001667039
Pdlim2	2.06E-06	0.155359147	0.045590455
Fbxl5	2.31E-08	0.155278909	0.000510292
Sfpq	1.16E-07	0.154964681	0.002560063
Bex4	8.51E-12	0.154938021	1.88E-07
Steap2	1.36E-06	0.154697891	0.030007441
Fam129b	4.56E-08	0.154026743	0.001008166
Bmpr1a	3.34E-07	0.153996101	0.007387497
Dym	2.05E-08	0.153894872	0.000453092
Srsf4	1.16E-07	0.153821167	0.002559806
Sema4a	4.81E-10	0.153646921	1.06E-05
Tbc1d2b	3.17E-10	0.153575622	7.01E-06
Chil1	2.04E-09	0.153410426	4.51E-05
Ttc13	2.40E-08	0.153236754	0.000531168
Cnot1	2.71E-07	0.153219519	0.005991317
Rpl4	1.83E-10	0.152773928	4.04E-06
Cdc14a	1.13E-08	0.152763608	0.000248633
Gpalpp1	5.05E-09	0.152518803	0.000111533
Tkt	4.84E-07	0.15245299	0.010703154
Prr14	6.35E-09	0.152284217	0.000140305

Liph	5.62E-08	0.152252366	0.001242261
Ino80d	8.86E-07	0.151960076	0.019577951
Fam20b	5.20E-08	0.151937472	0.001149153
Eif4b	8.45E-08	0.151920489	0.001867122
Mdfic	1.74E-06	0.151797388	0.038429796
Fry	4.11E-09	0.151357182	9.08E-05
Gm47969	7.85E-13	0.151323994	1.74E-08
Pcbp2	7.89E-09	0.151109321	0.000174295
Acot1	3.64E-07	0.150936052	0.008037397
Tnpo3	8.64E-08	0.150883598	0.001910288
H2-T23	4.16E-10	0.150835002	9.19E-06
Cavin1	2.26E-07	0.150608106	0.004989827
Arhgef2	2.67E-07	0.150599663	0.005893709
Oaz2	1.49E-07	0.150013858	0.003289362
Tmem243	1.47E-10	0.149648656	3.26E-06
Cdc42ep4	9.65E-07	0.149574743	0.021327626
Pon3	1.94E-07	0.149520497	0.004287004
Armc8	6.34E-08	0.149502411	0.0014003
Slc35g1	5.29E-08	0.149264351	0.001168296
Smad1	1.69E-08	0.148723397	0.000372397
Tmbim6	1.93E-10	0.148412785	4.26E-06
Csnk1a1	2.62E-08	0.148244457	0.00057915
Esyt2	4.13E-07	0.148176758	0.009119036
Sypl	5.38E-07	0.148142046	0.011881327
Ubqln1	7.19E-07	0.148012811	0.015885317
Epb41l5	2.46E-07	0.148000278	0.005439438
Mgea5	1.18E-07	0.147919109	0.002616985
Kif1c	7.43E-09	0.147595215	0.000164213
Snrnp200	2.37E-07	0.147586064	0.005236885
Taok3	3.28E-09	0.147417902	7.25E-05
Clcn4	1.70E-06	0.14737046	0.037562334
Sptbn1	9.23E-08	0.147203432	0.002040568
Gramd4	5.11E-09	0.147178301	0.000112822
Dlg3	6.59E-08	0.146993985	0.001456204
Gm44751	1.06E-07	0.146935356	0.002333676
Bcl6	1.79E-06	0.1467595	0.039623539
Idh2	2.08E-07	0.146756451	0.004604855
Cbx4	2.79E-07	0.14671817	0.006165187
Antxr1	8.55E-07	0.146683323	0.018898773
Sik1	5.98E-07	0.146565634	0.013219228
Atxn2	1.96E-06	0.146252249	0.043352674
Cyth3	1.44E-08	0.146223944	0.000318197
Inpp5b	1.72E-07	0.146187512	0.003797489
Ccnl1	6.17E-07	0.14616149	0.013630287
Rpl14	1.15E-09	0.146098882	2.54E-05
Mlph	6.79E-08	0.145409072	0.00150061
Scarb2	1.06E-07	0.145008998	0.002344643

Biology Open • Supplementary information

Pip5k1b	1.91E-06	0.145007375	0.042301122
H2-DMb1	1.97E-07	0.144644052	0.004360471
Syt7	7.55E-10	0.14461891	1.67E-05
Sh3rf1	9.77E-07	0.144557949	0.021598201
Akap13	1.56E-06	0.144212585	0.034582052
C1galt1	1.28E-06	0.144199511	0.028316145
Osbp	4.95E-08	0.144115412	0.001093552
Tug1	4.23E-07	0.143988831	0.009340811
Cpsf3	1.03E-06	0.143955863	0.022727857
Eci2	2.41E-07	0.143538958	0.005317992
Ube3a	1.48E-07	0.14351198	0.003277489
Sipa1l3	3.71E-07	0.143486544	0.008205861
Sorl1	1.61E-06	0.143372613	0.03562668
Ubn2	5.63E-07	0.143054439	0.012448893
Aplp2	4.03E-11	0.143030844	8.90E-07
St3gal1	4.12E-08	0.142886434	0.000911613
Dcaf11	5.46E-07	0.142772869	0.012067484
Arcn1	2.05E-06	0.142658727	0.045317472
Brd3	1.69E-07	0.142628339	0.003736936
Ndufb3	9.81E-07	0.142467534	0.021676635
Gak	1.61E-07	0.142301433	0.003548659
Zbtb7a	2.21E-07	0.141963049	0.004888419
Paip2b	1.13E-07	0.141565911	0.002501952
Matr3	2.22E-07	0.141311246	0.004907306
Rgmb	3.50E-09	0.14129698	7.73E-05
Kif13a	1.92E-07	0.141082961	0.004245855
Ehmt2	5.51E-07	0.140860715	0.012185676
Kmt2e	2.13E-07	0.140854346	0.004702601
Rhbdf2	1.68E-07	0.140816849	0.003720986
Snx21	1.10E-06	0.140484289	0.024269337
Nkx2-1	1.93E-10	0.140040065	4.26E-06
Hnmt	3.36E-08	0.139696777	0.000743252
Epb41	1.88E-06	0.139512449	0.041647967
Ick	3.50E-07	0.139485438	0.007745109
Slc16a7	1.49E-06	0.138978557	0.032923603
Epb41l1	1.10E-07	0.138978236	0.002424438
Txndc11	2.29E-07	0.138947311	0.005051438
Ppp1r13b	7.48E-07	0.138759216	0.016526343
Agap3	1.00E-06	0.138593852	0.022141889
Taok1	1.84E-06	0.138446511	0.040637904
Actn4	8.14E-07	0.138441899	0.017991088
4833439L19Rik	1.33E-07	0.138238356	0.002947671
Net1	3.54E-07	0.138050657	0.007827449
Ang	5.91E-07	0.13791538	0.013050189
Ik	5.48E-07	0.137768453	0.012118387
Phc3	2.93E-07	0.137700309	0.006481621
Scamp1	9.22E-08	0.137699115	0.0020382

Biology Open • Supplementary information

Crk	5.68E-08	0.137392257	0.001255401
Epb41l4aos	1.66E-06	0.137379797	0.036646296
Cds2	4.16E-07	0.137231895	0.00918389
Nrn1	1.34E-08	0.136810728	0.000295108
Hspa9	2.16E-06	0.136697487	0.047745804
Stag2	3.84E-07	0.136648558	0.008496568
Socs7	1.55E-09	0.136593166	3.42E-05
Zfp445	6.68E-08	0.136475599	0.001476757
Dopey2	5.94E-07	0.136284602	0.013137973
Galnt13	2.69E-15	0.136267334	5.94E-11
Tnpo2	3.43E-07	0.136193687	0.007582869
Ubr3	1.49E-06	0.136138382	0.032984355
Bscl2	8.97E-07	0.135996281	0.019832791
Pxn	3.23E-07	0.135776721	0.007147056
Grb14	5.39E-07	0.135708766	0.011913
Arid4b	1.35E-06	0.135653744	0.02980489
Wdr81	3.69E-10	0.135453131	8.16E-06
AI464131	2.44E-09	0.135203125	5.39E-05
Tbc1d20	1.91E-06	0.134851808	0.042129709
Elovl5	2.25E-06	0.13480657	0.049722467
Zfp740	1.94E-06	0.134410464	0.042804795
Zfp277	2.18E-06	0.133377038	0.048197052
Gm43672	3.80E-08	0.132968132	0.000839103
Anxa6	1.48E-06	0.13206916	0.032685718
Brpf3	5.86E-07	0.132002447	0.012946048
Cyp39a1	4.15E-07	0.131773826	0.009163456
Slc22a15	6.81E-07	0.131091835	0.015050025
Gnmt	3.46E-09	0.130848127	7.65E-05
Eef1g	6.04E-08	0.130228438	0.001334684
Itpr3	1.88E-06	0.130220782	0.041618413
Gle1	2.19E-06	0.130166748	0.048397197
Ubr5	6.62E-07	0.130063173	0.014640045
Gas2l1	5.17E-07	0.129891806	0.011420282
Pdgfb	4.94E-08	0.129852499	0.001092398
Jade1	1.48E-08	0.129537538	0.000327038
Ppm1b	1.93E-06	0.129442172	0.042683508
Rpe	2.40E-07	0.129335781	0.005294623
Paqr8	1.65E-07	0.129065693	0.003646975
Cog7	3.28E-07	0.128399324	0.007259796
Madd	5.89E-07	0.127414792	0.013026679
Sesn2	1.48E-07	0.127365042	0.00327048
Nr3c1	1.73E-06	0.127346289	0.038216443
Notum	1.46E-09	0.126953679	3.23E-05
Stim1	2.97E-07	0.126559427	0.006557915
Phf1	3.70E-08	0.126375303	0.000818795
Cab39	1.34E-06	0.126286138	0.02960924
Abl1	1.31E-06	0.126143909	0.028996898

Biology Open • Supplementary information

Tmeff1	2.41E-08	0.125691031	0.000531956
Eif4g2	2.93E-08	0.125310221	0.000647112
Hint2	1.13E-06	0.125124679	0.024939297
Vars	1.40E-06	0.12489516	0.030848532
Araf	1.60E-06	0.124860389	0.035417262
Rps14	6.07E-10	0.124771099	1.34E-05
Tmem154	2.04E-09	0.124682823	4.52E-05
Nfe2l2	1.19E-08	0.12418117	0.000262565
Znrf2	4.98E-07	0.123661636	0.011009194
Tab2	1.85E-06	0.123446098	0.040814184
Slc25a20	1.97E-06	0.123038242	0.043480544
Atn1	9.79E-07	0.123024752	0.021644151
Adcy6	4.31E-07	0.122343032	0.009521155
Synm	4.54E-07	0.121542009	0.01003848
Adnp2	6.42E-09	0.121192256	0.000141784
Fbxo31	8.82E-09	0.121012778	0.000194866
Inmt	2.54E-13	0.120548571	5.61E-09
Ppp1r14c	2.38E-07	0.12029389	0.005249333
Eif2ak3	4.39E-08	0.120021148	0.000969556
Rab3d	3.06E-07	0.118882401	0.006760443
Pom121	6.29E-07	0.118747158	0.013898776
9530026P05Rik	3.39E-11	0.118283402	7.49E-07
Atf5	1.26E-06	0.118224703	0.027890612
Mcf2l	1.42E-08	0.118193806	0.000313303
Cdc14b	2.63E-07	0.117583388	0.00581974
Gprc5b	5.97E-07	0.117187661	0.013196698
Ldlrap1	3.43E-07	0.116668217	0.007569252
Lmtk2	2.23E-07	0.116564616	0.004937825
Stard5	6.12E-07	0.116462952	0.013534834
Zkscan17	8.73E-08	0.116448703	0.001929708
Slc4a5	1.15E-06	0.116210823	0.025316273
Slc52a3	6.71E-08	0.116081495	0.001482231
Stard8	2.07E-07	0.116046241	0.004577364
Ncor1	1.71E-06	0.115865717	0.037791983
Sqor	2.71E-08	0.114363192	0.000599658
Mgll	2.23E-07	0.11364097	0.004933135
Slc6a9	1.43E-06	0.113055949	0.031642424
Plbd1	7.93E-11	0.112769772	1.75E-06
Fig4	2.20E-06	0.112050326	0.048544688
Osbpl3	2.89E-08	0.111745747	0.000637976
Rasef	4.51E-08	0.111589703	0.000995902
Rpl3	1.71E-08	0.110541708	0.000378367
Vdr	1.35E-07	0.110366106	0.002985388
Fstl4	5.06E-08	0.108384556	0.001119066
Plcd3	2.69E-08	0.107720968	0.000595555
Tspo	2.09E-07	0.107692156	0.004619626
2610028H24Rik	5.55E-07	0.107304079	0.012254844

Nudt16l1	1.30E-06	0.106702957	0.028632018
Zfp809	9.75E-07	0.106676891	0.021554012
2510009E07Rik	4.80E-08	0.106619693	0.001060734
Vamp8	7.23E-08	0.105918337	0.001596867
Fech	2.50E-07	0.105693594	0.005528426
Ndufa6	4.61E-07	0.104942087	0.010185174
Slc39a9	2.01E-06	0.10459622	0.044462158
Cables1	1.44E-07	0.103048679	0.003183387
Slc35e4	3.40E-09	0.102993963	7.52E-05
Tmem268	2.79E-07	0.101268629	0.00615694
Rack1	6.27E-07	0.099274965	0.013864329
Parp12	5.56E-08	0.098521378	0.001229209
Npdc1	2.68E-08	0.097864247	0.000592442
Mtr	2.65E-07	0.097489453	0.005864223
Paip2	9.60E-07	0.096027294	0.021217588
Etfbkmt	5.01E-07	0.095565758	0.011080022
Aass	6.33E-08	0.09534825	0.001398179
Gm15564	8.12E-08	0.094964303	0.00179441
Rnf125	4.98E-08	0.092096309	0.001099679
Ceacam1	5.62E-07	0.091350552	0.012421692
Ephx1	1.98E-07	0.088487711	0.004372343
Slc7a2	3.67E-07	0.087634735	0.008116794
Prob1	2.49E-08	0.086390988	0.00055065
Vps33b	6.64E-07	0.085783943	0.014667458
Mesp1	6.65E-08	0.083970796	0.001469514
Mlxipl	1.95E-08	0.083859505	0.000430997
Slc19a3	2.82E-07	0.073618976	0.006241634
C130074G19Rik	2.99E-07	0.066529135	0.006603103
Bcan	1.13E-08	0.065609654	0.000249749
Spp1	9.34E-07	0.064382364	0.020647467
Heg1	1.05E-06	0.064179555	0.02323959
Lgals3bp	1.30E-06	0.062726087	0.028708551
Klhl33	1.78E-06	0.046649225	0.039301864
Scgb1a1	3.90E-11	0.004544728	8.61E-07
Bmpr1b	9.08E-07	-0.072104521	0.020065642
Cldn6	7.00E-07	-0.073727579	0.015460126
Fabp3	5.35E-07	-0.104000175	0.011827055
Sftpc	2.47E-14	-0.121252949	5.45E-10
Hmga2	3.21E-10	-0.128674854	7.09E-06
Cldn4	2.32E-11	-0.140589318	5.14E-07
Igf2bp1	3.76E-11	-0.140995327	8.32E-07
Lipa	4.86E-11	-0.14437445	1.07E-06
Ly6i	1.66E-06	-0.146024634	0.036658983
Rpl18	7.86E-07	-0.155337194	0.017366688
Rpl37a	1.18E-09	-0.156085406	2.61E-05
Tpt1	2.51E-09	-0.156273803	5.54E-05
Rpl13	6.46E-10	-0.159131678	1.43E-05

Biology Open • Supplementary information

Cybrd1	1.67E-06	-0.161519572	0.036919061
Rps16	1.40E-08	-0.163425915	0.000310319
Fbn2	4.39E-07	-0.173208522	0.009706679
Aqp1	1.93E-07	-0.17966613	0.004260712
Pbld2	4.93E-07	-0.18287422	0.010885533
Msln	2.14E-07	-0.182900119	0.004731788
Rpl8	4.74E-08	-0.182993682	0.001047746
Slc22a19	3.53E-13	-0.183508822	7.81E-09
Rps10	3.12E-09	-0.184208238	6.89E-05
Col18a1	2.01E-09	-0.184303001	4.43E-05
Rpl19	1.09E-09	-0.184884104	2.40E-05
Gif	4.64E-14	-0.187036625	1.03E-09
Tmem171	3.95E-08	-0.187071609	0.000873637
Rpl34	1.42E-06	-0.188119632	0.03136057
Gm13056	7.59E-13	-0.188295298	1.68E-08
Fau	3.50E-09	-0.189324616	7.73E-05
Rps13	4.42E-07	-0.192798777	0.009776299
Rpl6	6.27E-09	-0.199693616	0.000138637
Lrrc73	1.17E-11	-0.199959812	2.59E-07
St6galnac4	1.39E-06	-0.202369539	0.030684626
Gm17056	2.46E-12	-0.203925497	5.43E-08
Hsd17b12	3.74E-08	-0.207221587	0.000826213
Tspan3	4.90E-08	-0.208756679	0.001082029
Fads3	1.91E-06	-0.210848172	0.042173427
Maged2	1.88E-07	-0.211471528	0.004163799
Ano1	2.71E-08	-0.213717607	0.000598122
S100a14	1.83E-11	-0.213884981	4.03E-07
Rps24	3.35E-12	-0.21461882	7.39E-08
S100a9	1.23E-08	-0.218332275	0.000272787
Nudt18	3.73E-08	-0.220149833	0.0008253
Tmsb4x	4.08E-18	-0.220945652	9.01E-14
Actg1	5.80E-08	-0.221158514	0.001281686
Muc4	8.13E-11	-0.223094042	1.80E-06
Rpl37	5.42E-11	-0.22425717	1.20E-06
Gipc1	2.42E-11	-0.228696021	5.35E-07
Gnas	5.84E-10	-0.229864598	1.29E-05
Cyp51	3.07E-07	-0.230769266	0.006776446
Erg28	1.73E-06	-0.232784017	0.038193733
Rps15a	4.67E-10	-0.236073928	1.03E-05
Igf2bp3	2.03E-13	-0.236633678	4.49E-09
Polr2j	6.97E-07	-0.243374226	0.015411329
Zmat4	1.31E-12	-0.244183209	2.90E-08
Mien1	1.80E-06	-0.248119137	0.039816854
Rpl23	3.83E-20	-0.248460515	8.47E-16
Hcfc1r1	8.98E-07	-0.248703633	0.019848067
Psmb2	7.72E-07	-0.249206216	0.017055744
Rpl28	4.47E-13	-0.250154172	9.89E-09

Ndufa1	8.21E-07	-0.250911063	0.018145066
Aqp3	7.72E-20	-0.254741918	1.71E-15
Cfl2	1.29E-07	-0.255474825	0.002858825
Tacstd2	6.55E-11	-0.255891716	1.45E-06
Frat2	2.50E-07	-0.25717348	0.005514094
Sox9	5.42E-08	-0.25718727	0.001197318
Kcnk2	5.42E-14	-0.257267954	1.20E-09
Rpl10a	1.10E-14	-0.257658895	2.42E-10
Rnaseh2c	3.76E-08	-0.258212522	0.000830319
Hmgn1	1.21E-06	-0.261343894	0.026669747
Ddah2	7.60E-07	-0.261628891	0.01678759
Rpl31	1.34E-10	-0.261828295	2.96E-06
Cox8a	4.24E-11	-0.262837345	9.37E-07
Uqcc2	2.95E-08	-0.263948227	0.00065266
2200002D01Rik	3.22E-13	-0.264530092	7.11E-09
Clu	2.89E-08	-0.265177212	0.000639444
Cox7c	1.13E-08	-0.265832273	0.000249195
Lgals3	1.04E-06	-0.266036105	0.022911267
AU021092	9.43E-08	-0.266862263	0.002084417
Rer1	1.89E-06	-0.269520579	0.041817106
Rpl24	1.16E-15	-0.270771045	2.57E-11
Hacd1	9.53E-10	-0.271298946	2.11E-05
Egfl6	1.92E-07	-0.271517449	0.00424953
Txnl1	1.66E-08	-0.271690621	0.000366609
Edf1	3.55E-08	-0.272640847	0.000784276
Rtn4	1.27E-07	-0.273482595	0.002810526
Gm20186	4.13E-13	-0.275074505	9.12E-09
Sp5	2.51E-09	-0.275370579	5.55E-05
Tpm1	1.43E-08	-0.276144273	0.000315746
Rhoa	1.63E-06	-0.277084901	0.036101317
Fzd2	7.03E-13	-0.27776279	1.55E-08
Myl6	1.32E-08	-0.279273177	0.000290886
Rps11	4.37E-20	-0.280919433	9.65E-16
Btg2	2.95E-07	-0.283196275	0.006519063
Rpl41	1.08E-19	-0.283232663	2.39E-15
Prdx2	8.39E-09	-0.283383551	0.000185392
Tubb2a	6.11E-07	-0.284904592	0.013495858
Rps7	9.61E-13	-0.28555537	2.12E-08
Atpif1	1.58E-08	-0.286271422	0.000349951
Gpc3	2.21E-11	-0.287329913	4.89E-07
Ube2m	1.48E-10	-0.290146667	3.27E-06
Arf4	6.90E-08	-0.291062532	0.001525332
Rac3	1.11E-09	-0.293005086	2.45E-05
Psmd8	1.52E-09	-0.294291114	3.36E-05
Atp5g1	2.90E-08	-0.295608136	0.000641207
Thbs3	2.04E-08	-0.295904622	0.000451866
Uqcrb	6.89E-09	-0.297461286	0.000152165

Biology Open • Supplementary information

Sem1	1.91E-09	-0.298257609	4.22E-05
Asah1	3.47E-08	-0.299863479	0.000767499
Dctn2	3.19E-09	-0.302279923	7.05E-05
Tuba1a	1.16E-14	-0.302454893	2.57E-10
Ftl1	2.42E-20	-0.303404433	5.35E-16
Epha4	3.61E-10	-0.304432129	7.97E-06
Ccnd3	8.31E-10	-0.308794947	1.84E-05
Krt7	9.76E-08	-0.309330457	0.002156539
Rpl32	8.27E-28	-0.310870185	1.83E-23
Peg3	3.82E-15	-0.310952068	8.43E-11
Tmem256	8.35E-10	-0.313375505	1.85E-05
Mrpl23	6.84E-08	-0.31362717	0.001511963
Serpinh1	5.07E-10	-0.315651408	1.12E-05
Clic1	5.42E-10	-0.316051099	1.20E-05
Rab9	5.81E-07	-0.317785494	0.012832564
Krtcap2	6.11E-08	-0.318827253	0.001351014
1110008P14Rik	1.88E-10	-0.319031875	4.16E-06
Dap	8.81E-10	-0.32012472	1.95E-05
Nnat	4.80E-11	-0.321565513	1.06E-06
Rgs10	1.20E-09	-0.323106478	2.66E-05
Clic3	3.52E-07	-0.32415393	0.007782847
Tspan1	2.13E-12	-0.326475605	4.71E-08
Tceal9	3.69E-15	-0.328437119	8.16E-11
Sumo2	8.69E-08	-0.328676565	0.001920962
Cox7b	8.87E-11	-0.329923448	1.96E-06
Pdia6	3.67E-08	-0.331134144	0.000812168
Ube2a	2.35E-10	-0.332459483	5.20E-06
Eif5a	1.06E-11	-0.332769397	2.34E-07
H3f3a	2.88E-19	-0.332883862	6.37E-15
Cyba	3.10E-08	-0.333046266	0.000685929
Tmem213	1.01E-09	-0.333086207	2.24E-05
Tnfrsf12a	4.51E-10	-0.335058732	9.97E-06
Rpl39	1.48E-24	-0.338728417	3.26E-20
Cldn3	8.15E-15	-0.33988806	1.80E-10
Atp5e	8.34E-13	-0.340069102	1.84E-08
Hras	1.36E-11	-0.341114449	3.01E-07
Crip2	6.80E-09	-0.343570877	0.000150281
H3f3b	1.38E-32	-0.344356776	3.05E-28
Atox1	3.62E-12	-0.34682614	8.00E-08
Tagln2	2.33E-10	-0.347993623	5.16E-06
Rpl29	9.06E-15	-0.350968169	2.00E-10
Selenof	2.43E-10	-0.351188556	5.38E-06
Fstl1	4.22E-21	-0.354099177	9.32E-17
Ier3	2.87E-07	-0.365142116	0.00635269
Atp5j2	1.30E-13	-0.365832709	2.87E-09
Tsc22d1	8.13E-13	-0.366689374	1.80E-08
Fbln5	8.44E-14	-0.369345985	1.87E-09

Biology Open • Supplementary information

Matn4	4.99E-11	-0.373396335	1.10E-06
Id1	1.25E-08	-0.373460784	0.000277146
Aes	2.42E-13	-0.373689406	5.36E-09
Ager	1.38E-10	-0.374020435	3.05E-06
Ndnf	1.18E-08	-0.375308907	0.00026138
Hopx	1.05E-06	-0.375441102	0.023151235
Stbd1	2.14E-14	-0.381402994	4.74E-10
Cldn10	3.76E-26	-0.381443682	8.31E-22
Pla2g1b	7.23E-15	-0.381665383	1.60E-10
Tspan8	4.86E-11	-0.383496003	1.07E-06
Ccnd1	8.79E-14	-0.385522481	1.94E-09
Cyr61	6.82E-07	-0.386036708	0.015082724
Rps26	1.84E-28	-0.39032253	4.07E-24
Cstb	9.55E-13	-0.391693972	2.11E-08
Ppia	2.18E-25	-0.39529168	4.81E-21
Bsg	4.93E-17	-0.399213479	1.09E-12
Serpinb6b	1.94E-10	-0.399476007	4.29E-06
Rps20	4.97E-44	-0.400391386	1.10E-39
Napsa	2.63E-37	-0.400834324	5.82E-33
Sfta2	4.48E-19	-0.401008169	9.90E-15
Epcam	7.69E-15	-0.401776991	1.70E-10
Cldn7	6.97E-16	-0.403302544	1.54E-11
Dynll1	2.19E-17	-0.407291598	4.83E-13
Rpl15	1.70E-29	-0.410838304	3.75E-25
Col6a2	1.15E-22	-0.416212815	2.54E-18
Elob	1.77E-17	-0.418890971	3.91E-13
Cxcl15	1.90E-28	-0.419937134	4.20E-24
Ppib	1.09E-19	-0.420560247	2.42E-15
Hsp90ab1	8.66E-25	-0.429342837	1.91E-20
Pfn1	4.06E-18	-0.432930137	8.98E-14
Anxa3	2.99E-11	-0.435285603	6.61E-07
Rplp0	1.13E-48	-0.44138654	2.50E-44
Bex3	2.74E-14	-0.442401216	6.05E-10
Ptma	6.90E-49	-0.447666658	1.52E-44
Tubb4b	2.58E-15	-0.457705612	5.70E-11
Anxa1	9.68E-19	-0.459773468	2.14E-14
Pnrc1	2.69E-18	-0.467851724	5.93E-14
Crlf1	9.52E-20	-0.468334853	2.10E-15
H2afj	7.56E-25	-0.46866248	1.67E-20
Rpl22l1	2.81E-32	-0.483944485	6.22E-28
Ppp1r14b	6.45E-24	-0.490157671	1.43E-19
Cldn18	7.72E-27	-0.495566152	1.71E-22
Serf2	5.27E-29	-0.511489181	1.17E-24
Cox6c	5.21E-40	-0.512335663	1.15E-35
Ubb	1.57E-49	-0.518277741	3.47E-45
Ppic	6.46E-29	-0.539873953	1.43E-24
Tubb5	4.79E-27	-0.548468045	1.06E-22

Biology Open • Supplementary information

Col6a1	2.33E-19	-0.557091906	5.16E-15
Cdkn1c	1.39E-38	-0.559333078	3.06E-34
Nfkbia	3.92E-18	-0.559722791	8.66E-14
AC149090.1	1.20E-15	-0.578571559	2.66E-11
Gng5	3.69E-54	-0.645773588	8.16E-50
S100a6	1.00E-09	-0.722581105	2.21E-05
Tmsb10	1.39E-45	-0.740541392	3.07E-41
Lyz1	6.18E-20	-0.803559134	1.37E-15
Ctsh	4.52E-59	-0.868263404	9.99E-55
Calm1	8.98E-80	-0.871015518	1.98E-75
Areg	4.94E-31	-0.898507072	1.09E-26
Meg3	9.09E-86	-1.025725601	2.01E-81
Dlk1	1.44E-104	-1.589813881	3.19E-100

Table S2.

Biological Processes	PValue	Fold Enrichment	Genes
cytoplasmic translation	2.07927E-22	7.880361223	RPL4, RPL5, RPL3, RPL32, RPL31, RPL34, RPLP0, RPL8, RPL10A, RPL6, RPS14, RPS15A, RPS16, RPL14, RPL13, RPL15, RPS11, RPL18, RPS10, RPL17, RPL39, RPS13, RPL19, RPL41, RPS7, RPL23,
lipid metabolic process	1.35157E-15	2.496834683	DGKD, ACAA2, LPCAT1, AKR1B3, VLDLR, COMT, LIPA, FADS3, HINT2, SPTLC1, LIPH, SCD2, HMGCS2, SCD1, PIP4K2C, LDLRAP1, FADS1, SFTPB, SREBF1, CPT1A, ACOT7, IAH1, PCYT1A, ELOVL5, ACSL5, ACSL4, SREBF2, BSCL2, CYP39A1, ACLY, NPC1, ACOX1, AGPS, ACOT1, PLBD1, CRK, ST6GALNAC4, MGLL, CDS1, ASAH1, HACD1, PRKAA2, PLA2G1B, ABHD2, ATP5A1, GDPD1, LPL, HSD17B12, HACD3, ADIPOR2, ACACA, PTGS1, MTM1, ACAT1, SULT1A1, INPP5B, C3, SCAP, PIP5K1B, B4GALNT1, APOE, PLCG1, ACSS1, LDLR, ST3GAL1, XBP1, GSTM1, CHKA, MID1IP1,
translation	5.42208E-15	3.484321799	RPL4, EIF4A2, RPL5, RPL3, VARS, RPL32, RPL31, RPL34, RPL10A, RPL8, RPL6, RPS14, RPS16, RACK1, RPL37, RPS11, RPL39, RPS13, EIF5A, RPS7, RPL23, RPS6, EEF1G, LARS2, EEF1D, RPL37A, RPL24, RPL29, RPL28, RPS15A, RPL14, EIF4H, RPL13, RPL15, PAIP2, RPL18, RPL17, EIF4B, RPL19,
positive regulation of transcription from RNA polymerase II promoter	4.03176E-11	1.879529703	FOXA1, EHF, CCNT2, CCNT1, ARID4B, NUCKS1, RORA, UBE3A, FGF1, NR3C1, BACH1, ZMIZ1, AKT2, SOX9, ARID2, GTF2I, DDX17, LMO7, FOXP1, PFN1, ATF5, ATF6, ATF4, CASZ1, KMT2A, EPAS1, PIK3R1, FOXO3, FBLN5, ATXN7, PCBP2, NKX2-1, BCL9L, S100A10, EGR1, SMAD1, CREBBP, XBP1, SMAD4, JAG1, FZD5, VDR, HMGA2, MICAL2, NR1D2, PBX1, NFKBIA, NR4A1, MLXIPL, BMP3, RGCC, GDNF, CCPG1, SP1, MAFG, BMPR1B, NFE2L1, FGFR2, NFE2L2, BMPR1A, ARF4, MESP1, DDX3X, LDB1, CITED2, LITAF, FLCN, MED13, DBP, MCF2L, SUMO2, NFKBIZ, ELOB, JAK2, HRAS, KDM6B,
fatty acid biosynthetic process	2.19832E-08	5.046349835	HACD1, ABCD3, PRKAA2, ELOVL5, PLA2G1B, LPL, HSD17B12, HACD3, ACACA, PTGS1, FADS3,
rhythmic process	4.31523E-08	3.515259173	PRKAA2, KMT2A, UBE3A, RORA, RAI1, DBP, RACK1, NKX2-1, EGR1, USP7, CREBBP, GFPT1, NR1D2, CSNK1E, PER1, KAT2B, SFPQ, PER3, NCOR1, TEF, SP1, KLF9, CRY2, BHLHE40, ID1, SIK1, ATF4
fatty acid metabolic process	9.48949E-08	3.056999314	ASAH1, HACD1, PRKAA2, ACAA2, LPL, HACD3, ADIPOR2, LIPA, ACACA, PTGS1, ACAT1, C3, FADS3, ACOXL, SCD2, CD36, SCD1, FADS1, CPT1A, ACOT7, ELOVL5, ACSL5, ACSL4, ACADSB, PER2, GNPAT,
positive regulation of transcription, DNA-templated	1.04066E-07	1.943395315	CDKN1C, MESP1, EHF, CITED2, RORA, FGF1, AFF1, MED13, CDH1, TMSB4X, SOX9, TRP53INP1, NCOA1, SREBF1, MSL2, FOS, RGMB, KLF15, ARID1B, SREBF2, ETV5, PIAS1, FOXP1, KAT2B, MDFIC, ATF5, ATF4, TRIM56, KMT2E, MEG3, CEBPA, CASZ1, NFIX, INO80D, KMT2A, WBP2, EPAS1, SRF, PDGFB, LRP5, FOXO3, LRP6, PDLIM1, RAI1, ATXN7, SPP1, NKX2-1, ZKSCAN17, APOE, MAP2K7,
endocytosis	6.03097E-07	2.873048363	DGKD, TFRC, FKBP15, USP33, LRRK2, LRP5, LRP2, VLDLR, LRP6, CREG1, STBD1, MICALL1, ABL1, SCARF2, LDLRAP1, LDLR, HRAS, SORT1, TNK2, HIP1R, TBC1D2B, AP2B1, CSNK1E, SCAMP1, SORL1,
cholesterol homeostasis	6.54784E-07	3.905843526	ABCA1, XBP1, LRP5, LPL, RORA, SREBF2, TTC39B, CYP39A1, FABP3, MED13, NCOR1, NPC1, CES1D, ACOX1, TSPO, APOE, SCD1, LDLRAP1, LDLR, NFE2L1
lung development	7.49963E-07	3.444777786	FOXA1, SREBF1, CEBPA, ASAH1, RAB3A, MME, EPAS1, HEG1, ABCA3, FGF1, LIPA, FOXP1, RBP4, SP1, MGP, NKX2-1, CHIL1, GPC3, CTNNB1, HMGCS2, FGFR2, BMPR1A, FOXA2
cholesterol metabolic process	1.59661E-06	3.545049505	ABCA1, SREBF1, CEBPA, PRKAA2, FECH, APLP2, PON1, LRP5, VLDLR, SREBF2, CYP51, NPC1, CES1D, APOC1, CAT, SCAP, HMGCS2, APOE, LDLRAP1, LDLR, NFE2L1
in utero embryonic development	2.20121E-06	2.296230776	MEG3, MT-ND4, CITED2, SRF, UBR3, INPP5B, GNA13, FLCN, EPB41L5, BCL2L11, C6, CDH1, MAN2A1, ZMIZ1, VPS54, MGAT1, PLCG1, SLC25A20, PCNT, SLC34A2, SMAD4, MBNL1, HEG1, TPM1, UBE2A, ATP11A, H13, PRRC2B, MTSS1, MIB1, EPN2, FOXP1, NCOR1, SP1, MAFG, CTNNB1,
actin cytoskeleton organization	3.65131E-06	2.747558894	EPB41, SRF, FLII, PDGFB, PDLIM1, EPB41L5, PDLIM2, POF1B, ABL1, RAC3, TMSB10, SPTBN1, ACTN1, LIMK2, STARD8, COBL, ACTN4, SSH2, CDC42BPA, RHOA, MTSS1, NISCH, DIAPH1, PIK3CA,
intracellular signal transduction	8.67206E-06	2.036246425	DGKD, DDX3X, PRKAA2, TFRC, CAB39, CAB39L, NPR1, NPR2, LRRK2, ARAF, ADCY7, ADCY6, GNA13, SOCS2, AKAP13, NRBP2, ZFP36, DEPTOR, AKT2, MCF2L, MKNK2, RACK1, PLCG1, JAK2, SOCS7, MARK2, TNS1, MAP4K4, PDK1, SMAD4, ARHGEF12, DUSP1, CDC42BPG, HMGA2,
transcription from RNA polymerase II promoter	1.18489E-05	2.4796434	EHF, NFIX, LDB1, EPAS1, SRF, ARID4B, PIK3R1, ABLIM1, ATXN7, NFKBIZ, SOX9, JAK2, POLR2J, BCL9L, GTF2I, SMAD1, SREBF1, XBP1, SMAD4, KLF13, STAT3, ASH1L, FOS, FOXP1, NR4A1, BCL6,
negative regulation of apoptotic process	1.25688E-05	1.810891089	ARF4, BTG2, DDX3X, HSP90AB1, TFRC, CITED2, ARAF, AKR1B3, NR3C1, CLU, SH3RF1, AQP1, LGALS3, AKT2, CTSH, SOX9, JAK2, IER3, EIF5A, TSC22D1, DUSP1, RPS6, PIAS1, POR, CAT, CLDN7, TMBIM6, BIRC6, NUPR1, ATF5, GAS6, PTMA, CD44, PRKAA2, XIAP, LRP2, PIK3R1, CBL, FSTL1,
response to insulin	2.95591E-05	3.83073115	EGR1, SORT1, RPS6, PIK3R1, KLF15, KHK, RBP4, AKT2, SESN2, CAT, CRY2, SCAP, SRSF4, SRSF5,
negative regulation of transcription from RNA polymerase II promoter	3.9498E-05	1.582774964	FOXA1, TXN1, CDKN1C, BTG2, LDB1, CITED2, EHMT2, WWC1, ATN1, ZBTB20, ARID4B, TFCP2L1, NR3C1, BACH1, RPS14, FLCN, CCND3, ZFP36, CCND1, SOX9, CD36, SREBF1, PEG3, RPL23, FOXP4, SREBF2, ETV5, PIAS1, FOXP1, SFPQ, NCOR1, CRY2, SIK1, EZR, MET, ATF4, KANK2, CEBPA, HDAC5, NFIX, EPAS1, FOXO3, ZBTB4, FBLN5, PURB, PURA, NKX2-1, ZBTB7A, HEXIM1, IRX1, CBX7, EGR1, IRX2, XBP1, SMAD4, CREBBP, CBX4, VDR, ZBTB16, HMGA2, IRF2BP2, NR1D2, HIPK1, HOPX, PER2,
liver development	4.64317E-05	3.319966997	MEG3, CEBPA, XBP1, CADM1, CITED2, EPHX1, HP, COBL, ACAT1, PIK3CA, CCND1, MAN2A1, SP1,
response to xenobiotic stimulus	4.77033E-05	2.163812356	HDAC5, HSP90AB1, ABCD3, LPL, ATP1A1, FOXO3, ADIPOR2, MT-ND1, CCND1, CDH1, NFKBIZ, ABL1, TSPO, HMGCS2, CBX7, SMAD1, SREBF1, CPT1A, XBP1, SORD, CYBA, FOS, RHOA, SOD1,
adrenal gland development	4.92478E-05	6.402793494	CDKN1C, SMAD4, CITED2, INSR, TSPO, HMGA2, NR3C1, PBX1, LY6E
cellular response to insulin stimulus	5.27875E-05	3.145231892	ERRFI1, XBP1, INSR, IRS2, GPT, PIK3R1, CFLAR, KAT2B, GCLC, CEACAM1, GRB14, TBC1D4, SP1,
response to progesterone	5.34531E-05	5.533278328	NCOA1, SREBF1, CLDN4, WBP2, ABHD2, TSPO, FOSB, UBE3A, MBP, FOS
mitochondrial ATP synthesis coupled proton transport	5.91646E-05	4.110435329	MT-ND4, MT-ND5, NDUFA6, ATP5A1, NDUFB3, NDUFA1, SDHA, MT-ND2, MT-ND3, ATP5J2, MT-
receptor-mediated endocytosis	6.15255E-05	3.820235996	SCARB2, TFRC, INSR, HIP1R, LRP2, VLDLR, SORL1, GAK, MICALL1, APOE, CD36, LDLRAP1, LDLR,
positive regulation of G1/S transition of mitotic cell cycle	6.17126E-05	4.869284928	KMT2E, ANKRD17, CREBBP, CCND3, DDX3X, RGCC, ANXA1, CCND1, CPSF3, RPL17, EIF4G1
regulation of translation	6.21891E-05	2.788772277	PPP1R15B, DDX3X, EIF2AK3, LARP4B, FOXO3, PUM2, PAIP2B, LARP1, CNOT1, AKT2, MKNK2, CNOT3, IGF2BP1, RACK1, IGF2BP3, PAIP2, S100A9, EIF4G3, EIF4G2, EIF4G1, CPEB4
positive regulation of protein phosphorylation	6.51279E-05	2.263613861	TFRC, LRRK2, PIK3R1, FGF1, AGER, ADIPOR2, C3, FLCN, CCND3, CCND1, MPRIP, AKT2, ABL1, RACK1, SOX9, MAP2K7, HRAS, CD74, XBP1, LIMCH1, INSR, LIMK2, MOB1B, PINK1, GPRC5B,
circadian regulation of gene expression	7.12557E-05	3.768611185	EGR1, KMT2A, GFPT1, RORA, CSNK1E, PER2, PER1, RAI1, PER3, NCOR1, CRY2, BHLHE40, ID1, ATF4
positive regulation of gene expression	7.28472E-05	1.763392306	RPL5, DDX3X, TFRC, CITED2, FGF1, CLU, ACTG1, AKT2, LAMP3, CTSH, KIF1B, SOX9, CD36, HRAS, TRP53INP1, RPS7, RPL23, ANK3, TUG1, ADAM19, CLDN3, EZR, GAS6, MET, CD44, ATF4, RBMS3, CEBPA, PDGFB, PIK3R1, AGER, UBR5, NKX2-1, LDLR, CD74, EGR1, SMAD1, CREBBP, JAG1, VDR,
positive regulation of apoptotic process	7.46507E-05	1.991980198	FOXA1, DDX3X, FOXO3, AGER, CLU, PTPRF, LRP6, BCLAF1, BCL2L11, RASSF2, C6, ABL1, RACK1, TSPO, JAK2, TRP53INP1, EIF5A, NCOA1, USP7, ANXA1, TNFRSF12A, DUSP1, ZBTB16, RPS6, OSGIN1, HIP1R, HMGA2, CFLAR, RHOB, NET1, LATS1, NR4A1, RYBP, BCL6, CTNNB1, SPRY1,
aging	0.000106233	2.608545497	CDKN1C, SLC12A2, SREBF1, SMAD4, MT-ND4, TFRC, MT-CO1, AMFR, ATP5A1, GSTT1, FOS, FOXO3, SOD1, MT-ATP6, GCLC, GSTA3, CAT, CANX, TSPO, SCAP, APOE, NFE2L2
response to fatty acid	0.000127476	6.639933993	GNPAT, PON1, CAT, SCD2, SCD1, SOX9, CD36, FOXO3
adipose tissue development	0.000132505	4.471792281	BSCL2, XBP1, PAXIP1, PIK3CA, UBB, LRP5, HMGA2, HMGCS2, HRAS, PUM2, ACAT1
lipid catabolic process	0.000138589	2.915092973	IAH1, PLA2G1B, ABHD2, LPL, DDHD1, PRDX6, LIPA, BSCL2, CYP39A1, HINT2, NCEH1, CES1D, LIPH,
positive regulation of transforming growth factor beta receptor signaling pathway	0.00013939	5.602444307	CDKN1C, FLCN, CREBBP, SMAD4, LRG1, HSP90AB1, CITED2, GIPC1, FURIN

protein stabilization	0.000157011	2.414521452	RPL5, EPHA4, CD74, USP7, COG7, HSP90AB1, RPS7, USP33, RPL23, HIP1R, PIK3R1, ATP1B1, CLU, RTN4, PINK1, PER3, RASSF2, NCOR1, WDR81, CHP1, PFN1, PPIB, NOP53, TSPAN1
regulation of cell proliferation	0.000158035	2.360166111	BEX4, CEBPA, NR3C1, CLU, LRP6, PTGS1, PURA, CCND3, ABL1, SOX9, HRAS, SMAD4, JAG1, ANXA1, STAT3, OSGIN1, PBX1, PIAS1, NFKBIA, BCL6, MAFG, CTNNB1, BIRC6, PLCD3, FGFR2
positive regulation of cell migration	0.000202244	2.155500961	COL18A1, ATP8A1, PDGFB, IRS2, FURIN, PIK3R1, FGF1, AGER, SYNE2, ACTG1, AQP1, MIEN1, AKT2, RACK1, CTSH, JAK2, HRAS, MAP4K4, EPHA4, SEMA4A, XBP1, INSR, STAT3, ACTN4, SSH2, PTP4A1,
stress granule assembly	0.000221874	6.12916984	ATXN2L, DYNC1H1, DDX6, DAZAP2, ATXN2, DDX3X, CSDE1, PUM2
regulation of blood pressure	0.000237428	3.359966599	ENPEP, ARHGEF12, NPR1, LRP5, ECE1, LNPEP, ATP1A1, SOD1, PTGS1, NISCH, GNA13, SULT1A1,
heart development	0.000264967	2.058379538	HDAC5, CITED2, SRF, PDGFB, ECE1, LRP2, ADIPOR2, PDLIM1, AKAP13, PDLIM2, SOX9, KDM6B, HEXIM1, HEG1, MICAL2, AP2B1, FOXP4, HOPX, BICC1, MIB1, FOXP1, ADAM19, KAT2B, RBP4,
response to oxidative stress	0.000270538	2.665325617	PPP1R15B, USP25, NDUFA6, MT-CO1, EPAS1, LRRK2, PRDX6, MT-ND3, ATOX1, SOD1, PTGS1,
apoptotic process	0.000278987	1.662502441	DDX3X, DYRK2, PPP1R13B, JADE1, ECE1, HINT2, AKT2, RACK1, CTSH, SOX9, HRAS, TRP53INP1, PDCD6IP, TNFRSF12A, PEG3, TRAF1, DYNLL1, RHOB, NISCH, MADD, CHIL1, CDIP1, TMBIM6, BIRC6, GAS6, PPIA, S100A9, KANK2, BEX3, CHIA1, ATP5A1, XIAP, FOXO3, GRAMD4, BCLAF1,
respiratory electron transport chain	0.000286654	5.90216355	PINK1, MT-CO1, VPS54, MT-CYTB, ETFB, MT-CO3, SDHA, PUM2
electron transport chain	0.000288363	9.19375476	POR, MT-CO1, AOX3, NDUFB3, MT-CO2, SDHA
skeletal muscle tissue development	0.000293146	3.499424672	MEG3, MBNL1, NFIX, CCNT2, ELN, CFLAR, RHOA, FOXP1, MYL6, CFL2, MYH14, VPS54, MET
positive regulation of neuron apoptotic process	0.000302347	3.280908561	EGR1, ITGA1, FOXO3, NR3C1, AGER, PTPRF, RHOA, BCL2L11, ABL1, CTNNB1, NUPR1, MAP2K7,
regulation of cell shape	0.000311267	2.553820767	SEMA4A, ANXA1, EPB41, PXN, TPM1, BAIAP2, RHOA, RHOB, GNA13, DIAPH1, MKLN1, BAMBI, CDC42EP4, CDC42EP3, RAC3, MYH14, BRWD1, EZR, CRK, PLXNA4
negative regulation of cell migration	0.000311267	2.553820767	EPHA4, CD74, CLIC4, JAG1, LIMCH1, CITED2, SRF, ABHD2, TPM1, PDGFB, ARID4B, FOXO3, RHOB, NISCH, FLCN, CLDN3, CDH1, NKX2-1, ARID2, TRP53INP1
negative regulation of cell-cell adhesion	0.000331102	6.971930693	EPB41L5, JAG1, CDH1, ABL1, PIK3R1, JAK2, MUC4
neuron differentiation	0.0003614	2.151338614	FOXA1, DDX6, BTG2, CASZ1, NFIX, LDB1, RTN4, NRBP2, TUBA1A, CCND1, ABL1, ADNP2, IRX1, IRX2, JAG1, ACSL4, CFLAR, HIPK1, MIB1, GDNF, ID1, CTNNB1, MET, PPIA, EIF4G1, ATF4, FOXA2
growth hormone receptor signaling pathway	0.000374201	12.44987624	SOCS2, STAT3, PXN, PIK3R1, JAK2
positive regulation of pri-miRNA transcription from RNA polymerase II promoter	0.000378034	3.407334549	SMAD1, SREBF1, EGR1, SMAD4, SRF, STAT3, PDGFB, FOS, FOXO3, NR3C1, SREBF2, KLF5, BMPR1A
cellular response to oxidative stress	0.000381823	3.205485376	XBP1, PRKAA2, LRRK2, FOXO3, ETV5, PRDX2, PINK1, SELENOS, SESN2, ADNP2, PPIA, NFE2L1,
cell proliferation	0.000410676	1.929553468	CEBPA, ENPEP, CITED2, IRS2, LRP2, LIPA, PURB, PURA, LARP1, CCND1, SOX9, GLUL, PDK1, CD74, SMAD1, SMAD4, CREBBP, H3F3B, ZBTB16, H3F3A, STAT3, HMGA2, HIPK1, SORL1, PBX1, BCL6,
positive regulation of angiogenesis	0.000429745	2.489975248	XBP1, ANXA3, PXN, STAT3, HMGA2, FGF1, RTN4, HK2, RHOB, AQP1, C3, LGALS3, LRG1, C6,
response to endoplasmic reticulum stress	0.000533955	3.098635864	PPP1R15B, XBP1, SEL1L, EIF2AK3, PIK3R1, PDIA6, HERPUD1, BCL2L11, UBQLN1, ABL1, TMBIM6,
negative regulation of cell proliferation	0.000588312	1.798315457	KANK2, MEG3, CEBPA, BTG2, KMT2A, SRF, ZFP503, TOB2, TOB1, PTPRF, FLCN, CLMN, GPC3, SOX9, JAK2, ARID2, HRAS, TRP53INP1, SMAD1, SMAD4, KLF13, FZD5, VDR, DUSP1, ZBTB16,
bicellular tight junction assembly	0.000602578	3.464313388	CLDN6, CLDN10, CLDN4, CLDN3, PDCD6IP, POF1B, SLC39A9, CDH1, SRF, CLDN7, CLDN18, ACTN4
cell migration	0.000630741	1.954157789	ARF4, ENPEP, USP33, WWC1, PXN, PDGFB, ATN1, IRS2, IQGAP1, TSPAN11, PTPRF, AQP1, PIK3C2B, FUT8, GPC3, IGFBP6, PLCG1, PDK1, CDC42BPA, SORL1, RHOA, RHOB, GOLPH3, PIK3CA,
lipid homeostasis	0.000643589	3.713861386	CEBPA, ACOXL, DDX3X, ACOX1, ZBTB20, NR1D2, APOE, SCD1, ACACA, NFE2L1, PNPLA2
cellular response to epidermal growth factor stimulus	0.000649588	4.06526571	ERRFI1, ZFP36, ID1, PLCG1, IQGAP1, CFLAR, SOX9, BAIAP2, ZFP36L2, PDK1
Rho protein signal transduction	0.000738148	3.651963696	GNA13, NFIX, ARHGEF12, BCL6, MCF2L, CDC42EP4, CDC42EP3, HACD3, BAIAP2, CTNNAL1, RHOA
trophectodermal cell differentiation	0.000757843	6.062548429	CNOT1, CITED2, CDH1, SP1, SRF, CNOT3, HOPX
calcium-independent cell-cell adhesion via plasma membrane cell-adhesion molecules	0.000757843	6.062548429	CLDN6, CLDN10, CLDN4, CLDN3, CADM1, CLDN7, CLDN18
response to hormone	0.000843852	3.592095439	GCLC, POR, NPR2, SRF, SORD, LNPEP, ANG, STEAP2, MT-ND3, SREBF2, ACAT1
lipid biosynthetic process	0.000967965	5.809942244	ACLY, SREBF1, PRKAA2, AGPS, ACSL5, ACSL4, SCD1
negative regulation of cysteine-type endopeptidase activity involved in apoptotic process	0.00097991	3.274487997	NR4A1, POR, DDX3X, AKT2, LAMP3, XIAP, CFLAR, GAS6, SH3RF1, CD44, HERPUD1, AQP1
endoplasmic reticulum unfolded protein response	0.001016917	3.83073115	XBP1, SELENOS, CCND1, AMFR, CREB3L1, EIF2AK3, ATF6, HERPUD1, ATF4, NFE2L2
acetyl-CoA metabolic process	0.001035323	9.95990099	ACLY, ACAA2, FASN, HMGCS2, ACACA
response to toxic substance	0.001061245	3.04655795	PON3, PON1, SLC6A14, EPHX1, FOS, INMT, CES1D, CDH1, MBP, MT-CYTB, NUPR1, TRPM6, ATF4
fat cell differentiation	0.001097106	2.875022966	SREBF1, CEBPA, EIF2AK3, HMGA2, LRP6, PIAS1, BSCL2, NR4A1, CCND1, ADGRF5, ATF5, ZBTB7A,
positive regulation of JNK cascade	0.001097776	2.741257153	DIXDC1, XIAP, TRAF1, AGER, SH3RF1, PJA2, CRACR2A, RASSF2, TAOK3, TAOK1, LTBR, MAP2K7,
glutathione metabolic process	0.001099821	3.230238159	EEF1G, GCLC, GSTM1, CLIC3, GSTP3, GSTA3, CTH, GSTT3, GSTT1, CLIC1, NFE2L1, SOD1
protein phosphorylation	0.001139337	1.591136256	SMG1, DYRK2, CCNT2, CCNT1, LRRK2, ARAF, NRBP2, CCND1, AKT2, SOX9, JAK2, PDK1, EPHA4, BRD2, LMTK2, CSNK1E, LATS1, GAK, GPRC5B, SIK1, GAS6, MET, PRKAA2, NPR1, NPR2, PDGFB, PIK3R1, CBL, MKNK2, ABL1, MAP2K7, TRPM6, ST3GAL1, MARK2, MAP4K4, SMAD1, CSNK1A1,
electron transport coupled proton transport	0.001165373	15.93584158	MT-ND4, MT-ND5, MT-CO1, MT-CYTB
antigen processing and presentation of peptide or polysaccharide antigen via MHC class II	0.001165373	15.93584158	H2-EB1, H2-DMB1, H2-AA, H2-AB1
response to copper ion	0.001172188	7.030518346	ATP7B, TFRC, MT-CO1, SORD, MT-CYTB, SOD1
response to glucose	0.001231566	3.187168317	PFKFB2, SREBF1, EGR1, MLXIPL, SELENOS, COL6A2, SESN2, NNAT, LPL, IRS2, GLUL, KHK
response to ethanol	0.001269492	2.591193754	SREBF1, MT-ND4, CPT1A, EHMT2, ATP5A1, CBL, ADIPOR2, SOD1, CLDN3, CCND1, CAT, CLDN7,
positive regulation of mesenchymal cell proliferation	0.001278089	4.687012231	LRP5, CTNNB1, IRS2, SOX9, FGFR2, BMPR1A, FOXP1, LRP6
cellular response to glucose stimulus	0.001305603	2.976522135	XBP1, SMAD4, PRKAA2, SRF, ZBTB20, CYBA, IRS2, FOXO3, MLXIPL, GCLC, PIK3CA, RACK1, GAS6
lactation	0.001378109	4.07450495	SOCS2, ATP7B, CCND1, VDR, RPLP0, EIF2AK3, MT-CO2, XDH, HK2
phosphorylation	0.001420583	1.591038497	SMG1, DGKD, PANK3, DYRK2, LRRK2, ARAF, HK2, KHK, AKT2, PIP4K2C, JAK2, PDK1, EPHA4, LMTK2, CSNK1E, LATS1, GAK, PIK3CA, FAM20B, SIK1, MET, PRPS2, PRKAA2, ATXN7, MKNK2, ABL1, PIP5K1B, TRPM6, MARK2, MAP4K4, CHKA, CSNK1A1, TNK2, INSR, LIMK2, CDC42BPG,
angiogenesis	0.0014536	1.929946213	COL18A1, ENPEP, CLIC4, EPAS1, NDNF, RORA, TSPAN12, FGF1, ACTG1, GNA13, ANPEP, BSG, GLUL, SEMA4A, XBP1, TNFRSF12A, FZD5, C1GALT1, EIF2AK3, VEZF1, RHOB, KLF5, PIK3CA,
regulation of gene expression	0.001508554	1.680477122	FOXA1, PRKAA2, FECH, KMT2A, LRRK2, TMPRSS4, TOB2, TOB1, AFF1, SLC4A5, FUT8, TSPAN8, CDH1, PCBP2, NFKBIZ, IGF2BP1, ADNP2, IGF2BP3, SOX9, APOE, KDM6B, BRD2, PEG3, SORT1, ASH1L, FOS, ATP1B1, TUG1, KLF15, DLK1, PIAS1, TAPBP, FOXP1, NFKBIA, KLF5, PIK3CA, GDNF,
cellular response to fibroblast growth factor stimulus	0.001519613	5.36302361	NR4A1, GCLC, ZFP36, NDNF, IQGAP1, ZFP36L2, CD44
actin filament organization	0.001527667	2.374545931	TMOD1, SRF, ACTN1, TPM1, HIP1R, RHOA, MTSS1, RHOB, DIAPH1, MPRIP, POF1B, TMSB4X, CFL2,
limb morphogenesis	0.001529396	4.553097595	FBN2, NFIA, NPR2, LRP5, VPS54, BMPR1B, PCNT, LRP6
negative regulation of endoplasmic reticulum stress-induced intrinsic apoptotic signaling pathway	0.001556538	6.639933993	XBP1, SELENOS, LRRK2, CREB3L1, TMBIM6, HERPUD1
positive regulation of cholesterol biosynthetic process	0.001562583	9.054455446	SREBF1, POR, SCAP, FGF1, SREBF2
positive regulation of signal transduction by p53 class mediator	0.001562583	9.054455446	HEXIM1, RPL23, RPS20, RPL37

Biology Open • Supplementary information

microtubule cytoskeleton organization	0.001641703	2.358923919	TBCEL, DST, CDC42BPA, TUBB4B, CAMSAP1, CDC14A, CDC14B, TUBB2A, TUBB5, TUBA1A, ATXN7, TAOK1, RANBP10, TACC1, TACC2, PCNT, PHLDB2, MARK2
regulation of circadian rhythm	0.002218114	3.175620606	PER2, PER1, USP7, SFPQ, PRKAA2, CRY2, BHLHE40, NR1D2, UBE3A, RORA, CSNK1E
negative regulation of erythrocyte differentiation	0.002251331	8.299917492	HSPA9, ZFP36, KLF13, LDB1, GAS2L1
mRNA transcription from RNA polymerase II promoter	0.00245894	3.734962871	NCOA1, SREBF1, VDR, SP1, EPAS1, STAT3, NFKBIZ, S100A10, ATF4
aerobic respiration	0.002473954	3.130254597	MT-ND4, MT-ND5, NDUFA6, MT-CO1, CAT, NDUFB3, NDUFA1, MT-CO3, MT-ND2, MT-ND3, MT-
cartilage development	0.002553709	2.754866231	SMAD1, NPR2, ZBTB16, BMP3, BMP1, NFIA, MGP, GNAS, SOX9, BMPR1B, ZBTB7A, CD44,
mitochondrial electron transport, cytochrome c to oxygen	0.002590992	5.975940594	COX8A, MT-CO1, MT-CO3, COX6C, COX7C, COX6A2
immunoglobulin production involved in immunoglobulin mediated immune response	0.002590992	5.975940594	H2-EB1, H2-DMB1, NFKBIZ, H2-AA, B2M, H2-AB1
skeletal system development	0.002767205	2.396367156	CDKN1C, COL18A1, NFIX, VDR, ZBTB16, EIF2AK3, ASH1L, PUM2, BCAN, RAI1, LGALS3, RASSF2,
ureteric bud development	0.002811985	3.658739139	SMAD1, SMAD4, GDNF, EPCAM, GSTA3, CAT, SPRY1, CRLF1, FGFR2
negative regulation of gene expression	0.002962983	1.714615867	HDAC5, PRKAA2, CITED2, ZFP503, PDGFB, ZBTB20, ADIPOR2, FLCN, CREB3L1, RACK1, SOX9, CD36, APOE, GAS2L1, LDLR, XDH, HRAS, TRP53INP1, VDR, INSR, MBD1, STAT3, EIF2AK3, FOXP1, PINK1,
cellular response to starvation	0.003104928	2.845685997	SREBF1, FLCN, EHMT2, USP33, LRRK2, RNASE4, GAS6, GAS2L1, GLUL, SREBF2, FADS1, PIK3C2B
epithelial cell maturation	0.003123762	7.6614623	CEBPA, XBP1, EPAS1, TFCP2L1, AKR1B3
positive regulation of nuclear-transcribed mRNA poly(A) tail shortening	0.003123762	7.6614623	BTG2, ZFP36, CNOT1, PABPC1, TOB1
regulation of fatty acid metabolic process	0.003123762	7.6614623	SREBF1, EIF2AK3, SCAP, ACSL4, NFE2L1
vacuole organization	0.003123762	7.6614623	FIG4, MAN2A1, NPR2, VPS54, B4GALNT1
osteoclast differentiation	0.003203327	3.585564356	DAP, NFIX, TFRC, GPC3, CTNNB1, PIK3R1, FOS, TOB2, FOXP1
positive regulation of endothelial cell apoptotic process	0.003261931	5.691371994	COL18A1, XBP1, RGCC, PLCG1, FOXO3, AGER
cellular response to glucocorticoid stimulus	0.003303126	4.647953795	ZFP36, ANXA1, EPHX1, HMGCS2, NR3C1, MT-ND3, ZFP36L2
positive regulation of cell differentiation	0.003386055	3.001613997	SMAD1, SLC6A6, HSP90AB1, GDNF, SRF, CTNNB1, JAK2, ARID2, BMPR1B, ARID1B, CLU
glucose homeostasis	0.003443859	2.199732734	FBN2, FOXA1, CEBPA, PRKAA2, INSR, RPS6, STAT3, NUCKS1, FOXO3, ADIPOR2, MLXIPL, GPRC5B,
response to hypoxia	0.003481912	2.040565081	EGR1, SMAD4, MT-ND4, MT-ND5, TFRC, CITED2, EPAS1, SRF, ITPR2, CYBA, ECE1, ACTN4, ATP1B1, AGER, HK2, PDLIM1, CLDN3, CAT, SCAP, MT-CYTB, ANG
lipid transport	0.003493201	2.138283828	ABCA1, ABCD3, STARD5, ATP8A1, TMEM41B, OSBPL3, ABCA3, ATP11A, VLDLR, NPC1, APOC1, OSBP, SLCO2A1, TSPO, ESYT2, APOE, CD36, SLC25A20, LDLR
establishment of localization in cell	0.003679933	2.030659425	ABCA1, PON3, RAB3A, SLC31A1, NEDD4L, CYBA, SCAMP1, AQP3, SLC7A2, ARCN1, AQP1, VAMP8, ANO1, PINK1, ATP7B, NPC1, STIM1, NNAT, ABL1, LDLR, MET
negative regulation of neuron apoptotic process	0.003710899	2.074979373	BTG2, NFIX, HSP90AB1, XIAP, NDNF, RHOA, SOD1, NRBP2, PRDX2, GCLC, PINK1, GDNF, PIK3CA,
negative regulation of ERK1 and ERK2 cascade	0.003735864	2.779507253	FLCN, ERRFI1, SPRED2, EPHA4, XBP1, SMAD4, GSTP3, DUSP1, ABL1, SPRY1, EZR, EIF3A
negative regulation of RNA splicing	0.003780468	11.38274399	RPS26, AKT2, TMBIM6, RPS13
hyperosmotic response	0.003780468	11.38274399	SLC12A2, HNMT, AQP1, PDK1
insulin-like growth factor receptor signaling pathway	0.003924929	4.498019802	GIGYF1, PIK3CA, ATXN7, EIF2AK3, PIK3R1, HRAS, PDK1
cellular response to hypoxia	0.004013706	2.390376238	ACAA2, EPAS1, NDNF, RORA, VLDLR, CFLAR, FOXO3, AQP3, RTN4, AQP1, PINK1, RGCC, UBQLN1,
MAPK cascade	0.004020089	2.489975248	SMAD1, LRRK2, NPR2, ARAF, ASH1L, ZFP36L2, ZFP36, TAOK3, TAOK1, CTNNB1, MBP, CD36,
chromatin organization	0.004166641	1.747351051	KMT2E, FOXA1, HDAC5, PHF2, PRKAA2, KMT2A, EHMT2, PHF1, ARID4B, NUCKS1, NR3C1, CECR2, JAK2, ARID2, ZBTB7A, HMGN1, KDM6B, BRD3, CBX7, BRD2, CREBBP, CBX4, BRPF3, SETD1B,
negative regulation of macroautophagy	0.00420123	7.114214993	NRBP2, SMG1, PINK1, NPC1, LRRK2
execution phase of apoptosis	0.00420123	7.114214993	CECR2, NPR2, TAOK1, MADD, NFKBIZ
beta-amyloid clearance	0.00420123	7.114214993	C3, MME, INSR, LRP2, LDLR
peptide antigen assembly with MHC class II protein complex	0.00420123	7.114214993	H2-EB1, H2-DMB1, H2-AA, B2M, H2-AB1
negative regulation of vascular associated smooth muscle cell migration	0.00420123	7.114214993	GNA13, TPM1, PDGFB, RHOA, NFE2L2
negative regulation of autophagosome assembly	0.00420123	7.114214993	PINK1, EHMT2, LRRK2, NUPR1, MTM1
cellular response to peptide	0.00420123	7.114214993	ANO1, KLF5, NPR2, ID1, KLF15
regulation of cell cycle	0.004462866	1.830087814	FOXA1, CDKN1C, HSP90AB1, INO80D, JADE1, XIAP, CCND3, CCND1, ABL1, RACK1, CCNL1, GAS2L1, CLIC1, HRAS, RPRM, RPL23, STAT3, HMGA2, CABLES1, PER2, MLXIPL, SFPQ, RGCC,
positive regulation of epithelial cell migration	0.004525788	3.794247996	EPB41L5, CTSH, IRS2, PLCG1, SOX9, PFN1, RTN4, AQP1
bone development	0.004870059	2.685815997	SMAD1, AKAP13, CADM1, NPR2, GNAS, LRP5, DYM, RPL13, NOTUM, BMPR1B, PPIB, FGFR2
ribosomal large subunit assembly	0.004963637	5.196470082	RPL5, RPL3, RPLP0, RPL24, NOP53, RPL6
circadian rhythm	0.005020491	2.538798292	SREBF1, CSNK1E, PER2, PER1, CLDN4, PER3, DBP, KLF9, CRY2, BHLHE40, ID1, ATF5, ATF4
homeostasis of number of cells within a tissue	0.005176637	3.706009671	NFIX, TUBA1A, KMT2A, NFKBIZ, RAC3, VPS54, SOX9, LIPA
activation of protein kinase B activity	0.00542241	4.225412541	INSR, PDGFB, ANG, FGF1, GAS6, PPIA, PDK1
positive regulation of reactive oxygen species metabolic process	0.00542241	4.225412541	EIF5A, PDGFB, TSPO, CD36, XDH, AGER, NFE2L2
actomyosin structure organization	0.00542241	4.225412541	EPB41L5, LIMCH1, EPB41, EPB41L1, CDC42BPG, MYH14, CDC42BPA
positive regulation of peptidyl-serine phosphorylation	0.005430327	2.514149764	TXN1, HSP90AB1, TFRC, ARAF, LATS1, DAP, PINK1, PIK3CA, AKT2, GAS6, MET, CD44, EIF4G1
positive regulation of proteasomal ubiquitin-dependent protein catabolic process	0.005480421	2.809202843	GCLC, CSNK1A1, LRRK2, SUMO2, RACK1, CSNK1E, ARIH1, CLU, NOP53, SH3RF1, PIAS1
regulation of extrinsic apoptotic signaling pathway	0.005824229	9.95990099	DEPTOR, MADD, ACSL5, TRAF1
positive regulation of sodium ion transport	0.006015129	4.979950495	AKT2, GNAS, NEDD4L, PRSS8, ANK3, MLLT6
epithelial tube branching involved in lung morphogenesis	0.006015129	4.979950495	FOXA1, CTNNB1, HMGA2, SOX9, FGFR2, FOXA2
actin cytoskeleton reorganization	0.006057281	2.973104773	MKLN1, CLDN3, ANXA1, CDH1, CDC42BPG, CDC42BPA, EZR, ANTXR1, S100A9, RHOA
cell redox homeostasis	0.006310479	4.101135702	TXN1, PRDX2, GCLC, SELENOS, PRDX6, NFE2L1, NFE2L2
unsaturated fatty acid biosynthetic process	0.007051065	6.224938119	FADS3, ELOVL5, SCD2, SCD1, FADS1
positive regulation of nuclear-transcribed mRNA catabolic process, deadenylation-dependent decay	0.007051065	6.224938119	ZFP36, CNOT1, PABPC1, TOB1, ZFP36L2
lung epithelial cell differentiation	0.007051065	6.224938119	FOXA1, NUMA1, CTNNB1, SOX9, FOXA2
regulation of growth	0.007153892	2.705158294	SOCS2, CREG1, SELENOP, RACK1, OSGIN1, GPC3, HMGA2, VPS54, GAS6, TKT, SOCS7
antigen processing and presentation of exogenous peptide antigen via MHC class II	0.007213878	4.780752475	CD74, H2-EB1, H2-DMB1, H2-AA, B2M, H2-AB1
neuron fate specification	0.007213878	4.780752475	FOXA1, NFIX, NFIA, EHMT2, SOX9, FOXA2
very-low-density lipoprotein particle clearance	0.007286876	19.91980198	APOC1, VLDLR, APOE
epidermal growth factor receptor signaling pathway	0.007552525	3.464313388	ARF4, NPR2, ABL1, PLCG1, IQGAP1, SOX9, AREG, PDK1
ion transport	0.007846382	1.491499316	ATP6V1A, SLC26A2, CLIC4, CLIC3, ATP5A1, MLC1, ITPR2, ITPR3, ATP1A1, ATP5G1, SLC4A5, CRACR2A, ATP7B, SLC22A15, SLC39A9, ATP5E, SLC22A19, TSPO, TMEM109, TRPM6, CLIC1, ATP6V1C2, SLC12A2, SLC34A2, ATP6V1G1, SLC31A1, SLC30A5, KCNJ15, ATP1B1, ATOX1, ATP5J2,
negative regulation of canonical Wnt signaling pathway	0.007899497	2.213311331	EGR1, CSNK1A1, AMFR, JADE1, FOXO3, NKD1, BICC1, LATS1, CDH1, GPC3, CTNNB1, APOE, SOX9,
response to activity	0.00804991	3.091003756	SULT1A1, GCLC, PRKAA2, PIK3CA, CAB39, CAT, CRY2, CYBA, CBL
steroid metabolic process	0.008412343	2.197036983	SREBF1, PRKAA2, STARD5, VLDLR, SREBF2, CYP51, CYP39A1, SULT1A1, ERG28, NPC1, SCAP,

Biology Open • Supplementary information

reactive oxygen species metabolic process	0.008499445	3.390604592	LRRK2, SESN2, ATPIF1, PDGFB, CYBA, MT-ND2, MET, SOD1
cellular response to fatty acid	0.008569655	4.59687738	SREBF1, CPT1A, LPL, HMGCS2, ADIPOR2, LDLR
cell-matrix adhesion	0.008579774	2.489975248	BCAN, MKLN1, BCL2L11, BCL6, SRF, ITGA1, PXN, CTNNB1, MSLN, MUC4, RHOA, ITGA9
insulin receptor signaling pathway	0.008838158	2.805605913	SREBF1, PIK3CA, GRB14, INSR, AKT2, IRS2, PIK3R1, HRAS, SOCS7, PDK1
endothelial cell morphogenesis	0.00885995	5.858765288	COL18A1, CLIC4, HEG1, ID1, MET
cellular response to fluid shear stress	0.00885995	5.858765288	HDAC5, XBP1, SPP1, MTSS1, NFE2L2
positive regulation of cartilage development	0.00885995	5.858765288	SMAD1, BMP1, ZBTB16, SOX9, BMPR1B
positive regulation of phosphoprotein phosphatase activity	0.00885995	5.858765288	PPP1R15B, HSP90AB1, ITGA1, JAK2, CALM1
negative regulation of glycolytic process	0.00885995	5.858765288	FLCN, NCOR1, STAT3, NUPR1, IER3
fatty acid beta-oxidation	0.008914142	3.038613861	ACOXL, CPT1A, ABCD3, ACAA2, ACOX1, ECI2, SESN2, ACADSB, ACAT1
response to mechanical stimulus	0.008914142	3.038613861	BTG2, TUBA1A, CITED2, CHIL1, FOSB, CD36, PPL, KCNK2, ACTG1
male gonad development	0.008951157	2.181000217	SMAD4, H3F3B, CITED2, INSR, H3F3A, ATN1, HMGA2, LRP2, CBL, RBP4, BCL2L11, UBB, TBC1D20,
transforming growth factor beta receptor signaling pathway	0.009251871	2.4643054	SMAD1, FLCN, FUT8, SMAD4, GDNF, CITED2, ZMIZ1, BAMBI, STAT3, PXN, FOS, BMPR1B
response to cAMP	0.009530751	3.319966997	PER1, SREBF1, BSG, FOSB, HMGCS2, PIK3R1, FOS, AREG
positive regulation of stem cell proliferation	0.009530751	3.319966997	HDAC5, EPCAM, CTNNB1, HMGA2, SOX9, PBX1, FGFR2, LRP6
mitochondrion organization	0.009747653	2.332949781	CEBPA, CLUH, RAB3A, EPAS1, LRRK2, CHCHD10, TUG1, PUM2, PINK1, WDR81, MAN2A1, SESN2,
positive regulation of I-kappaB kinase/NF-kappaB signaling	0.010067315	2.083116547	ANKRD17, CD74, TFRC, CFLAR, LITAF, RHOA, AKAP13, GPRC5B, PINK1, CTH, PELI1, ABL1, CTNNB1,
response to light stimulus	0.010091875	4.426622662	PER1, KMT2A, FECH, CRY2, BHLHE40, FOS
peptidyl-serine phosphorylation	0.010214864	1.970090306	SMG1, DYRK2, CAB39, CSNK1A1, LRRK2, EIF2AK3, PDGFB, LMTK2, CSNK1E, HIPK1, LATS1, PINK1,
cellular response to organic cyclic compound	0.010546969	2.728739997	NFKBIA, SMAD1, CEBPA, KLF5, HSP90AB1, LRRK2, AKT2, STAT3, SP5, CYBA
cellular response to dexamethasone stimulus	0.010650545	3.252212568	ERRFI1, GDNF, RPL32, ATP5A1, CFLAR, JAK2, NR3C1, AQP1
positive regulation of cell cycle	0.010650545	3.252212568	ANKRD17, BCL2L11, RPS15A, CITED2, CCPG1, NUPR1, FGFR2, LRP6
neuron projection morphogenesis	0.010789491	2.547881649	ATXN2, TAOK3, UBB, LRRK2, TAOK1, ITGA1, VPS54, LIFR, CLU, PLXNA4, MAP4K4
positive regulation of intrinsic apoptotic signaling pathway	0.010934588	3.669437207	FLCN, BCLAF1, BCL2L11, RACK1, NUPR1, CLU, S100A9
response to starvation	0.010934588	3.669437207	GNPAT, ZFP36, GPT, HMGCS2, CBL, FOXO3, ACAT1
post-embryonic development	0.011159143	2.291658635	MEG3, RAB3A, KMT2A, HEG1, ATN1, ASH1L, DLK1, BCL2L11, SELENOP, ABL1, GNAS, ATF5, FGFR2
stress-activated protein kinase signaling cascade	0.011574763	7.967920792	ERRFI1, USP25, AKR1B3, RHOA
negative regulation of protein metabolic process	0.011574763	7.967920792	HSP90AB1, PRPF18, APOE, LDLR
regulation of endothelial cell migration	0.011574763	7.967920792	SEMA4A, CEACAM1, GLUL, PDK1
cardiac vascular smooth muscle cell differentiation	0.011574763	7.967920792	MESP1, SRF, PDGFB, CTNNB1
progesterone receptor signaling pathway	0.011574763	7.967920792	WBP2, UBR5, KLF9, UBE3A
mesendoderm development	0.011574763	7.967920792	SMAD4, LDB1, FOXA2, BMPR1A
bone mineralization	0.011923298	2.891584158	NFIX, EIF2AK3, SPP1, GPC3, SOX9, FGFR2, MTSS1, DLK1, ATF4
multicellular organism development	0.012041473	1.323153362	FOXA1, MESP1, LDB1, CITED2, PLEKHB1, DIXDC1, RORA, FGF1, SPRED2, SEC61A1, ZFP36, CDH1, AKT2, ANPEP, CREB3L1, RACK1, PAQR8, EPHA4, USP7, EGFL6, TNFRSF12A, HEG1, EDF1, RHOB, PTP4A1, ANO1, TMEFF1, TET3, SIK1, ANG, ARHGEF2, MET, CEBPA, CASZ1, HACD1, EPAS1, FLII, SRF, PDGFB, LRP5, FSTL1, NKD1, FSTL4, ZFP36L2, LRP6, NNAT, ZBTB7A, MARK2, SEMA4A, XBP1,
endothelial cell migration	0.012388324	3.575349073	PAXIP1, GIPC1, PXN, ABL1, S100A9, FSTL1, RHOA
triglyceride metabolic process	0.012388324	3.575349073	CPT1A, SEL1L, APOC1, CAT, APOE, SCD1, CD36
protein localization to plasma membrane	0.012922522	1.968817638	RAB3A, RAB3D, RHBDF2, ANK3, ATP1B1, ZDHHC3, LRP6, SYTL2, GAK, CDH1, AKT2, SESN2, BSG,
phospholipid biosynthetic process	0.01307513	2.845685997	CDS1, GNPAT, PCYT1A, CHKA, ETNK1, ADGRF5, LPCAT1, ACSL5, CDS2
regulation of gluconeogenesis	0.013325471	5.242053153	RANBP2, USP7, SESN2, NR3C1, GNMT
cellular response to nitric oxide	0.013325471	5.242053153	ATP5A1, MTR, CFLAR, CRK, AQP1
positive regulation of glycogen biosynthetic process	0.013325471	5.242053153	DYRK2, INSR, AKT2, IRS2, CD36
embryonic camera-type eye morphogenesis	0.013325471	5.242053153	FZD5, CITED2, SP1, HIPK1, LRP6
multicellular organism growth	0.013524566	2.074979373	CDKN1C, MEG3, SLC12A2, CLIC4, NFIX, H3F3B, HEG1, NPR2, H3F3A, ATN1, DLK1, GNAS, RPL29,
fibroblast migration	0.013671161	4.121338341	PDLIM1, FUT8, PDGFB, IQGAP1, AQP1, TNS1
entrainment of circadian clock by photoperiod	0.013671161	4.121338341	PER2, PER1, PER3, CRY2, BHLHE40, SIK1
establishment of skin barrier	0.013972551	3.485965347	GAK, CLDN4, NCOR1, CDH1, SRF, NFKBIZ, MET
regulation of hydrogen peroxide metabolic process	0.014089486	14.93985149	PRDX2, PINK1, INSR
receptor-mediated endocytosis involved in cholesterol transport	0.014089486	14.93985149	LDLRAP1, LDLR, LRP6
glial cell fate specification	0.014089486	14.93985149	NFIX, NFIA, SOX9
tangential migration from the subventricular zone to the olfactory	0.014089486	14.93985149	LRRK2, SRF, OGDH
hydrogen ion transmembrane transport	0.014306268	2.801222153	ATP6V1A, ATP6V1G1, MT-CO1, ATP6V1B2, CHP1, ATP1A1, ATP1B1, ATP5G1, ATP6V1C2
cellular response to growth factor stimulus	0.014693263	2.58698727	INSR, CAT, RACK1, PDGFB, CTNNB1, LRP2, BMPR1B, GAS6, FGFR2, BMPR1A
regulation of transcription from RNA polymerase II promoter	0.014880666	1.246530359	FOXA1, EHF, ZFP445, CCNT2, CCNT1, EHMT2, JADE1, ZBTB20, ARID4B, NUCKS1, RORA, NR3C1, BACH1, ZFP36, ZMIZ1, CREB3L1, SOX9, CCNL1, ARID2, HMGN1, DDX17, FOXP4, FOXP1, PIAS1, PFN1, ATF5, ATF6, ATF4, EPAS1, FOXO3, RAI1, ATXN7, NKX2-1, ZFP740, ZBTB7A, EGR1, SMAD1, XBP1, SMAD4, BRPF3, FUS, ZBTB16, IRF2BP2, HOPX, PBX1, NR4A1, MLXIPL, BCL6, SP1, MAFG, BHLHE40, GNAS, SP5, BRWD1, NFE2L1, NFE2L2, ZFP110, MESP1, PHF2, LDB1, CITED2, TFCP2L1, ZFP809, MED13, DBP, HRAS, BRD3, SREBF1, BRD2, KLF13, TSC22D1, PEG3, TSC22D3, VEZF1, FOS,
osteoblast differentiation	0.015157133	2.29843869	HDAC5, SMAD1, BMP3, SMAD4, H3F3B, CREB3L1, H3F3A, SPP1, CTNNB1, BMPR1B, DLK1,
positive regulation of transcription from RNA polymerase II promoter in response to endoplasmic reticulum stress	0.015329331	7.243564356	CREB3L1, EIF2AK3, ATF6, ATF4
SREBP signaling pathway	0.015329331	7.243564356	SREBF1, EIF2AK3, SCAP, SREBF2
sebaceous gland development	0.015329331	7.243564356	CBX7, SMAD4, ASH1L, SCD1
regulation of cellular response to oxidative stress	0.015329331	7.243564356	FUT8, PINK1, MET, NFE2L2
positive regulation of protein catabolic process	0.015679717	2.407888151	LPCAT1, GPC3, NEDD4L, UBR3, SOX9, FBXL5, EZR, SORL1, OAZ2, NKD1, IER3
surfactant homeostasis	0.015744768	3.983960396	NAPSA, EPAS1, ABCA3, ADGRF5, LPCAT1, CTSH
positive regulation of fibroblast proliferation	0.015894938	2.553820767	CD74, ZMIZ1, PLA2G1B, ABL1, PDGFB, HMGA2, GAS6, HRAS, AGER, AQP1
stress fiber assembly	0.016009091	4.979950495	PDLIM1, ELN, SRF, FHDC1, RHOA
positive regulation of beta-amyloid formation	0.016009091	4.979950495	EPHA4, SP1, APOE, CSNK1E, CLU
protein secretion	0.016094678	3.006762563	ABCA1, GOLPH3, RAB3A, CAVIN1, RAB3D, SEL1L, RAB27A, PLCG1
negative regulation of transforming growth factor beta receptor signaling pathway	0.016828332	2.381715454	SPRED2, XBP1, BAMBI, SNX25, NKX2-1, ARID4B, SPRY1, ZBTB7A, PBLD2, BCL9L, PDK1
positive regulation of osteoblast differentiation	0.017167134	2.521493922	FBN2, SMAD1, CEBPA, JAG1, GNAS, LRP5, CTNNB1, BMPR1B, CLIC1, BMPR1A
brown fat cell differentiation	0.017553799	3.319966997	SELENBP1, CEBPA, ALDH6A1, FABP3, LRG1, PEX11A, SCD1
gene expression	0.018000163	1.718571151	DDX17, SCARB2, MESP1, NFIX, FUS, EPAS1, STAT3, LRP5, ECE1, NR3C1, GOLPH3, NCOR1, TUBA1A, NPC1, NFIA, NFKBIZ, NKX2-1, CTNNB1, VPS54, APOE, SOX9, FOXA2
negative regulation of neurogenesis	0.018017814	3.855445545	CTNNB1, ARHGEF2, ATF5, SORL1, B2M, BMPR1A
retina development in camera-type eye	0.018037987	2.356105611	C3, RBP4, NFIX, NFIA, LPCAT1, PDGFB, RPL24, SOX9, BMPR1B, SLC4A5, NECTIN1
negative regulation of translation	0.018413048	2.233996484	DDX6, BTG2, PAIP2B, DDX3X, LARP1, CNOT1, EIF2AK3, IGF2BP1, RACK1, ANG, PAIP2, TOB1

Biology Open • Supplementary information

erythrocyte differentiation	0.018504169	2.675794296	KMT2E, HSPA9, RPS14, FECH, EPAS1, ATPIF1, CFLAR, JAK2, NFE2L1
negative regulation of NF-kappaB transcription factor activity	0.01851204	2.489975248	NFKBIA, PRDX2, USP7, DAP, LRRK2, CAT, ERBIN, PELI1, CHP1, CD200
negative regulation of transcription, DNA-templated	0.018647683	1.395840139	CDKN1C, MEG3, MESP1, HDAC5, CEBPA, LDB1, CITED2, EHMT2, ZFP503, PDGFB, ZBTB20, NR3C1, ZBTB4, PURB, PURA, FLCN, BCLAF1, CREB3L1, SUMO2, NKX2-1, SOX9, ZBTB7A, HEXIM1, CBX7, SMAD4, CBX4, VDR, ZBTB16, HMGA2, NR1D2, PHC3, FOXP1, PER2, PER1, MLXIPL, SFPQ, DAP,
positive regulation of vascular endothelial cell proliferation	0.019008422	4.742809995	COL18A1, SP1, STAT3, PLCG1, PDK1
positive regulation of neuron death	0.019449462	2.897425743	EGR1, PPP1R13B, ABL1, APOE, FOS, CLU, AGER, ATP5G1
positive regulation of fat cell differentiation	0.019449462	2.897425743	CDS1, CEBPA, XBP1, ZFP36, KLF5, ZBTB16, LRP5, LPL
positive regulation of blood vessel endothelial cell migration	0.019560906	3.242758462	COL18A1, SP1, ATP5A1, PDGFB, PLCG1, NFE2L2, PDK1
negative regulation of long-term synaptic potentiation	0.019688664	6.639933993	EPHA4, ABL1, APOE, AGER
cerebral cortex cell migration	0.019688664	6.639933993	NKX2-1, DIXDC1, RHOA, LRP6
pentose-phosphate shunt	0.019688664	6.639933993	PRPS2, H6PD, RPE, TKT
maintenance of permeability of blood-brain barrier	0.019688664	6.639933993	SLC12A2, MBP, TSPAN12, PTGS1
positive regulation of fatty acid beta-oxidation	0.019688664	6.639933993	CPT1A, AKT2, ACSL5, IRS2
astral microtubule organization	0.019688664	6.639933993	NUMA1, LIMK2, TACC2, EZR
cellular response to glucose starvation	0.019931808	2.459234812	XBP1, PRKAA2, CHKA, SESN2, EIF2AK3, SIK1, FOXO3, ATF4, NFE2L2, CPEB4
positive regulation of cell proliferation	0.02003471	1.407207361	COL18A1, PLA2G1B, LRP5, PDGFB, IRS2, CRIP2, FGF1, CLU, AREG, LGALS3, PURA, CCND3, RPS15A, CCND1, EPCAM, AKT2, CTSH, SOX9, JAK2, HRAS, EPHA4, XBP1, TSC22D1, INSR, RPL23, RPS6, STAT3, HMGA2, LIFR, UBE2A, HIPK1, PBX1, MLXIPL, KLF5, NCOR1, GDNF, CCPG1, BAMBI, ACER3,
translational initiation	0.020080907	2.63644438	EIF4A2, DDX3X, LARP1, EIF4H, EIF4G3, EIF3A, EIF4B, EIF4G2, EIF4G1
tissue homeostasis	0.02049717	3.734962871	NFIX, GNAS, CTNNB1, SOX9, AKR1B3, ACACA
protein processing	0.020647419	2.306503387	ADAM19, CPM, ENPEP, BMP1, MME, DDI2, ANPEP, CPD, TMPRSS4, FURIN, ECE1
response to wounding	0.020702887	2.105344925	NFIX, DST, ABHD2, NPR2, TMPRSS4, PDGFB, NEAT1, ZFP36L2, ZFP36, NFIA, GDNF, SRSF5,
placenta development	0.021297726	2.845685997	CDKN1C, ADAM19, FBN2, HSP90AB1, MME, RPS6, BIRC6, MET
regulation of actin cytoskeleton organization	0.021428549	2.429244144	TAOK1, ABL1, HIP1R, DIXDC1, RAC3, IQGAP1, BAIAP2, CRK, RHOA, RHOB
glucose metabolic process	0.021428549	2.429244144	CBR2, NISCH, CPT1A, PDHA1, H6PD, PIK3CA, AKT2, PIK3R1, HK2, PDK1
negative regulation of neuron differentiation	0.021428549	2.429244144	DDX6, CASZ1, JAG1, DIXDC1, FOXO3, PBX1, RTN4, RHOA, FOXA2, MIB1
retrograde vesicle-mediated transport, Golgi to ER	0.021718637	3.169059406	ARF4, GOLPH3, COG7, RER1, KIF1C, USE1, ARCN1
methionine metabolic process	0.022705561	11.95188119	MTHFD1, MTR, GNMT
positive regulation of metallopeptidase activity	0.022705561	11.95188119	CLDN4, CLDN3, ANTXR1
positive regulation of phospholipid biosynthetic process	0.022705561	11.95188119	FABP3, ADGRF5, CHP1
positive regulation of regulated secretory pathway	0.022705561	11.95188119	RAB3A, RAB3D, RAB27A
positive regulation of mitophagy in response to mitochondrial depolarization	0.022705561	11.95188119	PINK1, ATPIF1, HK2
regulation of water loss via skin	0.022705561	11.95188119	CDH1, SRF, SCD1
positive regulation of transcription from RNA polymerase II promoter in response to oxidative stress	0.022705561	11.95188119	SESN2, ATF4, NFE2L2
regulation of removal of superoxide radicals	0.022705561	11.95188119	CD36, FBLN5, NFE2L2
maintenance of protein location in mitochondrion	0.022705561	11.95188119	PINK1, TSPO, HK2
negative regulation of hematopoietic stem cell differentiation	0.022705561	11.95188119	HSPA9, ZFP36, NFE2L2
transsulfuration	0.022705561	11.95188119	TST, MTHFD1, CTH
response to lipopolysaccharide	0.023015585	1.840416487	ERBIN, FOS, ADIPOR2, LITAF, FOXP1, NFKBIA, PRDX2, NR4A1, SCGB1A1, NFKBIZ, PELI1, NKX2-1,
negative regulation of endothelial cell apoptotic process	0.023189091	3.621782178	ID1, ABL1, NDNF, GAS6, NFE2L2, PDK1
negative regulation of epithelial to mesenchymal transition	0.023189091	3.621782178	FOXA1, SPRED2, EPHA4, NKX2-1, SPRY1, FOXA2
positive regulation of cholesterol efflux	0.023189091	3.621782178	NFKBIA, ABCA1, CES1D, ABCA3, PON1, APOE
skeletal muscle cell differentiation	0.023264398	2.795761681	EGR1, NR4A1, BTG2, KLF5, CITED2, NUPR1, FOS, BCL9L
negative regulation of osteoblast differentiation	0.023516518	2.561117397	HDAC5, BAMBI, ID1, NBR1, LRP5, SOX9, TOB1, AREG, DLK1
cell adhesion	0.023771162	1.401884591	LGALS3BP, COL18A1, LDB1, CLSTN1, PXN, MSLN, PTPRF, FBLN5, THBS3, ALCAM, CDH1, BSG, SPP1, ABL1, CD36, EMB, SCARF2, CTNNAL1, MUC4, EPHA4, EGFL6, CADM1, DST, TNFRSF12A, ITGA1, ATP1B1, RGMB, RHOA, RHOB, CLDN6, BCAN, CLDN10, CLDN4, CEACAM1, CLDN3, COL6A2,
negative regulation of extrinsic apoptotic signaling pathway	0.024031373	3.098635864	LGALS3, PRDX2, GCLC, GSTP3, BIRC6, CFLAR, SH3RF1
long-term memory	0.024031373	3.098635864	EGR1, CREBBP, EHMT2, SRF, APOE, LDLR, PJA2
oxidative phosphorylation	0.024658135	6.12916984	COX7B, MT-CO1, CHCHD10, COX6A2
peptidyl-serine dephosphorylation	0.024658135	6.12916984	PPP1R15B, PPTC7, PPP2R1A, DUSP1
fatty acid catabolic process	0.024658135	6.12916984	ACOT7, ACOX1, ECI2, LPIN2
regulation of apoptotic process	0.024840392	1.717224309	EGR1, LRP5, OSGIN1, TRAF1, ACTN4, CFLAR, CLU, PRDX2, PINK1, RASSF2, BCL2L11, RASSF3, MADD, ABL1, CTNNB1, APOE, CCNL1, JAK2, ZBTB7A, NOP53
canonical Wnt signaling pathway	0.024952714	2.134264498	FZD2, FZD5, CDH1, LRP5, GPC3, DIXDC1, CTNNB1, SOX9, FOXO3, FGFR2, BCL9L, LRP6
positive regulation of stress fiber assembly	0.025352661	2.747558894	RGCC, LIMCH1, TPM1, PXN, ABL1, PFN1, RHOA, S100A10
ERK1 and ERK2 cascade	0.025352661	2.747558894	FLCN, SMAD4, ABL1, CTSH, SPRY1, SOX9, ZFP36L2, FGFR2
regulation of autophagy	0.025380437	2.525045321	XBP1, DRAM1, USP33, LRRK2, LAMP3, CISD1, NUPR1, PIP4K2C, TRP53INP1
cellular response to low-density lipoprotein particle stimulus	0.025989302	4.330391735	NPC1, CES1D, CD36, LDLR, SREBF2
regulation of neuron death	0.025989302	4.330391735	UBB, LRRK2, CHP1, APOE, CLU
negative regulation of stress fiber assembly	0.026099197	3.515259173	KANK2, TACSTD2, PIK3R1, PFN1, PHLDB2, MET
actin filament polymerization	0.026099197	3.515259173	DIAPH1, ABL1, COBL, ANG, FHDC1, MTSS1
tricarboxylic acid cycle	0.026099197	3.515259173	PDHA1, IDH2, OGDH, SUCLG1, ACO2, SDHA
positive regulation of cell-substrate adhesion	0.026503201	3.031274214	HACD1, EGFL6, SPP1, PDGFB, NDNF, HSD17B12, JAK2
positive regulation of glucose import	0.026503201	3.031274214	INSR, AKT2, GPC3, IRS2, ADIPOR2, KLF15, NFE2L2
cellular response to hydrogen peroxide	0.027344977	2.489975248	KDM6B, NET1, ANXA1, ABL1, AKR1B3, ZFP277, AQP1, NFE2L2, RHOB
ossification	0.027990809	2.096821261	BMP3, SLC26A2, RASSF2, BMP1, SORT1, SP1, NPR2, MGP, EIF2AK3, SPP1, SOX9, DLK1
response to organic substance	0.02822569	2.316256044	ASAH1, DGKD, HSP90AB1, NFKBIZ, SPP1, AKR1B3, BMPR1B, BMPR1A, NFE2L2, SOD1
positive regulation of axon extension	0.029137897	2.966779018	RPL4, TNFRSF12A, SRF, MYO5B, APOE, ATP5G1, EIF4G2
posttranscriptional regulation of gene expression	0.029232452	3.414823197	PER1, EPB41L5, LARP1, ZC3H7A, MATR3, PUM2
developmental growth	0.029232452	3.414823197	SMAD4, ZMIZ1, SRF, EIF4H, GNAS, BMPR1A
negative regulation of proteasomal ubiquitin-dependent protein catabolic process	0.029232452	3.414823197	USP7, RYBP, HSP90AB1, GIPC1, NOP53, MTM1
cell morphogenesis	0.029605663	2.078588033	NFIX, BCL6, NFIA, VDR, CDH1, CSNK1A1, ARHGEF2, TFCP2L1, FRY, CLU, LIPA, RHOA
positive regulation of tumor necrosis factor production	0.029605663	2.078588033	FZD5, LRRK2, STAT3, ZBTB20, LPL, CYBA, ARHGEF2, PIK3R1, CD36, JAK2, CLU, AGER
regulation of cell growth	0.029905884	2.655973597	SOCS2, XBP1, CEACAM1, EPB41, CLSTN1, JADE1, FBLN5, CD44
histone H3-K4 methylation	0.029984136	4.149958746	PAXIP1, KMT2A, SETD1B, NFKBIZ, ASH1L
positive regulation of hepatocyte proliferation	0.03023729	5.691371994	XBP1, CFLAR, FGF1, RTN4
cytoskeleton organization	0.030310622	1.991980198	DST, EPB41, PXN, MICAL2, CDC42BPA, SIPA1L3, RHOA, CAMSAP1, DIAPH1, ABLIM1, RANBP10,

Biology Open • Supplementary information

Wnt signaling pathway	0.031167101	1.67393294	PRKAA2, FZD2, DDX3X, LDB1, FZD5, CSNK1A1, AMFR, LRP5, DIXDC1, XIAP, CSNK1E, NKD1, LRP6, FRAT2, CCND1, CTNNB1, RAC3, NOTUM, MARK2, CD44
positive regulation of insulin secretion	0.031584958	2.422678619	PFKFB2, RBP4, STIM1, NNAT, GNAS, ACSL4, IRS2, JAK2, GLUL
positive regulation of sequence-specific DNA binding transcription factor activity	0.031907982	1.976774242	FOXA1, HDAC5, CREBBP, FZD2, ANXA3, SRF, LRP5, HMGA2, LRP6, PINK1, RGCC, CTNNB1, JAK2
positive regulation of epithelial to mesenchymal transition	0.031938917	2.904971122	SMAD4, RGCC, LRG1, BAMBI, CTNNB1, AGER, BCL9L
energy homeostasis	0.032137055	2.263613861	MLXIPL, FLCN, PRKAA2, PIK3CA, UBB, STAT3, FMO2, NR1D2, CD36, EIF4G1
vasculature development	0.032593156	3.319966997	FZD5, CITED2, HEG1, LRP5, CTNNB1, PCNT
negative regulation of microtubule depolymerization	0.032593156	3.319966997	ATXN7, TAOK1, MID1IP1, ARHGEF2, GAS2L1, CAMSAP1
protein transport	0.032764482	1.365220073	RAB9, ARF4, POM121, DGKD, RAB3A, RAB3D, MLC1, LRP2, PIK3R1, USE1, MTM1, GLE1, SELENBP1, SEC61A1, SNX27, SNX25, MICALL1, KIF13A, CHP1, VPS54, RAB11FIP2, TNPO2, TNPO3, SEC63, EIF5A, RANBP2, XBP1, SLC15A2, PDCD6IP, COG7, RHBDF2, ARRDC3, VPS33B, SNX21,
positive regulation of hydrogen peroxide-mediated programmed cell death	0.032936455	9.95990099	FOXA1, ABL1, FOXO3
positive regulation of intracellular transport	0.032936455	9.95990099	DYNC1H1, NUMA1, MLC1
pulmonary artery morphogenesis	0.032936455	9.95990099	JAG1, CITED2, LRP2
regulation of modification of synaptic structure	0.032936455	9.95990099	EPHA4, ABL1, RHOA
acylglycerol catabolic process	0.032936455	9.95990099	CES1D, ABHD2, MGLL
mesodermal cell migration	0.032936455	9.95990099	MESP1, LRP5, LRP6
regulation of protein targeting to mitochondrion	0.032936455	9.95990099	SREBF1, PINK1, ATPIF1
copper ion import	0.032936455	9.95990099	ATP7B, SLC31A1, STEAP2
establishment of blood-retinal barrier	0.032936455	9.95990099	LRP5, CTNNB1, LRP6
protein dephosphorylation	0.033683955	1.897123998	DUSP1, DUSP18, SSH2, PTPN13, PTPRF, CDC14A, MTM1, CDC14B, PTP4A1, PPM1B, NCEH1,
epithelial cell proliferation	0.034227145	2.238179998	FLCN, PURA, EHF, CBX4, GPC3, SOX9, FGF1, LIPA, FGFR2, BMPR1A
negative regulation of Rho protein signal transduction	0.034321328	3.983960396	FLCN, BCL6, KCTD10, HEG1, MET
positive regulation of receptor-mediated endocytosis	0.034321328	3.983960396	C3, LDLRAP1, CBL, CLU, B2M
positive regulation of cell motility	0.034321328	3.983960396	CAVIN1, EPCAM, AKT2, CLDN7, CFAP20
protein autophosphorylation	0.035235444	1.745549658	HSPA9, EPHA4, SMG1, LRRK2, INSR, EIF2AK3, LMTK2, TAOK3, TAOK1, MKNK2, ABL1, SIK1, JAK2,
response to antibiotic	0.036184936	3.230238159	CPT1A, ID1, VPS54, DUSP18, CBL, JAK2
cellular response to transforming growth factor beta stimulus	0.036253443	2.358923919	EPB41L5, NFKBIZ, ID1, ABL1, SOX9, NR3C1, CRK, ZFP36L2, FGFR2
negative regulation of epithelial cell proliferation	0.036409009	2.213311331	CDKN1C, FLCN, CDH1, GPC3, NUPR1, SOX9, B2M, FGFR2, MTSS1, LRP6
somite rostral/caudal axis specification	0.036420612	5.311947195	EPB41L5, MESP1, SMAD4, FOXA2
amyloid fibril formation	0.036420612	5.311947195	DAP, FUS, CD36, B2M
positive regulation of viral entry into host cell	0.036420612	5.311947195	CD74, BSG, TMPRSS4, FURIN
cellular response to prostaglandin E stimulus	0.036420612	5.311947195	PRKAA2, GNAS, ACACA, ADCY6
endothelial cell differentiation	0.036420612	5.311947195	KDM6B, MESP1, FZD2, FSTL1
response to selenium ion	0.036420612	5.311947195	MIEN1, SELENOP, RPLP0, GSTT1
negative regulation of protein phosphorylation	0.037923403	2.086836398	PPP1R15B, CADM1, INSR, LRRK2, CTDSP2, CHP1, PTPN13, PPIA, XDH, AGER, LRP6
branching involved in ureteric bud morphogenesis	0.038052093	2.788772277	SMAD4, GDNF, GPC3, CTNNB1, SOX9, PBX1, CD44
gastrulation with mouth forming second	0.039003451	3.83073115	SMAD4, LDB1, LRP5, CTNNB1, LRP6
protein peptidyl-prolyl isomerization	0.039003451	3.83073115	RANBP2, PPIB, PPIA, NKTR, PPIC
mitochondrial electron transport, NADH to ubiquinone	0.039003451	3.83073115	MT-ND4, MT-ND5, MT-ND2, MT-ND3, MT-ND1
regulation of microtubule cytoskeleton organization	0.040010746	3.145231892	PRKAA2, TAOK1, DIXDC1, PHLDB2, MARK2, RHOA
cellular response to nerve growth factor stimulus	0.040010746	3.145231892	MICALL1, ID1, CSNK1E, CBL, FOXO3, CRK
negative regulation of cardiac muscle cell apoptotic process	0.040010746	3.145231892	ACOT1, NUPR1, CFLAR, JAK2, NFE2L2, PDK1
ATP metabolic process	0.041369472	2.734090468	ATP6V1A, ATP5E, ATP5A1, ATP6V1B2, ATP1B1, ATP5G1, ATP5J2
positive regulation of MAPK cascade	0.042867738	1.701691626	CD74, WWC1, INSR, ITGA1, PDGFB, IQGAP1, SOD1, PRDX2, MADD, CTNNB1, CD36, IGFBP6, JAK2,
positive regulation of fatty acid biosynthetic process	0.0431982	4.979950495	SREBF1, MLXIPL, ELOVL5, MID1IP1
positive regulation of blood coagulation	0.0431982	4.979950495	PRDX2, CD36, S100A9, NFE2L2
amino acid import across plasma membrane	0.0431982	4.979950495	SLC6A6, SLC6A14, SLC16A2, SLC7A2
positive regulation of protein binding	0.043520097	2.141914191	EPB41L5, HSP90AB1, EPB41, BAMBI, USP33, LRRK2, AMFR, EIF2AK3, HIP1R, B2M
protein kinase B signaling	0.043606586	2.451667936	FLCN, PIK3CA, AKT2, SESN2, SOX9, GAS6, PIK3C2B, MTM1
positive regulation of vascular endothelial growth factor	0.044031551	3.688852219	C3, BSG, EIF2AK3, RORA, ATF4
cellular response to interleukin-4	0.044031551	3.688852219	XBP1, RPL3, HSP90AB1, FASN, RPLP0
astrocyte differentiation	0.044031551	3.688852219	NFIX, STAT3, HMGA2, VPS54, NR3C1
cellular sodium ion homeostasis	0.044031551	3.688852219	SLC12A2, SCNN1A, SPP1, ATP1A1, ATP1B1
epithelial cell morphogenesis	0.044031551	3.688852219	EPB41L5, CLDN3, POF1B, CDH1, SIPA1L3
regulation of synapse organization	0.044072867	3.06458492	ARF4, TUBB5, TUBA1A, APOE, ETV5, NECTIN1
microtubule-based process	0.044072867	3.06458492	TUBB2A, TUBB5, TUBA1A, DCTN2, DYNLL1, TUBB4B
mitotic G2 DNA damage checkpoint	0.044072867	3.06458492	TAOK3, TAOK1, HMGA2, NOP53, IER3, CDC14B
positive regulation of protein kinase activity	0.044092125	2.269344529	GPRC5B, RASSF2, PIK3CA, LRRK2, PXN, TAB2, IQGAP1, VLDLR, GAS6
positive regulation of epithelial cell proliferation involved in lung morphogenesis	0.04459885	8.537057992	HMGA2, SRSF6, FGFR2
cholesterol import	0.04459885	8.537057992	STARD5, CD36, LDLR
positive regulation of cholesterol storage	0.04459885	8.537057992	LPL, CD36, SREBF2
melanosome localization	0.04459885	8.537057992	MLPH, VPS33B, RAB27A
ATP synthesis coupled electron transport	0.04459885	8.537057992	MT-ND4, MT-ND5, MT-CO2
regulation of skeletal muscle cell differentiation	0.04459885	8.537057992	DDX17, UQCC2, NR1D2
acetyl-CoA biosynthetic process	0.04459885	8.537057992	ACLY, ACSS1, ACAT1
ether lipid biosynthetic process	0.04459885	8.537057992	GNPAT, FASN, AGPS
positive regulation of transcription from RNA polymerase II promoter in response to stress	0.04459885	8.537057992	MUC1, NFE2L1, NFE2L2
regulation of exit from mitosis	0.04459885	8.537057992	CDKN1C, CDC14A, CDC14B
positive regulation of miRNA mediated inhibition of translation	0.04459885	8.537057992	DDX6, STAT3, EIF4G1
lacrimal gland development	0.04459885	8.537057992	IGSF3, SOX9, FGFR2
embryonic retina morphogenesis in camera-type eye	0.04459885	8.537057992	RBP4, HIPK1, LRP6
cerebellar cortex morphogenesis	0.04459885	8.537057992	NFIX, TUBA1A, PCNT
bradykinin catabolic process	0.04459885	8.537057992	MME, CTSH, ECE1
PERK-mediated unfolded protein response	0.04459885	8.537057992	EIF2AK3, ATF4, NFE2L2
lipid particle disassembly	0.04459885	8.537057992	PRKAA2, CHKA, PNPLA2
protein import	0.04459885	8.537057992	LRP2, APOE, CLU
positive regulation of nitric oxide biosynthetic process	0.04486361	2.681511805	HSP90AB1, DDAH2, INSR, AKT2, CD36, JAK2, CLU

embryonic organ development	0.04486361	2.681511805	GDNF, CTNNB1, ARID2, PBX1, FGFR2, BMPR1A, EPN2
sphingolipid metabolic process	0.04486361	2.681511805	FADS3, SFTPB, ASAH1, SPTLC1, ACER3, B4GALNT1, SERINC3
protein N-linked glycosylation	0.04486361	2.681511805	FUT8, KRTCAP2, PMM1, GFPT1, RPN1, MGAT1, ST3GAL1
protein ubiquitination	0.046333	1.416519252	SEL1L, NEDD4L, UBR3, UBE3A, CBL, SH3RF1, SOCS2, UBB, UBR5, RACK1, ELOB, ARIH1, DCAF11, SOCS7, FBXO9, AMFR, MSL2, KLHL21, UBE2A, ZZEF1, PJA2, MIB1, FBXO31, RNF145, PINK1,
response to ischemia	0.046761167	2.414521452	PER2, EGR1, CREBBP, PINK1, NDNF, ARID1B, HK2, CPEB4
activation of cysteine-type endopeptidase activity involved in apoptotic process	0.046934357	2.240977723	DAP, BCL2L11, EIF2AK3, RACK1, HIP1R, CFLAR, JAK2, XDH, S100A9
bone morphogenesis	0.048372912	2.987970297	CITED2, LRP5, SP5, DHRS3, FGFR2, LRP6
negative regulation of Notch signaling pathway	0.048372912	2.987970297	NFKBIA, MEG3, BCL6, KCTD10, ZBTB7A, DLK1
cellular iron ion homeostasis	0.048536239	2.630917243	ATP6V1A, ATP6V1G1, FTL1, SMAD4, TFRC, CYBRD1, SOD1
regulation of protein localization to plasma membrane	0.049405221	3.557107496	VAMP8, RACK1, PIK3R1, LDLRAP1, SPTBN1
DNA methylation	0.049405221	3.557107496	KMT2E, MEG3, KMT2A, EHMT2, GNAS
regulation of epithelial cell proliferation	0.049405221	3.557107496	JAG1, TACSTD2, FGFR2, HMGN1, LRP6
endocrine pancreas development	0.049405221	3.557107496	ANXA1, EIF2AK3, SOX9, CLU, FOXA2
response to cadmium ion	0.049405221	3.557107496	GCLC, NPC1, CAT, SORD, MT-CYTB
cGMP-mediated signaling	0.049405221	3.557107496	NPR1, NPR2, APOE, CD36, AQP1
positive regulation of wound healing	0.049405221	3.557107496	CLDN4, CLDN3, ANXA1, HRAS, ACTG1
regulation of signal transduction	0.050057817	2.378483819	SPRED2, GNAS, GPC3, FURIN, SPRY1, CRK, SH3RF1, MGLL
regulation of transcription, DNA-templated	0.050234483	1.223145736	FOXA1, ZFP445, EHF, CITED2, PHF1, WWC1, JADE1, ARID4B, RORA, NR3C1, BACH1, ZFP809, DBP, CREB3L1, SOX9, CCNL1, ARID2, TRP53INP1, BRD3, NCOA1, SREBF1, BRD2, ASH1L, FOS, FOXP4, SREBF2, ETV5, FOXP1, KAT2B, SFPQ, EWSR1, ATF5, ATF6, ATF4, CEBPA, CASZ1, NFIX, KMT2A, SRF, FOXO3, ZBTB4, LRP6, CREG1, NKX2-1, ZKSCAN17, ZBTB7A, IRX1, EGR1, SMAD1, IRX2, XBP1,
negative regulation of epidermal growth factor receptor signaling pathway	0.050556396	4.687012231	ERRFI1, ITGA1, CBL, PTPRF
fatty acid homeostasis	0.050556396	4.687012231	MLXIPL, XBP1, PRKAA2, APOE
regulation of cholesterol metabolic process	0.050556396	4.687012231	POR, APOE, LDLR, TTC39B
negative regulation of multicellular organism growth	0.050556396	4.687012231	SOCS2, RAI1, ATXN2, GNAS
positive regulation of interleukin-6 production	0.052083843	1.97403443	CD74, XBP1, STAT3, BSG, ZBTB20, LPL, CYBA, MBP, ARHGEF2, CD36, AGER
tumor necrosis factor-mediated signaling pathway	0.052911837	2.915092973	NFKBIA, CDIP1, ACTN4, TRAF1, JAK2, FOXO3
regulation of cell differentiation	0.052911837	2.915092973	BCL6, PLEKHB2, PLEKHB1, CTNNB1, SIK1, SOX9
localization	0.052911837	2.915092973	MUC1, ANXA1, CDC42BPG, ANXA6, ANK3, CDC42BPA
neutrophil chemotaxis	0.052970837	2.18631973	LGALS3, ITGA1, BSG, SPP1, PPIB, PPIA, S100A9, CXCL15, ITGA9
negative regulation of neuron death	0.052970837	2.18631973	PINK1, LRRK2, STAT3, CTNNB1, APOE, AP2B1, CBL, SORL1, CD200
anterior/posterior pattern specification	0.05441806	1.882186014	BTG2, SMAD4, KMT2A, ZBTB16, BHLHE40, LRP5, PDS5A, HIPK1, PBX1, FOXA2, BMPR1A, LRP6
positive regulation of bone resorption	0.05512267	3.434448617	TFRC, SPP1, DLK1, ITGA9, LRP6
lipid storage	0.05512267	3.434448617	BSCL2, CRY2, B4GALNT1, CD36, PNPLA2
proteasome-mediated ubiquitin-dependent protein catabolic	0.055436704	1.643867154	WDR26, CSNK1A1, NEDD4L, UBE2A, AGAP3, ARMC8, PJA2, MTM1, PSMD8, ZNRF2, KCTD10,
axon guidance	0.055501512	1.677457009	MEG3, EPHA4, SEMA4A, SMAD4, USP33, LMTK2, ANK3, ABLIM1, ALCAM, BSG, NKX2-1, EMB,
steroid biosynthetic process	0.057082124	2.309542259	ERG28, HINT2, PRKAA2, TSPO, HMGCS2, HSD17B12, PBX1, CYP51
establishment of protein localization to mitochondrion	0.057523723	7.469925743	PINK1, TSPO, HK2
negative regulation of tubulin deacetylation	0.057523723	7.469925743	BEX4, PRKAA2, FRY
aromatic compound catabolic process	0.057523723	7.469925743	PON3, PON1, EPHX1
positive regulation of Wnt signaling pathway, planar cell polarity pathway	0.057523723	7.469925743	ABL1, GPC3, NKD1
endothelial tube morphogenesis	0.057523723	7.469925743	BSG, CTNNB1, RHOB
maintenance of cell polarity	0.057523723	7.469925743	PDLIM1, DST, ATN1
renal water absorption	0.057523723	7.469925743	AQP3, AQP1, MLLT6
ventricular compact myocardium morphogenesis	0.057523723	7.469925743	CTNNB1, LRP2, BMPR1A
mammary gland duct morphogenesis	0.057523723	7.469925743	LRP5, NR3C1, LRP6
regulation of membrane permeability	0.057523723	7.469925743	CLDN3, PDCD6IP, TJP2
regulation of Cdc42 protein signal transduction	0.057523723	7.469925743	ABCA1, ABL1, APOE
response to hydroperoxide	0.057523723	7.469925743	SP1, JAK2, MT-ND1
negative regulation of endothelial cell differentiation	0.057523723	7.469925743	JAG1, ID1, XDH
antigen processing and presentation	0.05768995	2.845685997	CD74, H2-EB1, H2-DMB1, RAB27A, H2-AA, H2-AB1
metanephros development	0.05768995	2.845685997	IRX1, IRX2, GDNF, CTSH, SPRY1, PDS5A
regulation of cytokinesis	0.05768995	2.845685997	FLCN, KIF13A, KLHL21, CCP110, BIRC6, CALM1
neural precursor cell proliferation	0.05768995	2.845685997	GAK, BTG2, NFIX, NFIA, ZFP503, PCNT
protein targeting	0.05768995	2.845685997	MLPH, GIPC1, ERBIN, RAB27A, SORL1, ZDHHC3
neuromuscular junction development	0.05768995	2.845685997	TMEFF1, RER1, LRRK2, ANK3, UTRN, FGFR2
lipid particle organization	0.058478348	4.426622662	BSCL2, TBC1D20, PPIA, PNPLA2
phospholipid homeostasis	0.058478348	4.426622662	ABCA1, FABP3, ABCA3, STAT3
positive regulation of reactive oxygen species biosynthetic process	0.058478348	4.426622662	RAB27A, CYBA, CD36, FOXO3
cellular copper ion homeostasis	0.058478348	4.426622662	ATP7B, SLC31A1, APLP2, ATOX1
ATP synthesis coupled proton transport	0.058478348	4.426622662	MT-ATP6, ATP5E, ATP5A1, ATP5G1
response to hydrogen peroxide	0.060637042	2.489975248	PPP1R15B, MT-ND5, CAT, UBE3A, CRK, AREG, SOD1
positive regulation of focal adhesion assembly	0.061180809	3.319966997	EPB41L5, ABL1, IQGAP1, S100A10, MAP4K4
membrane organization	0.061180809	3.319966997	GNPAT, HIP1R, CHP1, MBP, TOR1AIP1
regulation of cell morphogenesis	0.061180809	3.319966997	CLDN4, CLDN3, RAC3, CAMSAP1, BCL9L
phospholipid transport	0.06270693	2.779507253	ABCA1, ATP8A1, ABCA3, OSBP, ATP11A, LDLR
iron ion homeostasis	0.06270693	2.779507253	FECH, EPAS1, CYBRD1, FBXL5, STEAP2, B2M
thymus development	0.065033824	2.446291471	PRDX2, BCL2L11, CITED2, SRF, ABL1, CTNNB1, PBX1
positive regulation of protein localization to plasma membrane	0.065033824	2.446291471	LGALS3, RER1, MYO5B, RAB11FIP2, EZR, SPTBN1, PDK1
cellular response to tumor necrosis factor	0.066161668	1.761615141	CEBPA, ASAH1, LRRK2, ERBIN, CYBA, RORA, PRPF8, ZFP36L2, FOXP1, NFKBIA, ZFP36, CHIL1,
negative regulation of cell death	0.066270123	1.88894674	HSPA9, SMAD4, NPC1, STAT3, ADNP2, TMEM109, JAK2, SRSF6, CLU, RHOA, NFE2L2
regulation of inflammatory response	0.06683947	1.972257622	ANXA1, BCL6, SCGB1A1, NR1D2, JAK2, AGER, SLC7A2, NFE2L1, MGLL, FOXP1
phagocytosis	0.06683947	1.972257622	NFIX, ANXA1, PIK3CA, ANXA3, ABL1, RAB11FIP2, GAS6, LDLR, MET, SYT7
negative regulation of beta-amyloid formation	0.066944512	4.193642522	APOE, SORL1, CLU, RTN4
triglyceride catabolic process	0.066944512	4.193642522	LIPH, LPL, MGLL, PNPLA2
cyclic nucleotide biosynthetic process	0.066944512	4.193642522	NPR1, NPR2, ADCY7, ADCY6
androgen receptor signaling pathway	0.066944512	4.193642522	DDX17, ZMIZ1, UBE3A, RHOA
negative regulation of cell-matrix adhesion	0.066944512	4.193642522	JAG1, BCL6, PIK3R1, MUC4

Biology Open • Supplementary information

regulation of protein binding	0.067575327	3.212871287	HDAC5, APLP2, LDLRAP1, CRK, HOPX
protein sumoylation	0.067575327	3.212871287	RANBP2, CBX4, ZMIZ1, SUMO2, PIAS1
neuron projection development	0.06915825	1.659983498	BTG2, SRF, ECE1, FRY, AGER, AREG, CAMSAP1, DIAPH1, GDNF, CDH1, CLMN, MICALL1, RAC3,
negative regulation of fat cell differentiation	0.06961242	2.404114032	JAG1, SORT1, RORA, ZFP36L2, NUCB2, DLK1, LRP6
negative regulation of sequence-specific DNA binding transcription factor activity	0.070159567	2.06066917	PURA, MED13, PAXIP1, BHLHE40, ID1, NUPR1, GAS6, PBX1, FOXA2
neural tube closure	0.07021568	1.952921763	BRD2, CECR2, RPS7, MTHFD1, CITED2, ABL1, COBL, LRP2, PFN1, LRP6
chitin catabolic process	0.071555378	6.639933993	CHIA1, CHIL1, CHIL3
vascular wound healing	0.071555378	6.639933993	NPR2, NDNF, ADIPOR2
negative regulation of renal sodium excretion	0.071555378	6.639933993	ANPEP, GNAS, COMT
forebrain radial glial cell differentiation	0.071555378	6.639933993	NFIX, RHOA, LRP6
intestinal cholesterol absorption	0.071555378	6.639933993	NPC1, CD36, LDLR
regulation of type B pancreatic cell proliferation	0.071555378	6.639933993	ERRFI1, NR4A1, SGPP2
positive regulation of mitochondrial outer membrane permeabilization involved in apoptotic signaling pathway	0.071555378	6.639933993	ATPIF1, HIP1R, CHCHD10
fat pad development	0.071555378	6.639933993	UBB, ARRDC3, HMGA2
cellular response to oxygen-glucose deprivation	0.071555378	6.639933993	CBL, AQP3, ATF4
circadian behavior	0.071555378	6.639933993	2510009E07RIK, NR1D2, CSNK1E
cerebellum morphogenesis	0.071555378	6.639933993	GNPAT, ABL1, LRP6
store-operated calcium entry	0.071555378	6.639933993	CRACR2A, CRACR2B, STIM1
endoplasmic reticulum organization	0.073453147	2.655973597	GAK, SEC61A1, LRRK2, EIF2AK3, USE1, RTN4
phosphatidylinositol phosphorylation	0.073453147	2.655973597	SOCS2, SMG1, PIK3CA, PIK3R1, SOCS7, PIK3C2B
cardiac muscle cell proliferation	0.074300778	3.112469059	SMAD1, CTNNB1, FGF1, ARID2, FGFR2
embryonic placenta development	0.074300778	3.112469059	CEBPA, CITED2, SP1, EPAS1, PDGFB
neuron death	0.075933116	3.983960396	LRRK2, ABL1, VPS54, CBL
fatty acid oxidation	0.075933116	3.983960396	POR, ACOX1, CD36, ADIPOR2
somatic stem cell population maintenance	0.077190116	2.153492106	LDB1, STAT3, LRP5, HMGA2, SOX9, ZFP36L2, BCL9L, BMPR1A
response to nutrient levels	0.077190116	2.153492106	CPT1A, GSTA3, INSR, PON1, SORD, CYBA, ATF4, SOD1
positive regulation of translation	0.077886231	2.014361998	RPL5, PINK1, DDX3X, LARP1, LARP4B, EEF2, EIF4G3, EIF4G2, RBMS3
negative regulation of neuron projection development	0.077886231	2.014361998	GAK, EPHA4, LRRK2, APOE, B2M, RTN4, ADCY6, RHOA, MAP4K4
histone modification	0.07917875	2.598235041	KDM6B, MEG3, CREBBP, KMT2A, BRPF3, JADE1
cellular response to cytokine stimulus	0.07917875	2.598235041	NFKBIA, MME, STAT3, NFKBIZ, LDLRAP1, RHOA
epithelial to mesenchymal transition	0.07917875	2.598235041	DDX17, EPB41L5, SMAD4, HMGA2, SOX9, FGFR2
positive regulation of T cell differentiation	0.07917875	2.598235041	CD74, XBP1, ZMIZ1, ARID2, H2-AA, ARID1B
macromolecular complex assembly	0.07924103	1.825981848	VAMP8, CD74, HACD1, KMT2A, PPP2R1A, EPB41, MGP, CHCHD10, NUPR1, SOX9, TAPBP
protein destabilization	0.079313173	2.323976898	DAZAP2, XBP1, CREBBP, DAP, ID1, CTSH, SERF2
positive regulation of protein tyrosine kinase activity	0.081350665	3.018151815	EPHA4, GPRC5B, DLG3, PDGFB, GAS6
lysosome localization	0.081350665	3.018151815	FLCN, RAB3A, VPS33B, KIF1B, ARL8B
intrinsic apoptotic signaling pathway in response to DNA damage by p53 class mediator	0.081350665	3.018151815	DYRK2, CDIP1, TMEM109, NUPR1, HIPK1
triglyceride homeostasis	0.081350665	3.018151815	MED13, SESN2, LPL, RORA, APOE
erythrocyte development	0.081350665	3.018151815	BCL6, CITED2, SRF, RPS6, ADGRF5
positive regulation of mitotic nuclear division	0.081350665	3.018151815	RGCC, INSR, LRP5, PDGFB, MET
response to estradiol	0.08476565	1.879226602	NCOA1, SOCS2, FOXA1, ANXA1, CCND1, MMP15, STAT3, CAT, CTNNB1, AREG
spleen development	0.085135995	2.542953444	BCL2L11, KMT2A, CITED2, NFKBIZ, ABL1, PBX1
positive regulation of protein serine/threonine kinase activity	0.085135995	2.542953444	DAZAP2, DDX3X, HSP90AB1, CAB39, HMGA2, RHOA
negative regulation of smooth muscle cell proliferation	0.085135995	2.542953444	NPR1, ANG, PIK3R1, APOE, COMT, SF1
intermediate filament cytoskeleton organization	0.085420565	3.794247996	SYNM, DST, CSNK1A1, PPL
nuclear migration	0.085420565	3.794247996	DYNC1H1, CLMN, CDC42BPA, SYNE2
positive regulation of lipid biosynthetic process	0.085420565	3.794247996	SREBF1, MLXIPL, ZBTB20, APOE
chromatin remodeling	0.08613557	1.640454281	FOXA1, KDM6B, BRD3, BRD2, INO80D, NR3C1, ARID1B, CECR2, KAT2B, RYBP, SOX9, ARID2,
hyperosmotic salinity response	0.086550532	5.975940594	NKX2-1, MT-CYTB, AQP1
hydrogen peroxide biosynthetic process	0.086550532	5.975940594	ACOX1, CYBA, SOD1
negative regulation of ubiquitin protein ligase activity	0.086550532	5.975940594	RPL5, RPS7, RPL23
coronary artery morphogenesis	0.086550532	5.975940594	CTNNB1, LRP2, ARID2
CRD-mediated mRNA stabilization	0.086550532	5.975940594	CSDE1, IGF2BP1, IGF2BP3
endothelial cell activation	0.086550532	5.975940594	SMAD4, PPIA, FOXP1
negative regulation of circadian rhythm	0.086550532	5.975940594	PER2, SFPQ, CRY2
long-chain fatty-acyl-CoA biosynthetic process	0.086550532	5.975940594	ELOVL5, ACSL5, ACSL4
negative regulation of muscle cell differentiation	0.086550532	5.975940594	SMAD1, TNPO2, BMPR1A
embryonic process involved in female pregnancy	0.086550532	5.975940594	CITED2, SP1, ACSL4
ectoderm development	0.086550532	5.975940594	EPB41L5, CTNNB1, BMPR1A
dorsal/ventral axis specification	0.086550532	5.975940594	CTNNB1, BMPR1A, LRP6
endothelial cell apoptotic process	0.086550532	5.975940594	COL18A1, ID1, HIPK1
positive regulation by host of viral genome replication	0.086550532	5.975940594	TBC1D20, NUCKS1, PPIB
autophagy	0.086661758	1.570033654	XBP1, PRKAA2, TMEM41B, LRRK2, VAMP8, PINK1, DAP, DRAM1, NPC1, STBD1, CREG1, UBQLN1,
response to electrical stimulus	0.088717528	2.929382644	NR4A1, XBP1, BTG2, RAB3A, MT-CO1
determination of adult lifespan	0.088717528	2.929382644	MEG3, LRRK2, ATN1, B4GALNT1, TFCP2L1
long-chain fatty acid metabolic process	0.088717528	2.929382644	CPT1A, ACOT1, ACSL5, ACSL4, CD36
decidualization	0.088717528	2.929382644	VDR, CITED2, CDH1, BSG, ASH1L
mitochondrial respiratory chain complex I assembly	0.0897322	2.249009901	MT-ND4, MT-ND5, NDUFA6, NDUFB3, NDUFA1, MT-ND2, MT-ND1
neuron migration	0.0899679	1.670693069	SRF, NDNF, SH3RF1, RHOA, TUBB2A, TUBA1A, NKX2-1, CTNNB1, PCNT, GAS6, CRK, MET, MARK2
hippocampus development	0.090391544	1.948676281	KDM6B, BCAN, NFIX, TUBA1A, ANXA3, SRF, OGDH, NKX2-1, CRK
regulation of cell migration	0.093674501	1.767079208	GNA13, BEX4, LDB1, TMSB4X, OSGIN1, FGF1, TMSB10, RTN4, RHOA, PLXNA4, RHOB
hemopoiesis	0.094802974	1.927722772	FLCN, PINK1, EPAS1, ZBTB16, MKNK2, CTNNB1, CRIP2, ZFP36L2, CXCL15
positive regulation of cell death	0.095207428	2.213311331	EGR1, DAP, BCL2L11, HP, CD36, KCNK2, EIF4G1
positive regulation of chondrocyte differentiation	0.095381812	3.621782178	POR, ZBTB16, SOX9, BMPR1B
regulation of protein ubiquitination	0.095381812	3.621782178	PINK1, HSP90AB1, UBQLN1, HERPUD1
negative regulation of protein secretion	0.095381812	3.621782178	ANXA1, RHBDF2, APOE, CYP51
positive regulation of dendrite development	0.095381812	3.621782178	ABL1, COBL, IQGAP1, VLDLR
mesenchymal cell proliferation	0.095381812	3.621782178	CTNNB1, SOX9, FGFR2, LRP6
TOR signaling	0.095381812	3.621782178	FLCN, LARP1, RPS6, MTM1

Biology Open • Supplementary information

ribosomal small subunit biogenesis	0.095381812	3.621782178	RPS16, RPS7, RPS6, RPS24
myotube differentiation	0.095381812	3.621782178	HACD1, STIM1, SORT1, MET
motor behavior	0.095381812	3.621782178	TUBA1A, VPS54, B4GALNT1, NR3C1
positive regulation of endocytosis	0.096393021	2.845685997	GPC3, LRP2, APOE, AP2B1, MIB1
positive regulation of DNA binding	0.096393021	2.845685997	TXN1, EDF1, JAK2, CALM1, HIPK1
neuron projection extension	0.096393021	2.845685997	BCAN, NRN1, ALCAM, CTNNB1, IQGAP1
response to tumor necrosis factor	0.096393021	2.845685997	TUBA1A, INSR, CHIL1, MAP2K7, JAK2
embryonic hindlimb morphogenesis	0.096393021	2.845685997	ZBTB16, GNAS, GPC3, CTNNB1, LRP6
positive regulation of canonical Wnt signaling pathway	0.096776546	1.827504769	PPM1B, GPRC5B, DDX3X, BAMBI, LRRK2, UBR5, GPC3, XIAP, CSNK1E, FGFR2
animal organ regeneration	0.097732191	2.439159426	PRPS2, CCND1, ANXA3, LIFR, MT-CYTB, GAS6
negative regulation of I-kappaB kinase/NF-kappaB signaling	0.097732191	2.439159426	PER1, PPM1B, ABL1, RORA, ASH1L, RHOA
T cell differentiation in thymus	0.097732191	2.439159426	NCOR1, FZD5, RPS6, CTNNB1, B2M, ZFP36L2
eukaryotic translation initiation factor 4F complex assembly	0.097790381	19.91980198	EIF4H, EIF4B
negative regulation of mitophagy	0.097790381	19.91980198	PINK1, TSPO
positive regulation of metalloendopeptidase activity	0.097790381	19.91980198	STAT3, MBP
lung smooth muscle development	0.097790381	19.91980198	SRF, SOX9
regulation of centromere complex assembly	0.097790381	19.91980198	H3F3B, H3F3A
negative regulation of cellular respiration	0.097790381	19.91980198	FLCN, TRAP1
positive regulation of dopaminergic neuron differentiation	0.097790381	19.91980198	FOXA1, FOXA2
canonical Wnt signaling pathway involved in positive regulation of cardiac outflow tract cell proliferation	0.097790381	19.91980198	CTNNB1, LRP6
negative regulation of mitochondrion organization	0.097790381	19.91980198	FLCN, TSPO
postganglionic parasympathetic fiber development	0.097790381	19.91980198	GDNF, PLXNA4
positive regulation of ARF protein signal transduction	0.097790381	19.91980198	MAPRE2, MAP4K4
positive regulation of monooxygenase activity	0.097790381	19.91980198	POR, GDNF
cellular response to mycophenolic acid	0.097790381	19.91980198	EGR1, PDGFB
proximal/distal pattern formation involved in metanephric nephron development	0.097790381	19.91980198	IRX1, IRX2
aerobic electron transport chain	0.097790381	19.91980198	SLC25A51, MT-CO3
tarsal gland development	0.097790381	19.91980198	ASH1L, SCD1
response to caloric restriction	0.097790381	19.91980198	APOE, LDLR
positive regulation of neurofibrillary tangle assembly	0.097790381	19.91980198	APOE, CLU
positive regulation of activation of membrane attack complex	0.097790381	19.91980198	C3, C6
negative regulation of matrix metallopeptidase secretion	0.097790381	19.91980198	IDH2, CD200
regulation of female gonad development	0.097790381	19.91980198	INSR, NUPR1
negative regulation of chromosome condensation	0.097790381	19.91980198	H3F3B, H3F3A
negative regulation of vasculogenesis	0.097790381	19.91980198	XDH, RTN4
negative regulation of lung goblet cell differentiation	0.097790381	19.91980198	FOXP4, FOXP1
regulation of protein sumoylation	0.097790381	19.91980198	EGR1, ZMIZ1
cranial nerve morphogenesis	0.097790381	19.91980198	CITED2, PLXNA4
neuropeptide catabolic process	0.097790381	19.91980198	CTSH, LNPEP
negative regulation of intrinsic apoptotic signaling pathway in response to hydrogen peroxide	0.097790381	19.91980198	TRAP1, PINK1
bronchus cartilage development	0.097790381	19.91980198	SRF, SOX9
aflatoxin catabolic process	0.097790381	19.91980198	GSTA3, NFE2L2
lung secretory cell differentiation	0.097790381	19.91980198	FOXP4, FOXP1
intracellular lipid transport	0.097790381	19.91980198	FABP3, TMEM41B
cytosolic lipolysis	0.097790381	19.91980198	BSCL2, CES1D
regulation of lung goblet cell differentiation	0.097790381	19.91980198	FOXP4, FOXP1
complement-dependent cytotoxicity	0.097790381	19.91980198	C3, RAB27A
negative regulation of translation in response to endoplasmic reticulum stress	0.097790381	19.91980198	SESN2, EIF2AK3
positive regulation of growth factor dependent skeletal muscle satellite cell proliferation	0.097790381	19.91980198	STAT3, JAK2
positive regulation of glutathione biosynthetic process	0.097790381	19.91980198	EIF2AK3, NFE2L2

Biology Open • Supplementary information
